# Asymmetric Synthesis of the HIV Protease Inhibitor TMC-126, a PMX Antimalarial Protease Inhibitor, and a Putative COVID-19 Inhibitor Using a Highly Stereoselective Glycolate Aldol Addition Reaction Pathway

**DOI:** 10.1021/acsomega.6c01499

**Published:** 2026-05-01

**Authors:** Kweku Amaning Affram, Austin Carter, April Breede, Alexandria Kimsey, Godson Hemeson, Bader Semakieh, Moses Martinez, Emmanuel Ayim, Joy Odeh, Shawn R. Hitchcock

**Affiliations:** Department of Chemistry, 6049 Illinois State University, Normal, Illinois 61790-4160, United States

## Abstract

An asymmetric glycolate aldol addition pathway was developed for the synthetic preparation of a series of protease inhibitors based on the hydroxyethylamine structural motif. A single glycolate aldol adduct derived from a highly diastereoselective (≥95:5 d.r.) asymmetric aldol reaction served as the starting point for the synthesis of the inhibitors. The starting material is easily prepared and purified via recrystallization on multigram scales. This work describes the synthesis of the HIV protease inhibitor TMC-126, the Plasmepsin X (PMX) inhibitor **49c** for the treatment of malaria, and a computationally derived inhibitor for the 3CLpro of the SARS-CoV-2 from a single common synthetic intermediate.

## Graphical abstract

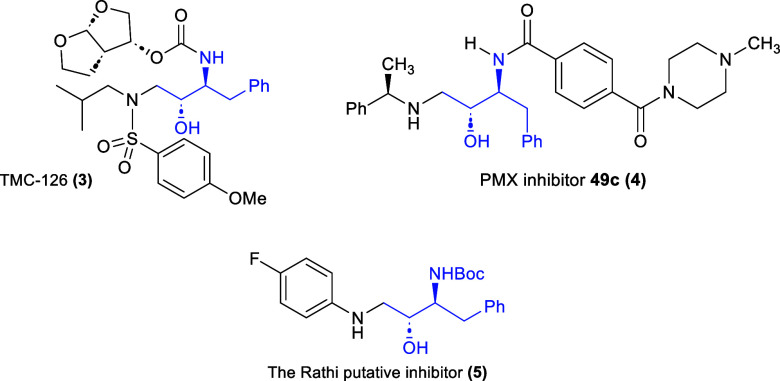

## 1 Introduction

The asymmetric glycolate aldol addition reaction developed by Crimmins and co-workers^1–3^ has been exploited as a useful synthetic tool in the preparation of a variety of biologically active substrates including gigantecin,^4^ pyranicin,^5^ (+)-laurencin,^6,7^ mucocin,^8^ and bistramide A.^9^ We recently employed the asymmetric glycolate aldol addition in the synthesis of the HIV protease inhibitor darunavir **(1)** ^10^ using a *N*-glycolate oxazolidine-2-thione aldol adduct **(2)**. Due to the nature of the substitution of the glycolate aldol adduct **2** and its stability to stereochemical epimerization, it can be used as a key synthon for the preparation of substrates that possess the hydroxyethyl isostere template that has proven to be useful in a wide variety of medicinal agents that block enzyme action (Figure 1).^11,12^ We became interested in determining if it might be possible to use this methodology in the synthesis of other medicinally valuable synthetic targets. To this end, the HIV protease inhibitor TMC-126 **(3)**,^13^ the antimalarial PMX inhibitor “**49c**” **(4)**,^14^ and a computationally derived protease inhibitor **(5)** developed by Rathi and co-workers^15^ were considered as viable candidates for synthesis.

**Figure 1:**
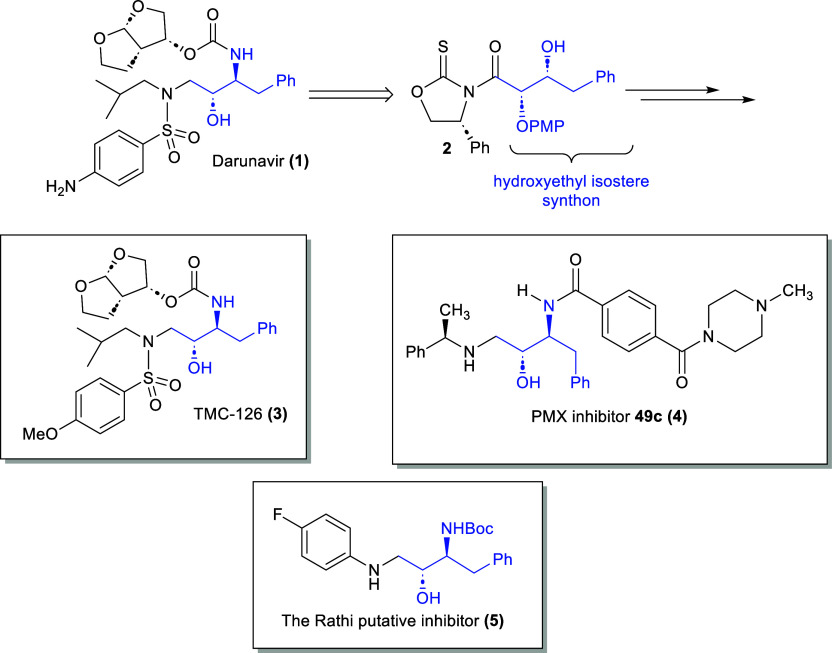
Candidates for synthesis via the asymmetric glycolate addition reaction.

TMC-126 **(3)**, the precursor to darunavir, and also referred to as UIC-94003, is a nonpeptidic HIV antiretroviral medication that is a second-generation HIV type-1 protease inhibitor that was originally designed to address the challenges of drug resistance.^16^ TMC-126 became a target of interest as we wanted to demonstrate the flexibility of the synthetic methodology involving the glycolate aldol starting material **2** that had been previously employed in the synthesis of darunavir. There was also an interest in pursuing the synthesis of the antimalarial Plasmepsin X (PMX) inhibitor **49c (4)**. It is a peptidomimetic inhibitor of the Plasmepsin X aspartic protease of *Plasmodium falciparum*, the parasite that causes the most severe form of malaria.^17,18^ The active site of the protease is characterized by a pocket region that accommodates inhibitors that belong to the hydroxyethyl isostere family, which can interact with the aspartic residues and other active site features.^14,19,20^ The PMX inhibitor **49c (4)** is a hydroxyethyl isostere based inhibitor and so we became interested in employing the glycolate aldol adduct **2** in its synthetic preparation. Finally, Mukherjee and co-workers^15^ utilized computational docking simulations to evaluate a series of potential drug candidates for inhibition of the SARS-CoV-2 protease (3CL^pro^) associated with Covid-19 pandemic.^21^ Among the viable candidates, indinavir, an early HIV protease inhibitor possessing the hydroxyethylamine isostere motif, demonstrated the strongest docking and binding affinity. A library of compounds possessing this motif were screened and among these select molecules would emerge the putative protease inhibitor **5**. While this compound has not entered clinical trials as of yet, it was still an attractive for synthesis via the glycolate chemistry. Herein, we describe the synthesis of TMC-126 **(3)**, the PMX inhibitor **49c (4)**, and a proposed Covid-19 protease inhibitor **(5)** using an asymmetric glycolate aldol addition pathway.

## 2 Results and Discussion

It was envisioned that the protease inhibitor TMC-126 could be synthesized in a similar manner as the earlier preparation darunavir via the asymmetric glycolate aldol addition synthetic pathway. The key difference in the synthetic pathways would then be the introduction of the *N*-*p*-methoxybenzenesulfonamide group in TMC-126 rather than that of the *N*-*p*-nitrobenzenesulfonamide group in darunavir (Scheme 1). Thus, retrosynthetically, TMC-126 was proposed to be derived from β-hydroxy-γ-amide sulfonamide **6** via hydrogenation and *N*-acylation. Sulfonamide 6 was considered to be accessible from the PMP protected sulfonamide **7** which in turn would originate from γ-amino alcohol **8** via bis­(sulfonylation). Finally, alcohol **8** would be derived from the glycolate aldol addition starting material.

**Scheme 1:**
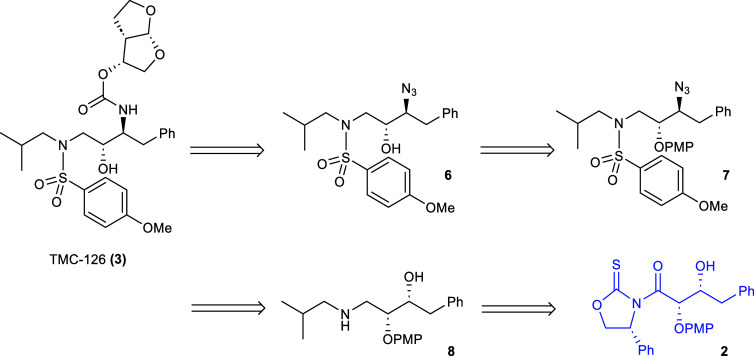
Retrosynthetic Approach to Preparation of TMC-126

The asymmetric synthesis of TMC-126 began with treatment of the glycolate aldol addition product **2** ^10^ was with an excess of isobutylamine in the presence of stoichiometric amounts of imidazole (nucleophilic acylation catalyst) to form the *N*-isobutylamide **9** in quantitative yield via transamidation (Scheme 2). This material was then reduced using sodium borohydride and iodine (I_2_)^22^ to yield γ-aminoalcohol **8** in 90% yield. The amine was then doubly activated with two equivalents of the *p*-methoxyphenylsulfonyl chloride and DMAP in dichloromethane. This process afforded the *O*-*p*-methoxysulfonyl-*N*-sulfonamide **10** in 51% yield after chromatographic purification and recrystallization. Reaction of sulfonamide **10** with sodium azide in DMSO overnight at 60 °C gave γ-azido sulfonamide **7** in near quantitative yield after flash chromatography.

**Scheme 2:**
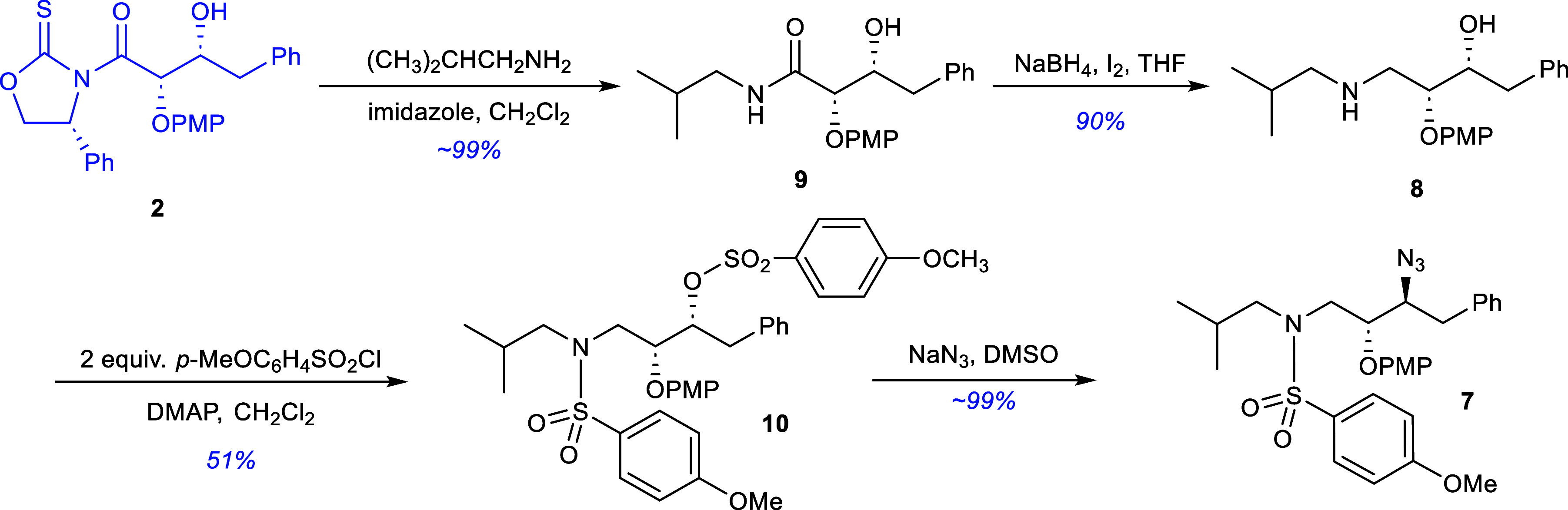
Synthesis of the Key Azide Intermediate **7**

The penultimate step in the synthesis of the target protease inhibitor was the chemoselective deprotection of the *p*-methoxyphenoxy group with ceric ammonium nitrate^23^ (Scheme 3). We were pleased to determine that the deprotection could be accomplished in 60 min to afford β-hydroxy-*N*-sulfonamide **6** in 51% yield after chromatographic purification. While the isolated chemical yield was not ideal, the removal of the *p*-methoxyphenoxy (PMP) group in the presence of the *p*-methoxyphenylsulfonyl group was achieved. TMC-126 was finally prepared by hydrogenation of *N*-sulfonamide **6** in the presence of 10% palladium on carbon and the *N*-hydroxy-succinimidyl *O*-bis­(THF) carbonate **11** followed by chromatographic purification. We were pleased with the overall synthesis of TMC-126 as a starting point in terms of exploring the flexibility of using the asymmetric glycolate aldol addition reaction pathway as an entry point into medicinally important substrates bearing the hydroxyethyl isostere structural motif. From this point, the synthesis of the PMX inhibitor “**49c**” **(4)**.

**Scheme 3:**
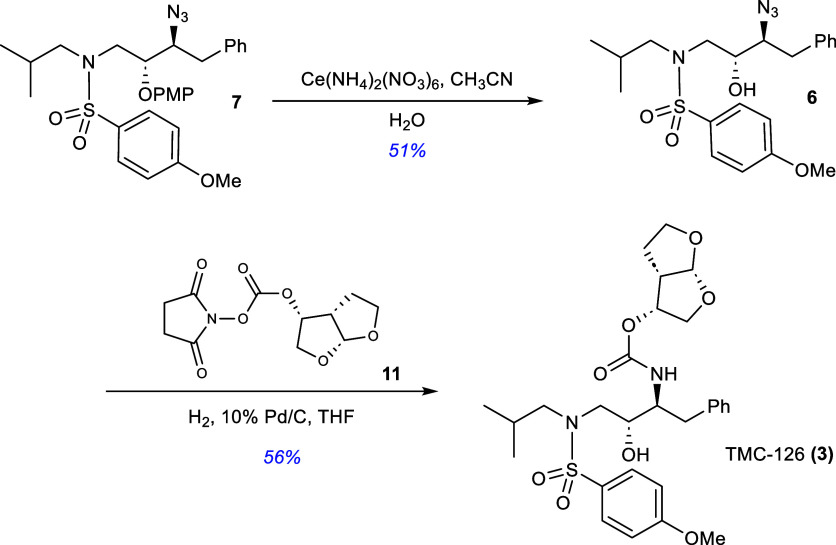
Synthesis of TMC-126 **(3)**

The retrosynthetic planning for the PMX inhibitor **49c (4)** was considered to be divergent from the pathway for darunavir and TMC-126 as there was no sulfonamide group present, meaning that the synthesis would have to take an unprotected secondary amine into account (Scheme 4). In terms of the planning for the synthetic preparation of the PMX inhibitor **49 (4)**, it was proposed that the final step in the synthesis would be the deprotection of the PMP group in γ-amino amide **12**. This compound would originate from a chemoselective amide coupling of the γ-diamine **14** with terephthalic acid derivative **13**. The γ-diamine **14** would be sourced from β-azidoamide **15** through hydride reduction. Finally, amide **15** would be obtained via a transamidation of the glycolate aldol addition adduct **2**.

**Scheme 4:**
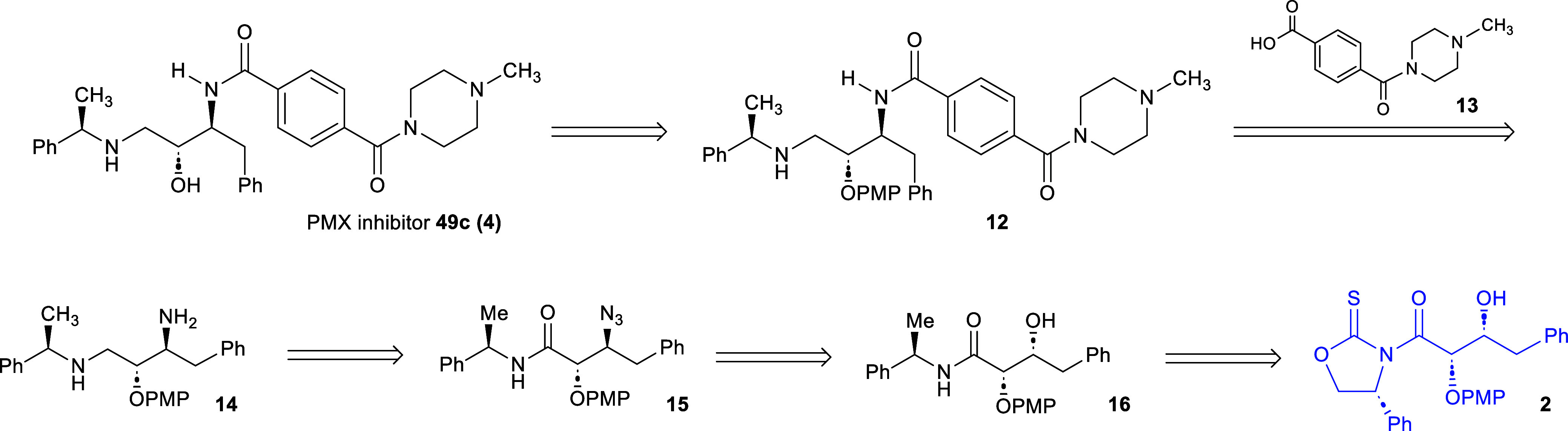
Retrosynthetic Analysis of PMX Inhibitor **49c**

With the planning established, glycolate aldol adduct **2** was reacted with (*R*)-1-phenethylamine in the presence of imidazole and dichloromethane to afford the transamidation product **16** in 85% after recrystallization (Scheme 5). The product **(16)** was then treated with *p*-nitrobenzenesulfonyl chloride and a catalytic amount of DMAP in dichloromethane to afford the *p*-nosylated amide **17** in 88% yield after recrystallization. Treatment of amide **17** with sodium azide in DMSO at 50 °C for 48 h yielded the corresponding β-azidoamide **15** in 71% yield after recrystallization. Fortuitously, there was little evidence for the formation of the potential elimination product of **17**, namely the α,β-unsaturated amide **18**.

**Scheme 5:**
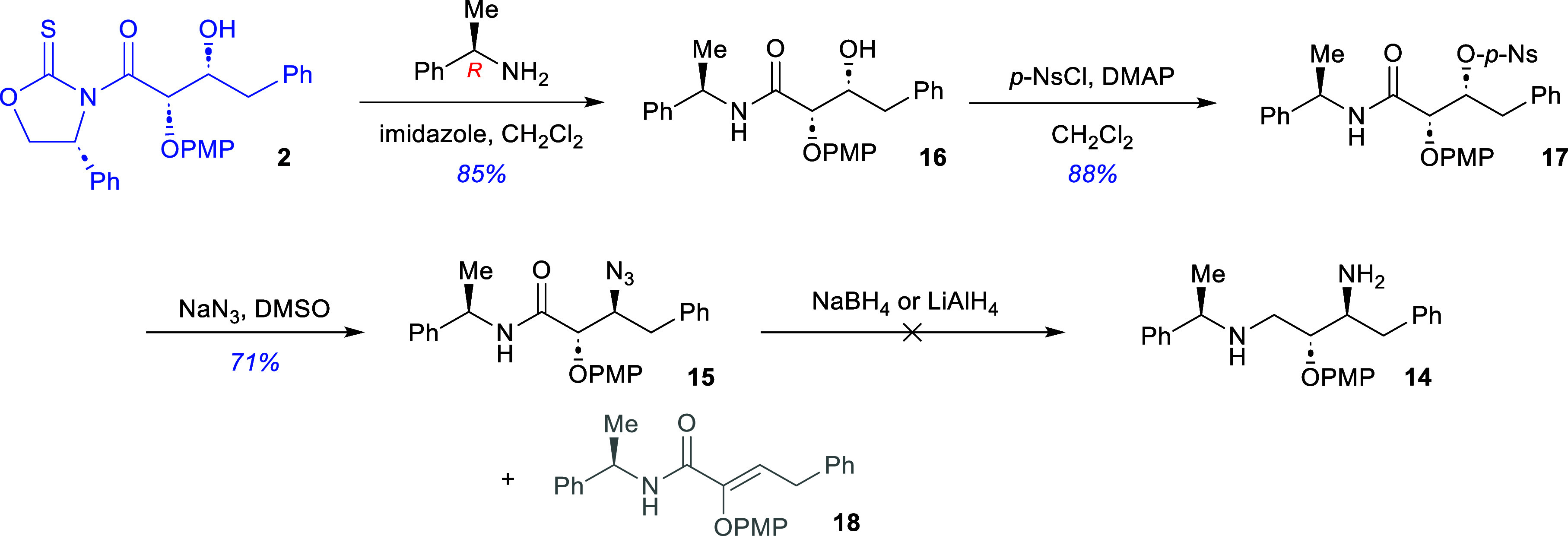
Initial Efforts towards the Synthesis of Intermediate **13**

With compound **15** in hand, we conducted a series of attempts to reduce the azido functionality and the amide carboxyl group using a variety of reducing agents including sodium borohydride with iodine, borane–dimethylsulfide complex, and lithium aluminum hydride. All attempts to induce the reduction of this substrate failed to generate the desired product **14** as an isolable material. To circumvent the difficulties associated with the reduction of β-azidoamide **15**, the PMP protecting group was removed (Scheme 6). It was anticipated that the free alcohol would allow the reducing agent to bind to the substrate and allow for a more effective reduction. Thus, treatment of **15** with ceric ammonium nitrate in a solvent mixture of acetonitrile and water (5:1) yielded the deprotected amide **19** in 97% yield after chromatographic purification. When the β-azido-α-hydroxyamide **19** was reacted with lithium aluminum hydride, the γ-diamine **14** was obtained in 67% yield after flash chromatographic purification. At this stage, monomethyl terephthalic acid **(20)** was converted to its corresponding amide **(21)** by reaction with thionyl chloride followed by *N*-methylpiperidine in 65% yield after chromatographic purification. The benzoate ester functionality of amide **21** was then saponified with methanolic sodium hydroxide in dichloromethane to provide the terephthalic acid derivative **13**.^24^

**Scheme 6:**
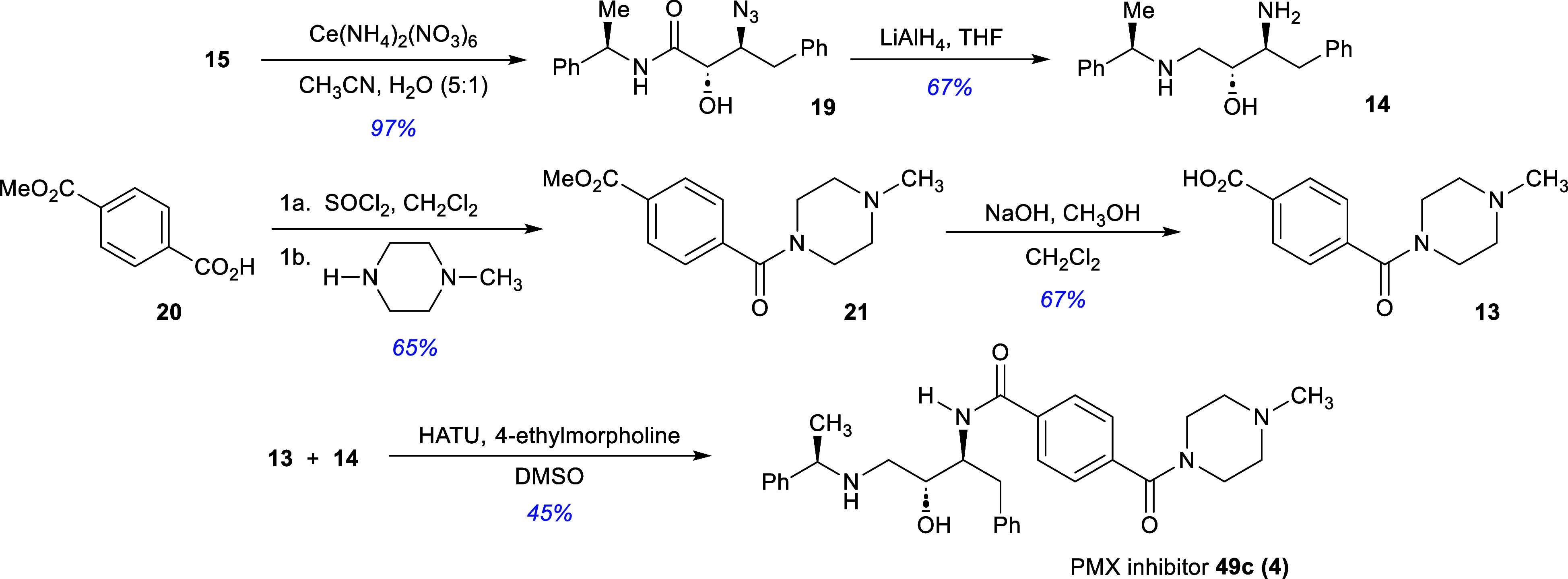
Synthesis of the PMX Inhibitor **49c (4)**

With the key components having been prepared, the synthesis of the PMX inhibitor **49c (4)** was concluded with the reaction of γ-diamine **14** with the terephthalic acid derivative **13**. Coupling of these two fragments with HATU and *N*-methylmorpholine afforded the target inhibitor in 45% after chromatographic purification.

We were also interested in pursuing the synthesis of the computationally derived putative Covid-19 inhibitor **(5)** developed by Rathi and co-workers (Scheme 7). It is proposed that the target will be formed by the chemoselective Boc protection of the primary amine group in **22**. Based on the knowledge gained from the PMX inhibitor synthesis, γ-diamine **22** will be derived from β-hydroxy-α-hydroxyamide **23**. In turn, this material will be obtained from the *N*-*p*-fluorophenylamide **24** via a process of *p*-nosylation and azidation. Amide **24** will be obtained from the glycolate starting material.

**Scheme 7:**
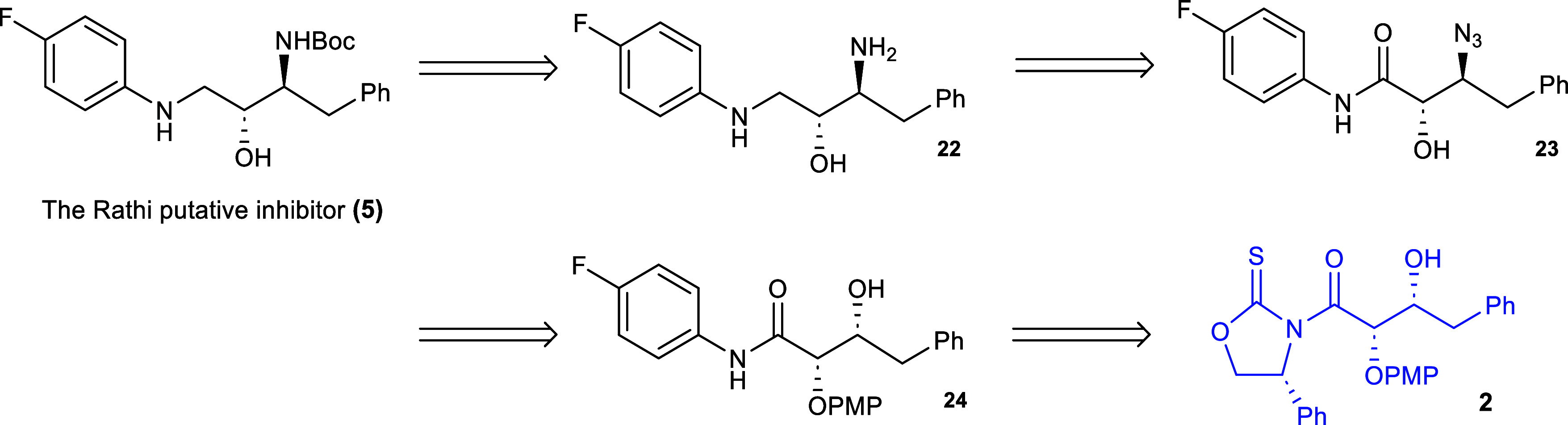
Retrosynthetic Evaluation of the Rathi Putative Inhibitor **(5)**

The synthesis of the inhibitor was launched by the transamidation reaction of the glycolate aldol adduct **2** with *p*-fluoroaniline in the presence of imidazole and dichloromethane (Scheme 8). Interestingly, the use of an excess of the imidazole (3 equiv) as described by Crimmins and co-workers caused the reaction to generate a number of additional products from which the transamidation product **24** could only be obtained in 26% yield. However, when the amount of the imidazole was reduced to substoichiometric amounts, the isolated yield after recrystallization was 75%. It is proposed that the use of excessive amounts of imidazoles gives rise to the efficient formation of the acyl imidazole **25** which undergoes coupling with the *p*-fluoroaniline to form *N*-*p*-fluoroamide **24** that undergoes a second coupling to form imide **26**, tentatively identified from the reaction mixture via high resolution mass spectrometry (calculated exact mass: 702.2472; observed exact mass: 702.2460). Amide **24** was subsequently nosylated in 82% yield after recrystallization by reaction with *p*-nitrobenzenesulfonyl chloride and DMAP in dichloromethane. This material was then reacted with sodium azide in DMSO at 50 °C for 48 h to afford the β-azide **28** in 82% yield after recrystallization.

**Scheme 8:**
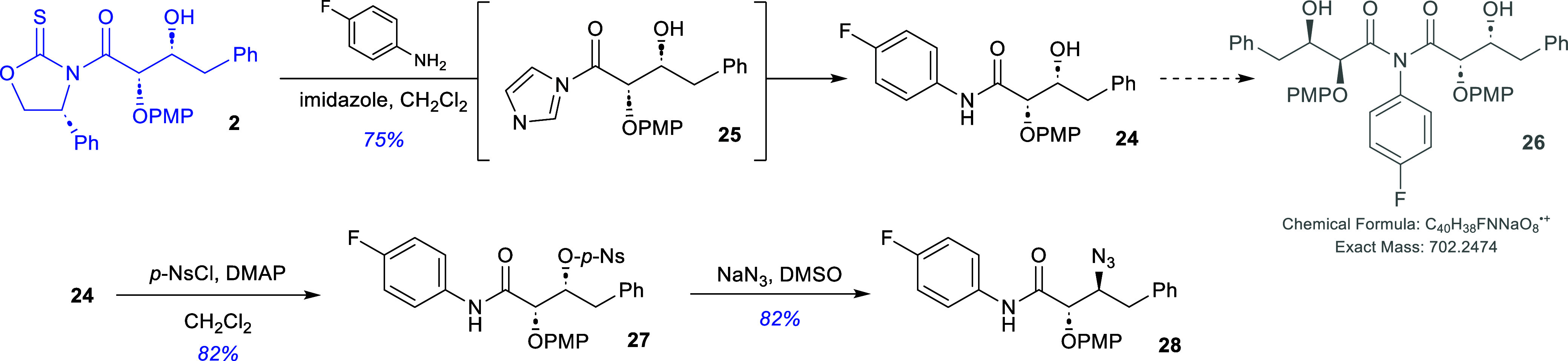
Synthesis of the β-Azidoamide **27**

The β-azidoamide **28** was then deprotected by treatment with ceric ammonium nitrate in acetonitrile and aqueous solution of 2 M sulfuric acid to afford the β-azido-α-hydroxyamide **23** in 34% yield (Scheme 9). This reaction proved to be challenging due to the formation of byproducts among which amide **32** was tentatively assigned based on analysis of the crude reaction mixture by high resolution mass spectrometry (Supporting Information). This byproduct **(32)** could not be separated from other byproducts and thus could not be fully characterized. Prolonged treatment of reaction mixtures containing **32** with aqueous H_2_SO_4_ did not lead to the desired product **23**. Nonetheless, this structure is not unprecedented as Su and co-workers^25^ were able to form similar structures using a palladium­(II) and BF_3_-etherate catalyzed cyclization of alkynyl-tethered aryl ethers.

**Scheme 9:**
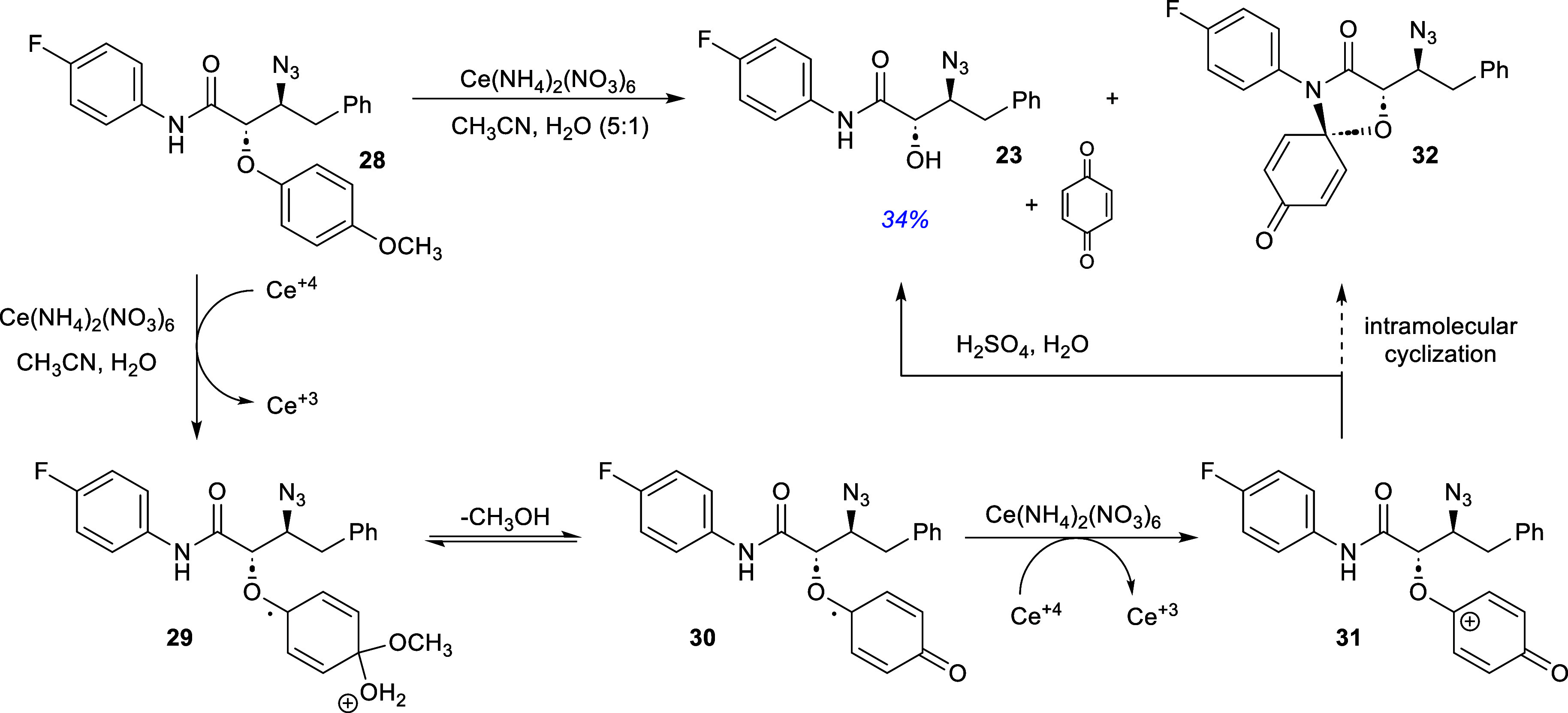
Synthesis of the Rathi Putative Covid-19 Inhibitor **(5)**

It is proposed that the β-azido amide **28** undergoes an initial single electron transfer with the ceric ammonium nitrate and nucleophilic addition to form the first activated intermediate **29**. Subsequent loss of methanol through proton exchange leads to activated intermediate **30** that undergoes a second single electron transfer to putative oxocarbenium ion **31**. This cation can either react with water to undergo loss of benzoquinone or undergo an intramolecular cyclization with the amido nitrogen to yield the tentatively assigned byproduct **32**. Interestingly, subjecting the impure **32** to an aqueous solution of 2 M sulfuric acid did not cause the formation of the desired product **23**. Nonetheless, the recovered product **23** was moved onto the penultimate step by reduction. Amide **23** was subsequently reduced with lithium aluminum hydride in THF to the γ-diamine **22** in 53% yield (Scheme 10). Chemoselective Boc-acylation with *tert*-butoxycarbonyl anhydride yielded the Rathi putative inhibitor in 64% isolated yield after chromatography.

**Scheme 10:**
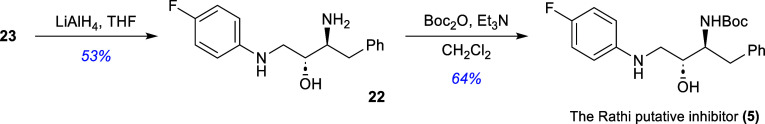
Synthesis of the Rathi Putative Covid-19 Inhibitor **(5)**

## 3 Conclusion

Using the asymmetric glycolate aldol adduct **2**, we have completed the synthesis of TMC-126 **(3)**, the antimalarial PMX inhibitor **49c (4)**, and a proposed protease inhibitor **(5)** of the SARS-CoV-3CLpro protease. The glycolate adduct **2** proved to be a flexible synthon generator for the hydroxyethyl isostere motif. While not as efficient as the methodology of employing phenylalanine derived epoxides^26–30^ commonly employed in the construction of protease inhibitors, the synthetic pathways described here show an alternate route to achieve the synthesis of different inhibitors.

## 4 Experimental Section

### 4.1 Safety Considerations

The use of sodium borohydride in conjunction with iodine gives rise to the generation of borane (BH_3_) in situ at reflux temperatures in tetrahydrofuran. The reaction must be conducted in a well vented fume hood, and an efficient condenser should be used. The use of lithium aluminum hydride requires similar treatment. The quenching of this reaction should be done once the reaction has been cooled to 0–5 °C while allowing for venting of hydrogen gas released. The use of sodium azide in dimethylsulfoxide at ∼50 °C was monitored to ensure a stable temperature. Azides are shock sensitive and should be handled with care and not exposed to metal spatulas, transition metals, or acid sources.

### 4.2 General Experimental Design

Unless otherwise addressed, all reactions were conducted in glassware that had been flame-dried under an inert atmosphere of dry nitrogen gas (continuous flow). The chemical reagents were purchased from MilliporeSigma, Combi-Blocks, and ThermoFisher and were used without further purification. All solvents were used as purchased. Halogenated solvents (dichloromethane and chloroform) were handled in accordance with Federal regulations.^31^ Reactions were heated to reflux temperatures by the use of thermowells controlled by variable transformers. All nucleophilic substitutions involving sodium azide were heated using silicone oil baths heated on hot plates controlled by thermocouple sensors. TLC analyses were carried out using TLC plates coated with silica gel with fluorescent indicator *F*_254_. Flash chromatography was carried out using prepacked silica gel columns and fractions were analyzed by the use of UV lamps (254 nm). Proton NMR spectral data was collected either on a 500 or 400 MHz NMR spectrometer using either deuterated chloroform (CDCl_3_) or deuterated dimethylsulfoxide (DMSO-*d*_6_). Proton decoupled carbon-13 NMR [^13^C­{^1^H}] spectral data was collected at 100 MHz using the 400 MHz ^1^H NMR spectrometer. The associated coupling constants (*J* values) were reported in Hertz (Hz) values and all chemical shifts are reported in parts per million (ppm) in relation to the observed residual chloroform peak (7.26 ppm in ^1^H and 77.2 ppm in ^13^C NMR spectroscopy) and residual DMSO-d_5_ (2.50 ppm in ^1^H and 39.5 ppm in ^13^C NMR spectroscopy). Optical activities were measured on a Jasco P-2000 digital polarimeter. Mass spectral data was collected using electrospray ion trap mass spectrometer.

### 4.3 Synthesis of the TMC-126 **(3)**

#### 4.3.1 (2S,3R)-3-Hydroxy-*N*-isobutyl-2-(*p*-methoxyphenoxy)-4-phenylbutanamide **(9)**

Into a flame-dried, nitrogen purged 1 L round-bottom flask equipped with a stir bar, was placed the glycolate aldol adduct **2** ^10^ (11.50 g, 24.80 mmol, 1.00 equiv) in dichloromethane (100 mL). Imidazole (5.06 g, 74.4 mmol, 3.00 equiv) and isobutylamine (5.0 mL, 50 mmol, 2.00 equiv) were added to the reaction mixture and the solution was stirred overnight at ambient temperature. The reaction mixture was diluted with dichloromethane (100 mL), transferred to a separatory funnel, and sequentially washed with aqueous 1 M HCl (2 × 30 mL), aqueous 2 M NaOH (2 × 30 mL), and brine (30 mL). The organic layer was dried (MgSO_4_) and concentrated under reduced pressure to afford β-hydroxyamide **9** (8.77 g, ∼99% yield) as white crystals that were characterized without further purification. Mp: 84.0–86.0 °C. [α]_D_ = +42.7 (*c* = 0.348, CHCl_3_). ^1^H NMR (500 MHz, CDCl_3_): δ 6.88 (d, *J* = 9.3 Hz, 2H), 6.83 (d, *J* = 9.3 Hz, 2H), 6.49 (broadened triplet, 1H), 4.56 (d, *J* = 2.6 Hz, 1H), 4.30 (broad singlet, 1H), 3.77 (s, 3H), 3.73 (dd, *J* = 7.8, 2.6 Hz, 1H), 3.12 (t, *J* = 6.6 Hz, 2H), 2.01–1.92 (m, 1H), 1.78–1.70 (m, 1H), 1.06 (d, *J* = 6.7 Hz, 3H), 0.89 (d, *J* = 6.7 Hz, 3H), 0.84 (d, *J* = 3.7 Hz, 3H), 0.83 (d, *J* = 3.7 Hz, 6H) ppm. ^13^C­{^1^H} NMR (100 MHz, CDCl_3_): δ 170.7, 154.9, 151.6, 116.2, 114.9, 80.6, 55.7, 46.4, 31.0, 28.5, 19.94, 19.90, 19.3, 18.7 ppm. IR (CDCl_3_): 3470, 3338, 1634, 817 cm^–1^. HRMS (ESI-TOF) *m*/*z*: [M + Na]^+^ calcd for C_21_H_27_NNaO_4_, 380.1832; found, 380.1832.

#### 4.3.2 (2R,3S)-2-Hydroxy-4-(isobutylamino)-3-(*p*-methoxyphenoxy)-1-phenylbutane **(8)**

Into a flame-dried, nitrogen purged 1 L round-bottom flask fitted with a pressure equalizing addition funnel was added β-hydroxyamide **9** (9.24 g, 25.8 mmol, 1.00 equiv) and THF (105 mL). To this reaction mixture was added sodium borohydride (2.35 g, 62.0 mmol, 2.40 equiv) in portions while the reaction mixture was slowly stirred. Once the addition of the sodium borohydride was complete, iodine (I_2_) (7.22 g, 28.4 mmol, 1.10 equiv) dissolved in 20 mL THF was then added dropwise via the addition funnel to the reaction mixture. The reaction was then heated to reflux by the use of a heating mantle and stirred overnight. The reaction was cooled to room temperature and then with an ice bath. The reaction was then quenched by the slow addition of methanol (100 mL) and stirred for 30 min. The mixture was concentrated under reduced pressure (rotary evaporation) to remove the THF solvent, and the reaction mixture was extracted with ethyl acetate (100 mL). The organic layer was washed with aqueous 1 M NaOH (2 × 30 mL) and with brine (30 mL), dried over MgSO_4,_ and concentrated again under reduced pressure to afford the title compound as a viscous oil (7.99 g, 90% yield). The quality of the ^1^H NMR spectrum suggested that the material could move on in the synthesis without further treatment. [α]_D_ = −54.3 (*c* = 0.591, CHCl_3_). ^1^H NMR (400 MHz, CDCl_3_): δ 7.25–7.15 (m, 5H), 6.89–6.82 (m, 4H), 4.19 (td, *J* = 7.1, 2.01 Hz, 1H), 4.08–4.06 (m, 1H), 3.78 (s, 3H), 3.27 (dd, *J* = 12.7, 4.3 Hz, 1H), 2.98 (d, *J* = 7.3 Hz, 2H), 2.72 (dd, *J* = 12.7, 2.3 Hz, 1H), 2.42 (dd, *J* = 11.5, 6.4 Hz, 1H), 2.32 (dd, *J* = 11.5, 7.1 Hz, 1H), 1.74–1.64 (m, 1H), 0.89 (d, *J* = 1.7, 3H), 0.87 (*J* = 1.7 Hz, 3H) ppm. ^13^C­{^1^H} NMR (100 MHz, CDCl_3_): δ 153.4, 150.5, 137.5, 128.5, 127.4, 125.2, 116.2, 113.8, 75.7, 74.5, 57.0, 54.7, 49.6, 38.7, 27.1, 19.5 ppm. IR (CDCl_3_):153.4, 150.5, 137.5, 128.5, 127.4, 125.2, 116.2, 113.8, 75.7, 74.5, 56.9 54.7, 49.6, 38.7, 27.1, 19.5 ppm. IR (CDCl_3_): 3316, 3027, 2060, 1948, 1508, 1464, 751 cm^–1^. HRMS (ESI-TOF) *m*/*z*: [M + H]^+^ calcd for C_21_H_31_NO_3_, 344.2220; found, 344.2220.

#### 4.3.3 (2R,3S)-3-(*p*-Methoxyphenoxy)-4-(*N*-isobutyl-*N*-p-methoxyphenylsulfonamido)-1-phenyl-2-(*p*-methoxyphenylsulfonato)­butane **(10)**

To a flame-dried, nitrogen purged 500 mL round-bottom flask were added the γ-aminoalcohol **9** (6.96 g, 20.3 mmol, 1.00 equiv) and dichloromethane (60 mL). To this mixture was added two equivalents of *p*-methoxybenzenesulfonyl chloride (8.79 g, 42.6 mmol, 2.10 equiv), DMAP (5.20 g, 42.6 mmol, 2.10 equiv), and triethylamine (8.5 mL, 60 mmol, 2.96 equiv). The reaction mixture stirred overnight and was diluted with dichloromethane (100 mL), washed with aqueous 1 M hydrochloric acid (2 × 50 mL) and brine (50 mL). The organic layer was dried over MgSO_4_, gravity filtered, and the solvent was removed under reduced pressure to yield a yellow viscous oil. The crude residue was purified by column chromatography on silica gel (hexane/ethyl acetate, 9:1). The material obtained was then crystallized from diethyl ether and hexanes to afford the title compound as white crystals (7.07 g, 51% yield). Mp: 110–112 °C. [α]_D_ = +82.9 (*c* = 1.86, CHCl_3_). ^1^H NMR (400 MHz, CDCl_3_): δ 7.73 (d, *J* = 8.9 Hz, 2H), 7.50 (d, *J* = 8.9 Hz, 2H), 7.11–7.05 (m, 3H), 6.90 (d, *J* = 8.9 Hz, 2H), 6.83 (dd, *J* = 7.5 Hz, 1.2 Hz, 2H), 6.79–6.74 (m, 4H), 6.72 (d, *J* = 9.0 Hz, 2H), 4.81–4.80 (m, 1H), 4.71–4.67 (m, 1H), 3.84 (s, 3H), 3.83 (s, 3H), 3.79 (s, 3H), 3.66 (dd, *J* = 15.3 Hz, 1.7 Hz, 1H), 3.39 (dd, *J* = 15.3 Hz, 9.2 Hz), 3.08–3.01 (m, 2H), 2.87 (dd, *J* = 13.7 Hz, 6.4 Hz), 2.71­(dd, *J* = 14.3 Hz, 9.1 Hz), 2.04–1.93 (m, 1H), 0.84 (d, *J* = 6.9 Hz, 3H), 0.82 (d, *J* = 6.9 Hz, 3H) ppm.^13^C NMR (100 MHz, CDCl_3_): 163.5, 162.7, 154.5, 151.1, 136.0, 131.4, 129.9, 129.4, 129.2, 128.4, 127.1, 126.6, 116.8, 114.7, 114.2, 114.1, 81.0, 76.3, 56.8, 55.7, 55.6, 55.5, 48.1, 35.0, 26.5, 20.0, 19.9 ppm. IR (CHCl_3_): 1506, 1262, 1168 cm^–1^. HRMS (ESI-TOF) *m*/*z*: [M + Na]^+^ calcd for C_35_H_41_NNaO_9_S_2_ 706.2115; found, 706.2103.

#### 4.3.4 (2R,3S)-3-Azido-2-(*p*-methoxyphenoxy)-1-[(*N*-isobutyl-*N*-p-methoxyphenyl)­sulfonamido]-4-phenylbutane **(7)**

To a flame-dried, nitrogen purged 250 mL round-bottom flask was added the bis­(sulfonyl) adduct **10** (6.80 g, 9.94 mmol, 1.00 equiv), dimethylsulfoxide (35 mL), and treated with sodium azide (1.95 g, 29.8 mmol, 3.00 equiv). The reaction temperature was then adjusted to 60 °C and the reaction stirred overnight. The reaction was diluted with diethyl ether (200 mL) and 1 M aqueous NaOH (50 mL), and the layers were separated. The organic layer was then washed with brine (2 × 25 mL), dried with MgSO_4_, and the solvents were removed under reduced pressure to give a crude oil. This material was purified by flash column chromatography on silica gel with ethyl acetate and hexanes (9:1) to afford the azide **7** as a light-yellow viscous oil (5.36 g, ∼99%). [α]_D_ = +57.9 (*c* = 0.66, CHCl_3_). ^1^H NMR (400 MHz, CDCl_3_): δ 7.73 (m, 2H), 7.39–7.28 (m, 5H), 6.94 (m, 2H), 6.77 (m, 2H), 4.64 (dt, *J* = 8.4, 2.7, 1H), 3.97–3.92 (m, 1H), 3.88 (s, 3H), 3.78 (s, 3H), 3.73 (dd, *J* = 12.8, 2.7 Hz, 1H), 3.22 (dd, *J* = 15.4, 8.5 Hz, 1H), 3.15 (dd, *J* = 13.5, 8.5 Hz, 1H), 2.95 (dd, *J* = 14.1, 5.61 Hz, 1H), 2.85–2.79 (m, 2H), 2.07–1.99 (m, 2H), 0.87 (d, *J* = 6.6 Hz, 3H), 0.84 (d, *J* = 6.6 Hz, 3H) ppm. ^13^C NMR (100 MHz): δ 162.8, 154.6, 150.7, 137.2, 131.0, 129.4, 129.3, 128.8, 127.0, 117.2, 114.8, 114.2 80.1, 64.4, 58.1, 55.7, 55.6, 48.8, 37.0, 26.9, 20.1, 19.9 ppm. IR (CHCl_3_): 2961, 2119, 1597, 1507, 1340 cm^–1^. HRMS (ESI-TOF) *m*/*z*: [M + Na]^+^ calcd for C_28_H_34_N_4_O_5_SNa 561.2148; found, 561.2127.

#### 4.3.5 (2R,3S)-3-Azido-2-hydroxy-1-[(*N*-isobutyl-*N*-*p*-methoxyphenyl)-sulfonamido]-4-phenyl butane **(6)**

To a 250 mL round-bottom flask equipped with a stir bar was added the azide substrate **7** (4.25 g, 7.89 mmol, 1.00 equiv), acetonitrile (77 mL), and deionized water (26.0 mL). Ceric ammonium nitrate (17.30 g, 31.55 mmol, 4.00 equiv) was then added, and the reaction mixture was stirred for 60 min. The reaction was diluted with diethyl ether (200 mL) and the reaction mixture was washed with brine (2 × 50 mL). The organic layer was dried (MgSO_4_), gravity filtered, and the solvent was then concentrated under reduced pressure to afford a yellow crude residue. The crude product was then purified by column chromatography (ethyl acetate/hexanes, 9:1) to give the β-azido-α-hydroxyamide **6** as a yellow oil (1.74 g, 51%). [α]_D_ = +59.3 (*c* = 0.18, CHCl_3_). ^1^H NMR (400 MHz, CDCl_3_): δ 7.77 (d, *J* = 8.9 Hz, 2H), 7.37–7.28 (m, 5H), 7.03 (d, *J* = 8.9 Hz, 2H), 3.90 (s, 3H), 3.82–3.77 (m, 1H), 3.65–3.60 (m, 1H), 3.28 (dd, *J* = 15.3 Hz, 9.3 Hz), 3.15–3.03 (m, 3H), 2.85–2.79 (m, 2H), 1.90–1.79 (m, 2H), 0.96 (d, *J* = 6.6 Hz, 3H), 0.90 (d, *J* = 6.6 Hz, 3H) ppm. ^13^C NMR (100 MHz, CDCl_3_): 163.2, 137.2, 129.8, 129.5, 129.4, 128.7, 126.9, 114.4, 71.8, 66.6, 58.9, 55.6, 52.9, 37.0, 27.2, 20.2, 19.8 ppm. IR (CHCl_3_): 3489, 2963, 2106 cm^–1^. HRMS (ESI-TOF) *m*/*z*: [M + Na]^+^ calcd for C_21_H_28_N_4_NaO_4_S 455.1723; found, 455.1715.

#### 4.3.6 (3R,3aS,6aR-Hexahydrofuro­[2,3-*b*]­furan-3-yl-(2S,3R)-4-[(*N*-isobutyl-*N*-p-methoxy phenyl)­sulfonamido]-3-hydroxy-1-phenylbutyl)­carbamate (TMC-126) **(3)**

In a flame-dried, nitrogen purged 250 mL round-bottom flask was placed the β-azidoamide **6** (1.60 g, 3.70 mmol, 1.00 equiv) and THF (44 mL). The commercially available carbonate **11** (1.06 g, 3.89 mmol, 1.05 equiv) was added to the reaction mixture followed by the addition of 10% palladium on carbon (0.32 g). The system was purged with nitrogen gas for 10 min and a hydrogen filled balloon was connected to the reaction flask and the reaction stirred overnight at ambient temperatures. The reaction mixture was then diluted with chloroform and filtered through a 1:1 mixture of Celite and MgSO_4_. The residue was concentrated under reduced pressure to give a yellow reside that was subjected to silica gel column chromatography with 2% methanol in dichloromethane to afford TMC-126 **(3)** as a viscous oil (1.18 g, 56%). [α]_D_ = +11.8 (*c* = 0.396, CHCl_3_). ^1^H NMR (400 MHz, CDCl_3_): δ 7.71 (d, *J* = 8.9 Hz, 2H), 7.30–7.21­(m, 5H), 6.99 (d, *J* = 8.9 Hz, 2H), 5.64 (d, *J* = 5.2 Hz, 1H), 5.02 (q, 1H), 4.92 (d, *J* = 8.3 Hz, 1H), 3.97–3.93 (m, 1H), 3.88 (s, 3H), 3.85–3.82 (m, 1H), 3.72–3.66 (m, 2H), 3.20–2.78 (m, 6H), 1.83 (p, 1H), 1.69–1.51 (m, 3H), 0.93 (d, *J* = 6.6 Hz, 3H), 0.88 (d, *J* = 6.6 Hz, 3H) ppm. ^13^C NMR (100 MHz, CDCl_3_): 163.3, 155.5, 137.6, 129.8, 129.5, 129.4, 128.6, 126.6, 114.4, 109.3, 73.5, 72.8, 70.8, 69.6, 58.8, 55.7, 55.2, 53.8, 45.4, 35.7, 27.3, 25.8, 20.2, 19.9 ppm. IR (CHCl_3_): 3482, 3355, 1718 cm^–1^. HRMS (ESI-TOF) *m*/*z*: [M + Na]^+^ calcd for C_28_H_38_N_2_O_8_SNa 585.2241; found, 585.2230.

### 4.4 Synthesis of the PMX Inhibitor **49c (4)**

#### 4.4.1 (2S,3R)-3-Hydroxy-2-(*p*-methoxyphenoxy)-*N*-(R)-(1-phenethyl)-4-phenylbutanamide **(16)**

Into a flame-dried, nitrogen purged 1 L round-bottom flask equipped with a stir bar, was placed the glycolate aldol adduct **(2)** ^10^ (6.67 g, 14.4 mmol, 1.00 equiv) and dichloromethane (80 mL). To this mixture was added imidazole (1.96 g, 28.8 mmol, 2.0 equiv) and (*R*)-α-methylbenzylamine (2.80 mL, 21.6 mmol, 1.5 equiv) and the reaction was stirred overnight at ambient temperatures. The reaction was diluted with dichloromethane (100 mL), transferred into a separatory funnel, and sequentially washed with aqueous 1 M HCl (2 × 50 mL), aqueous 2 M NaOH (2 × 50 mL), and brine (50 mL). The organic layer was dried (MgSO_4_), gravity filtered, and the solvent was concentrated under reduced pressure to afford compound **16** as a viscous oil. The product was purified via crystallization with ethyl acetate and hexanes to afford pure white crystals (4.95 g, 85% yield). This reaction was conducted multiple times to accumulate material for the next reaction. Mp: 141–142 °C. [α]_D_ = −35.9 (*c* = 1.10, CHCl_3_). ^1^H NMR (400 MHz, CDCl_3_): δ 7.32 (d, *J* = 4.4 Hz, 4H), 7.25–7.17 (m, 4H), 7.13–7.11 (m, 2H), 6.91–6.84 (m, 4H), 6.80 (d, *J* = 8.4 Hz, 1H), 5.18 (p, 1H), 4.51 (d, *J* = 2.9 Hz, 1H), 4.29–4.25 (m, 1H), 3.79 (s, 3H), 2.93 (dd, *J* = 13.8, 5.1 Hz, 1H), 2.85 (dd, *J* = 13.8, 8.7 Hz, 1H), 2.59 (broad singlet, 1H), 1.43 (d, *J* = 6.9 Hz, 3H) ppm. ^13^C­{^1^H} NMR (100 MHz, CDCl_3_): δ 169.9, 155.1, 151.3, 142.7, 137.7, 129.4, 128.7, 128.5, 127.4, 126.6, 126.0, 116.8, 114.9, 80.9, 73.1, 55.7, 48.5, 39.5, 21.7 ppm. IR (CHCl_3_): 3406, 3267, 1649, 1207 cm^–1^. HRMS (ESI-TOF) *m*/*z*: [M + Na]^+^ calcd for C_2525_H_27_NNaO_4_; 428.1832; found, 428.1832.

#### 4.4.2 (2S,3R)-2-(*p*-Methoxyphenoxy)­3-(*p*-nitrophenylsulfonato)-*N*-(R)-(1-phenethyl)-4-phenyl Butanamide **(17)**

A flame-dried, nitrogen purged 500 mL round-bottom flask was charged with β-hydroxyamide **16** (6.02 g, 14.8 mmol, 1.00 equiv) and dichloromethane (75 mL) followed by the addition of *p*-nitrobenzenesulfonyl chloride (3.60 g, 16.3 mmol, 1.10 equiv) and DMAP (0.452 g, 3.70 mmol), and triethylamine (2.30 mL, 17.0 mmol, 1.15 equiv). The reaction was stirred overnight and was diluted with dichloromethane (60 mL) and washed with aqueous 1 M HCl (2 × 50 mL) and brine (50 mL). The organic layer was dried (MgSO_4_), gravity filtered, and the solvents were removed under vacuum to afford a yellow viscous oil. The product started to crystallize and was purified via recrystallization with ethyl acetate and hexanes to afford the title compound **(17)** as a white crystalline product (7.70 g, 88%). Mp: 136.1–137 °C. [α]_D_ = −35.0 (*c* = 1.10, CHCl_3_). ^1^H NMR (400 MHz, CDCl_3_): δ 8.01 (d, *J* = 9.0 Hz, 2H), 7.71 (d, *J* = 9.0 Hz, 2H), 7.34–7.27 (m, 3H), 7.17–7.11 (m, 5H), 6.94 (d, *J* = 7.2 Hz, 2H), 6.87–6.81 (m, 4H), 6.66 (d, *J* = 8.7 Hz, 1H), 5.27 (ddd, *J* = 8.7, 6.4, 3.1 Hz, 1H), 4.91 (p, 1H), 4.41 (d, *J* = 3.1 Hz, 1H), 3.79 (s, 3H), 3.30 (dd, *J* = 13.7, 6.5 Hz, 1H), 3.24 (dd, *J* = 13.7, 8.7 Hz, 1H), 1.28 (d, *J* = 6.8 Hz, 3H) ppm. ^13^C­{^1^H} NMR (100 MHz, CDCl_3_): δ 166.9, 155.3, 151.1, 150.4, 142.3, 141.8, 134.8, 129.5, 129.1, 128.9, 128.8, 127.6, 127. 3, 125.7, 124.0, 116.3, 115.1, 84.2, 78.5, 55.7, 48.7, 37.5, 22.7 ppm. IR: HRMS (ESI-TOF) *m*/*z*: [M + Na]^+^ calcd for C_31_H_30_N_2_NaO_8_S 613.1615; found, 613.1593.

#### 4.4.3 (2R,3S)-3-Azido-2-(*p*-methoxyphenoxy)-*N*-(R)-(1-phenethyl)-4-phenylbutanamide **(15)**

In a flame-dried, nitrogen purged 500 mL round-bottom flask was combined **17** (9.00 g, 15.5 mmol, 1.00 equiv), DMSO (62 mL), and sodium azide (4.03 g, 62.0 mmol, 4.00 equiv). The reaction was heated to 60 °C and stirred overnight. The reaction was cooled to room temperature and diluted with diethyl ether (120 mL), transferred into a separatory funnel, and washed twice with aqueous 1 M NaOH (2 × 40 mL) and brine (2 × 40 mL). The organic layer was separated, dried (MgSO_4_), and filtered. The solvent was then removed under reduced pressure to afford a solid product. The product **(15)** was recrystallized from ethyl acetate and hexanes and was recovered as a white powder (4.68 g, 71%). Mp: 119–121 °C. [α]_D_ = −92.7 (*c* = 1.10, CHCl_3_). ^1^H NMR (400 MHz, CDCl_3_): δ 7.35 (d, *J* = 4.3 Hz, 4H), 7.33–7.22 (m, 6H), 6.87–6.82 (m, 4H), 6.78 (d, *J* = 8.2 Hz, 1H), 5.23–5.16 (m, 1H), 4.68 (d, *J* = 2.8 Hz, 1H), 4.04–3.99 (m, 1H), 3.78 (s, 3H), 3.01–2.90 (m, 2H), 1.46 (d, *J* = 6.9 Hz, 3H) ppm. ^13^C­{^1^H} NMR (100 MHz, CDCl_3_): δ 167.4, 155.3, 150.9, 142.5, 137.1, 129.3, 128.9, 128.7, 127.6, 126.9, 126.1, 116.9, 114.9, 81.7, 64.7, 55.7, 48.7, 35.4, 21.6 ppm. IR: 3334, 2104, 1645, 700 cm^–1^ HRMS (ESI-TOF) *m*/*z*: [M + Na]^+^ calcd for C_25_H_26_N_4_NaO_3_: 453.1897 found, 453.1890.

#### 4.4.4 (2R,3S)-3-Azido-2-hydroxy-*N*-(R)-(1-phenethyl)-4-phenylbutanamide **(19)**

β-Azidoamide **15** (2.21 g, 5.14 mmol, 1.00 equiv) was dissolved in acetonitrile (65 mL) and sulfuric acid (1M, 15.4 mL, 15.4 mmol, 3.00 equiv). The resulting mixture was stirred for 20 min to allow for reaction homogeneity. Ceric ammonium nitrate (8.45 g, 15.4 mmol, 3.00 equiv) was then added the reaction mixture was stirred for 100 min. The reaction was diluted with dichloromethane (100 mL) and subsequently treated with an aqueous solution of 1 M NaOH (2 × 100 mL) and brine (100 mL). The organic layer was separated and dried (MgSO_4_), filtered, and the solvent was removed under reduced pressure to afford a reddish oil that was purified via flash column chromatography (silica gel) with dichloromethane/methanol (9:1) to yield a reddish-brown oil (1.61 g, 97%). [α]_D_ = +60.2 (*c* = 1.10, CHCl_3_). ^1^H NMR (400 MHz, CDCl_3_): δ 7.34–7.21 (m, 10H), 6.79 (d, *J* = 7.9 Hz, 1H), 5.16 (m, 1H), 4.20 (d, *J* = 4.7 Hz, 1H), 3.95 (p, *J* = 4.7 Hz, 1H), 3.00 (broad singlet, OH), 2.89 (d, *J* = 14.2, 4.1 Hz, 1H), 2.82 (d, *J* = 14.2, 9.4 Hz), 1.54 (d, *J* = 6.9 Hz) ppm. ^13^C­{^1^H} NMR (100 MHz, CDCl_3_): δ 169.8, 142.6, 137.3 129.4, 128.8, 128.6, 127.6, 126.9, 126.1, 73.5, 66.3, 48.9, 35.0, 21.8 ppm. IR: 3559, 3361, 2105, 1661 cm^–1^. HRMS (ESI-TOF) *m*/*z*: [M + Na]^+^ calcd for C_18_H_20_N_4_NaO_2_; 347.1478; found, 347.1475.

#### 4.4.5 (2R,3S)-3-Amino-2-hydroxy-*N*-(R)-[(1-phenethylamino)-4-phenylbutane **(14)**

The β-azido-α-hydroxyamide **19** (1.29 g, 4.00 mmol, 1.00 equiv) and THF (20 mL) were combined in a flame-dried, nitrogen purged 500 mL round-bottom flask. To this solution was added lithium aluminum hydride (0.911 g, 24.0 mmol, 6.00 equiv). The reaction was heated to reflux and stirred for 30 h. The reaction was then cooled to room temperature and then to 0 °C (ice bath) and was quenched by treating the mixture with an aqueous solution of 1 M NaOH (50 mL). The quenched reaction was then diluted with dichloromethane (80 mL) and a precipitate separated out. The precipitate was removed via vacuum filtration and the filtrate transferred into a separatory funnel. The combined organic layers were washed with an aqueous solution of 1 M NaOH (80 mL) and brine (80 mL). The collected organic layer was filtered, and the solvent was removed under reduced pressure. This process afforded a crude product that was purified by flash column chromatography (dichloromethane: methanol, 7:3). The title compound was recovered as a clear green viscous oil (0.763 g, 67%). [α]_D_ = +24.2 (*c* = 1.10, CHCl_3_). ^1^H NMR (400 MHz, CDCl_3_): δ 7.35–7.13 (m, 10H), 3.76 (q, *J* = 6.7 Hz, 1H), 3.59–3.54 (m, 1H), 3.08–3.03 (m, IH), 2.82 (dd, *J* = 13.6, 4.0 Hz, 1H), 2.73 (dd, *J* = 12.0, 3.37 Hz, 1H), 2.54 (dd, *J* = 12.0, 8.28 Hz, 1H), 2.40 (dd, *J* = 13.6, 9.8 Hz, 1H), 2.00 (broad singlet, 4H), 1.38 (d, *J* = 6.7 Hz, 3H) ppm. ^13^C­{^1^H} NMR (100 MHz, CDCl_3_): δ 145.2, 139.2, 129.2, 128.6, 128.5, 127.1, 126.6, 126.3, 72.2, 58.1, 55.9, 48.8, 39.7, 24.4 ppm. IR: 3352, 3295, 3026, 1602 cm^–1^. HRMS (ESI-TOF) *m*/*z*: [M + H]^+^ calcd for C_18_H_25_N_2_O; 285.1961; found, 285.1958.

#### 4.4.6 *N*-(*p*-Carboxymethylbenzoyl)-*N*-methylpiperidine **(21)**

Mono methyl terephthalate (9.00 g, 50.0 mmol, 1.00 equiv) and THF (200 mL) were combined in flame-dried, nitrogen purged 1 L round-bottom flask. Thionyl chloride (5.47 mL, 75.0 mmol, 1.50 equiv) was added via syringe and the reaction stirred for 45 min as determined by monitoring the reaction by TLC. At this stage, 1-methylpiperazine (8.31 mL, 75.0 mmol, 1.50 equiv) was added to the reaction mixture. The reaction was stirred for another 90 min and was quenched by the addition of a saturated aqueous solution of sodium bicarbonate. The product was extracted with dichloromethane (50 mL) and sequentially washed with an aqueous solution of 1 M NaOH and brine (50 mL). The organic layer was dried over MgSO_4_, filtered and excess solvent removed under reduced pressure to afford a brown colored viscous oil (8.45 g, 65%). Initial NMR analysis showed the formation of the target compound. Mp: 66.7–68.0 °C. ^1^H NMR (400 MHz, CDCl_3_): δ 8.08 (d, *J* = 8.5 Hz, 2H), 7.47 (d, *J* = 8.5 Hz, 2H), 3.94 (s, 3H), 3.81 (broad singlet, 2H), 3.39 (broad singlet, 2H), 2.49 (broad singlet, 2H), 2.35 (broad singlet, 2H), 2.32 (s, 3H) ppm. ^13^C­{^1^H} NMR (100 MHz, CDCl_3_): δ 169.2, 166.3, 140.1, 131.2, 129.8, 127.0, 55.2, 54.7, 52.3, 47.5, 46.0, 42.0 ppm. IR: 2935, 1723, 1615, 862 cm^–1^. HRMS (ESI-TOF) *m*/*z*: [M + H]^+^ calcd for C_14_H_19_N_2_O_3_: 263.1390; found, 263.1386.

#### 4.4.7 4-(*N*-Methylpiperazinylcarbonyl) Benzoic Acid **(13)**

The 1-methylpiperazine carbonyl benzoate ester (8.45 g, 32.1 mmol, 1.00 equiv) was added to a 500 mL flame-dried round-bottom flask and dissolved in dichloromethane (43 mL). A methanolic solution of 2 M NaOH was prepared by dissolving 1.22 g of NaOH in methanol (15 mL) with gentle heating. The methanolic solution (4.5 mL, 64.20 mmol, 2.00 equiv) was added dropwise in a 9:1 ratio of CH_2_Cl_2_/CH_3_OH and stirred at room temperature for 3 h. The reaction mixture was removed via rotary evaporation and the residue diluted with 100 mL of deionized water. The unreacted ester and byproducts were removed via extraction with ethyl acetate and the aqueous layer containing 1-methylpiperazine carbonyl sodium benzoate was cooled to ice temperature and acidified with concentrated hydrochloric acid. Water was then removed under reduced pressure to give a white semi solid product. Crystallization with reaction grade acetone and vacuum filtration afforded 1-methylpiperazine carbonyl benzoic acid (5.38 g, 67%) as white crystals. Mp: 244–246 °C. ^1^H NMR (400 MHz, D_2_O): δ 8.10 (d, *J* = 8.5 Hz, 2H), 7.56 (d, *J* = 8.5 Hz, 2H), 3.93–3.89 (m, 1H), 3.68 (d, *J* = 12.4 Hz, 1H), 3.60–3.46 (m, 3H), 3.40–3.32 (m, 1H), 3.26–3.13 (m, 2H), 2.95 (s, 3H) ppm. ^13^C­{^1^H} NMR (100 MHz, D_2_O): δ 171.6, 169.7, 137.8, 131.9, 130.2, 127.1, 52.9, 50.0, 44.5, 43.0, 40.8, 39.4 ppm. IR: 3321, 2950, 1709, 1638, 1608 cm^–1^. HRMS (ESI-TOF) *m*/*z*: [M + H]^+^ calcd for C_13_H_17_N_2_O_3_: 249.1234; found, 249.1230.

#### 4.4.8 (2R,3S)-3-Hydroxy-2-(*N*′-methylpiperazinecarbonylbenzamido)-*N*-(*R*)-(1-phenethyl Amino)-4-phenylbutane **(4)**

In a flame-dried, nitrogen purged round-bottom flask was placed the γ-diamine **14** (0.762 g, 2.68 mmol, 1.0 equiv) dissolved in DMSO (45 mL). To this mixture was added *N*-methylpiperazine carbonyl benzoic acid **13** (0.670 g, 2.68 mmol, 1.0 equiv) was added and stirring continued to completely dissolve the materials. The coupling agent HATU (1.32 g, 3.48 mmol, 1.30 equiv) was then added to the reaction mixture followed by the addition of 4-ethylmorpholine (1.22 mL, 9.65 mmol, 3.60 equiv). At this stage, the flask was wrapped with aluminum foil to prevent exposure to light and photochemical decomposition of the HATU reagent. The reaction stirred overnight and was quenched by the addition of a saturated aqueous solution of sodium bicarbonate. The reaction mixture was transferred into a separatory funnel and diluted with ethyl acetate (3 × 50 mL) and washed with deionized water (200 mL) and brine (70 mL). The organic layer was separated, dried (MgSO_4_), and the solvent was removed under reduced pressure to afford a dark brown crude. This material was purified via flash chromatography (silica gel) using dichloromethane: methanol (9:1). The compound was recovered as a lightly colored viscous oil (0.620 g, 45%). [α]_D_ = −20.1 (*c* = 1.10, CHCl_3_). ^1^H NMR (400 MHz, CDCl_3_): δ 7.67 (d, *J* = 8.4 Hz, 2H), 7.38 (d, *J* = 8.4 Hz, 2H), 7.32–7.27 (m, 5H), 7.25–7.16 (m, 5H), 4.45–4.38 (m, 1H), 3.79–3.74 (m, 3H), 3.67–3.63 (m, 1H), 3.37 (broad singlet, 2H), 2.99–2.94 (m, 1H), 2.84 (dd, *J* = 14.1, 6.1 Hz, 1H), 2.71–2.69 (m, 1H), 2.60 (dd, *J* = 12.6, 4.8 Hz, 1H), 2.48 (broad singlet, 2H), 2.32 (s, 5H), 1.44 (dd, *J* = 6.7 Hz, 3H) ppm. ^13^C­{^1^H} NMR (100 MHz, CDCl_3_): δ 169.4, 166.8, 144.0, 138.5, 137.9, 135.6, 129.2, 128.7, 128.5, 127.4, 127.3, 127.0, 126.8, 126.5, 69.9, 58.8, 54.9, 49.1, 47.5, 45.9, 42.1, 36.9, 23.9 ppm. IR: 3646, 3300, 1624 cm^–1^. HRMS (ESI-TOF) *m*/*z*: [M + H]^+^ calcd for C_31_H_39_N_4_O_3_: 515.3017; found, 515.3010.

### 4.5 Synthesis of Rathi’s Covid-19 Inhibitor **(5)**

#### 4.5.1 *N*-(*p*-Fluorophenyl)-3-hydroxy-2-(*p*-methoxyphenoxy)-4-phenylbutanamide **(24)**

To a flame-dried, nitrogen purged 1 L round-bottom flask equipped with a stir bar was added the aldol adduct **2** ^10^ (13.5 g, 29.1 mmol, 1.00 equiv), dichloromethane (100 mL), *p*-fluoroaniline (8.3 mL, 87.3 mmol, 3.00 equiv), and imidazole (2.18 g, 32.0 mmol, 1.10 equiv). The reaction was stirred overnight and allowed to vent via tubing as some gaseous byproducts can form. Upon completion of the overnight stirring, the mixture was diluted with dichloromethane (100 mL) and two washes of 1 M HCl (2 × 40 mL). The organic layer was then treated with two washes of 1 M NaOH (2 × 40 mL) and a final wash of brine solution (40 mL). The organic layer was then dried over MgSO_4_, and the solvent was removed by rotary evaporation. The material was purified via recrystallization with ethyl acetate and hexanes, producing compound **24** (8.67 g, 75% yield) as a white powder. Melting point: 138.4–140.1 °C. [α]_D_ = −29.54 (*c* = 1.00, CHCl_3_). ^1^H NMR (500 MHz, CDCl_3_): δ 8.22 (broad singlet, 1H), 7.46–7.49 (m, 2H), 7.26–7.28 (m, 1H), 7.16–7.23 (m, 4H), 6.99–7.03 (m, 2H), 6.93–6.95 (m, 2H), 6.85–6.88 (m, 2H), 4.56 (d, *J* = 2.8 Hz, 1H), 4.40 (td, *J* = 7.3, 2.8 Hz, 1H), 3.78 (s, 3H), 3.00 (d, *J* = 7.2 Hz). ^13^C­{^1^H} NMR (100 MHz, CDCl_3_): δ 168.9, 160.9, 158.4, 155.3, 151.3, 137.4, 132.9 (d, *J* = 3 Hz), 129.0 (d, *J* = 82 Hz), 126.7, 122.0 (d, *J* = 8 Hz), 115.9 (d, *J* = 167 Hz), 115.8, 115.5, 81.3, 73.3, 55.7, 39.8 *ppm*. IR: 3334, 1662, 1210, 822, 811, 701 cm^–1^. HRMS (ESI-TOF) *m*/*z*: [M + H]^+^ calcd for C_23_H_22_FNNaO_4_ 418.1425; found, 418.1418.

#### 4.5.2 *N*-(*p*-Fluorophenyl)-2-(*p*-methoxyphenoxy)-3-(*p*-nitrobenzenesulfonyl)-4-phenyl Butanamide **(27)**

To a flame-dried, nitrogen purged 1 L round-bottom flask equipped with a stir bar was added transamidation product **24** (7.70 g, 19.5 mmol, 1.00 equiv), dichloromethane (100 mL), *p*-nitrobenzenesulfonyl chloride (4.75 g, 21.5 mmol, 1.10 equiv), DMAP (0.600 g, 4.88 mmol), and triethylamine (3.13 mL, 22.4 mmol, 1.15 equiv). The reaction was stirred overnight, and the reaction mixture was diluted with dichloromethane (100 mL) and sequentially treated with 1 M HCl (80 mL) and brine (80 mL). The organic layer was separated, dried (MgSO_4_), and the solvent was removed by rotary evaporation. The material was purified via column chromatography to produce compound **27** (9.28 g, 82% yield) as a yellow powder. Melting point: 145.0–147.0 °C. [α]_D_ = −54.65 (*c* = 1.00, CHCl_3_). ^1^H NMR (400 MHz, CDCl_3_): δ 7.94–7.98 (d, *J* = 9.0 Hz, 2H), 7.94 (broad singlet, 1H), 7.83–7.86 (d, *J* = 9.0 Hz, 2H), 7.17–7.21 (m, 5H), 6.99–7.02 (m, 2H), 6.90–6.94 (m, 2H), 6.83 (s, 4H), 5.33 (ddd, *J* = 8.1, 6.0, 2.1 Hz, 1H), 4.40 (d, *J* = 2.1 Hz, 1H), 3.77 (s, 3H), 3.40 (dd, *J* = 13.4, 6.0 Hz, 1H), 3.31 (dd, *J* = 13.4, 9.4 Hz, 1H). ^13^C­{^1^H} NMR (100 MHz, CDCl_3_): δ 166.1, 160.8, 158.4, 155.5, 150.9, 150.3, 141.7, 134.4, 132.4 (d, *J* = 3 Hz), 129.6, 128.9 (d, *J* = 78 Hz), 127.5, 124.1, 121.0 (d, *J* = 78 Hz), 115.8, 115.7 (d, *J* = 104 Hz), 115.5, 84.6, 78.3, 55.7, 38.1 *ppm*. IR: 3314, 1683, 1347, 1210, 838, 736, 706 cm^–1^. HRMS (ESI-TOF) *m*/*z*: [M + H]^+^ calcd for C_29_H_25_FN_2_NaO_8_S 603.1208; found, 603.1204.

#### 4.5.3 3-Azido-*N*-(*p*-fluorophenyl)-2-(*p*-methoxyphenoxy)-4-phenylbutanamide **(28)**

To a flame-dried, nitrogen purged 500 mL round-bottom flask equipped with a stir bar was added nosylation product **27** (7.29 g, 12.6 mmol, 1.00 equiv), DMSO (58 mL), and sodium azide (3.28 g, 50.4 mmol, 4.00 equiv). The reaction was heated to 40 °C for 48 h. Upon completion of the 48 h, the mixture was diluted with ethyl acetate (100 mL) and 1 M NaOH (75 mL). The organic layer was then treated with a final wash of brine solution (80 mL). The organic layer was then dried over MgSO_4_, and the solvent was removed by rotary evaporation. The material was purified via column chromatography to produce compound **27** (4.23 g, 10.4 mmol, 82% yield) as a white solid. Melting point: 94.5–96.2 °C. [α]_D_ = −58.43 (*c* = 0.93, CHCl_3_). ^1^H NMR (400 MHz, CDCl_3_): δ 8.23 (br s, 1H), 7.47–7.51 (m, 2H), 7.29–7.32 (m, 4H), 7.23–7.26 (m, 2H), 7.03 (t, *J* = 8.5 Hz, 2H), 6.88 (d, *J* = 9.3 Hz, 2H), 6.84 (d, *J* = 9.3 Hz, 2H), 4.74 (d, *J* = 2.7 Hz), 4.13–4.17 (m, 1H), 3.77 (s, 3H), 3.08–3.17 (m, 2H). ^13^C­{^1^H} NMR (100 MHz, CDCl_3_): δ 166.5, 161.0, 158.6, 155.5, 150.6, 136.7, 132.6 (d, *J* = 3.0 Hz), 129.0 (d, *J* = 67 Hz), 127.1, 122.0 (d, *J* = 8 Hz), 116.1 (d, *J* = 190 Hz), 115.9, 115.7, 81.5, 64.7, 55.7, 35.8 *ppm*. IR: 3316, 2108, 1664, 1210, 824, 736, 706 cm^–1^. HRMS (ESI-TOF) *m*/*z*: [M + H]^+^ calcd for C_23_H_21_FN_4_NaO_3_ 443.1490; found, 443.1483.

#### 4.5.4 3-Azido-*N*-(*p*-fluorophenyl)-2-hydroxy-4-phenylbutanamide **(23)**

To a 500 mL round-bottom flask equipped with a stir bar was added azide product **28** (5.02 g, 11.9 mmol, 1.00 equiv), acetonitrile (38 mL), 2 M H_2_SO_4_ (30 mL), and ceric ammonium nitrate (39.3 g, 71.6 mmol, 6.00 equiv). An extra amount of 2 M H_2_SO_4_ (∼3 mL) was added to increase the homogeneity of the solution. The reaction was stirred for 2 h and was then diluted with diethyl ether (50 mL) and washed with brine (2 × 25 mL), a saturated aqueous solution of NaHCO_3_ (25 mL), and brine (80 mL). The organic layer was then dried over MgSO_4_, and the solvent was removed by rotary evaporation. The material was purified via column chromatography to produce compound **23** (1.26 g, 34% yield) as a yellow, viscous oil. This compound was not fully characterized due to concerns of its stability as there was a color change in the material over time. Melting point: N/A (viscous oil). [α]_D_ = −39.9 (*c* = 0.46, CHCl_3_). ^1^H NMR (400 MHz, CDCl_3_): δ 8.35 (br s, 1H), 7.49–7.52 (m, 2H), 7.28–7.34 (m, 6H), 7.02–7.06 (m, 3H), 4.41–4.45 (m, 1H), 4.14–4.18 (m, 1H), 3.03 (dd, *J* = 14.0, 4.5 Hz, 1H), 2.92–2.98 (dd, *J* = 14.0, 9.3 Hz, 2H). IR: 3391, 2112, 1692, 1210, 829, 731, 702 cm^–1^. [M + H]^+^ calcd for C_16_H_15_FN_4_NaO_2_ 337.1071; found, 337.1073.

#### 4.5.5 3-Amino-1-(*N*-(*p*-fluorophenyl)-2-hydroxy)-4-phenyl-1-butanamine **(22)**

To a flame-dried, nitrogen purged 500 mL round-bottom flask fitted with a condenser and equipped with a stir bar was added butanamide **23** (1.00 g, 3.18 mmol, 1.00 equiv), THF (16 mL), and lithium aluminum hydride (0.727 g, 19.2 mmol, 6.03 equiv). The reaction was heated (70 °C) using and a heating mantle and stirred for 30 h. Upon completion of the allotted time, the reaction mixture was cooled in an ice bath and an aqueous solution of 1 M NaOH (10 mL) was added dropwise via the addition funnel to quench the unreacted lithium aluminum hydride. The solution was subsequently diluted with dichloromethane (20 mL) and vacuum filtered to remove the solid aluminum hydroxide waste byproduct. The collected organic layer was treated with an aqueous solution of 1 M NaOH (20 mL) and brine (20 mL). The organic layer was collected, dried (MgSO_4_), and the solvent was removed under reduced pressure. The material was purified via column chromatography (hexanes/ethyl acetate, 6:4) to produce the title compound **22** (0.465 g, 53% yield) as a pale brown solid. Melting point: 80.5–82.5 °C. [α]_D_ = 2.69 (*c* = 0.55, CHCl_3_). ^1^H NMR (400 MHz, CDCl_3_): δ 7.30–7.34 (m, 2H), 7.20–7.24 (m, 3H), 6.87–6.91 (m, 2H), 6.59–6.62 (m, 2H), 3.80–3.84 (m, 1H), 3.31 (dd, *J* = 12.0, 3.5 Hz, 1H), 3.22–3.26 (m, 1H), 3.17 (dd, *J* = 12.0, 8.3 Hz, 1H), 2.95 (dd, *J* = 13.0, 4.0 Hz, 1H), 2.56 (dd, *J* = 13.0, 10.0 Hz, 1H). ^13^C­{^1^H} NMR (100 MHz, CDCl_3_): δ 157.3, 155.0, 144.7 (d, *J* = 2 Hz), 138.7, 129.0 (d, *J* = 45 Hz), 126.6, 115.1 (d, *J* = 152 Hz), 115.0 (d, *J* = 122 Hz), 72.2, 55.8, 47.0, 39.2 ppm. IR: 3388, 3353, 814, 742, 701 cm^–1^. HRMS (ESI-TOF) *m*/*z*: [M + H]^+^ calcd for C_16_H_20_FN_2_O 275.1554; found, 275.1551.

#### 4.5.6 3-[(*tert*-Butoxycarbonyl)­amino]-1-(*N*-(*p*-fluorophenyl)-2-hydroxy)-4-phenyl-1-butanamine **(5)**

To a flame-dried, nitrogen purged 500 mL round-bottom flask equipped with a stir bar was added reduction product **22** (0.370 g, 1.35 mmol, 1.00 equiv), dichloromethane (9 mL), *tert*-butoxycarbonyl anhydride (0.308 g, 1.42 mmol, 1.05 equiv), and triethylamine (0.23 mL, 1.65 mmol, 1.22 equiv). The reaction was stirred overnight and subsequently diluted with dichloromethane (80 mL) and washed with an aqueous solution of 1 M NaOH (15 mL). The organic layer was then treated with a final wash of brine (15 mL). The organic layer was then dried over MgSO_4_, and the solvent was removed under reduced pressure. The material was purified via column chromatography with a solvent system of 90:10 hexanes/ethyl acetate to produce the title compound **5** (0.323 g, 64% yield) as a pale brown solid. Melting point: 131.3–133.3 °C. [α]_D_ = 15.04 (*c* = 0.87, CHCl_3_). ^1^H NMR (400 MHz, CDCl_3_): δ 7.30–7.33 (m, 2H), 7.22–7.24 (m, 3H), 6.87–6.92 (m, 2H), 6.63–6.66 (m, 2H), 4.57 (d, *J* = 7.7 Hz, 1H), 3.87–3.94 (m, 1H), 3.73–3.78 (m, 1H), 3.30 (dd, *J* = 13.0, 3.5 Hz, 1H), 3.11–3.16 (m, 3H), 2.88 (dd, *J* = 14.2, 5.0 Hz, 1H), 1.37 (s, 10H). ^13^C­{^1^H} NMR (125 MHz, CDCl_3_): δ 157.1, 156.4, 155.3, 144.8, 137.7, 129.0 (d, *J* = 101 Hz), 126.6, 115.6 (d, *J* = 23 Hz), 114.6 (d, *J* = 7 Hz), 80.0, 72.1, 54.8, 47.8, 45.9, 36.5, 28.3, 8.6 ppm. IR: 3358, 1683, 1170, 820, 755, 703 cm^–1^. HRMS (ESI-TOF) *m*/*z*: [M + H]^+^ calcd for C_21_H_28_FN_2_O_3_ 375.2078; found, 375.2080.

## Supplementary Material



## Data Availability

The data underlying this study are available in the published article and its Supporting Information.

## Notes

Including all ^1^H and ^13^C NMR spectroscopic data for all reported compounds (PDF)
