# Supplementary material for: Asymmetric Synthesis of the HIV Protease Inhibitor TMC-126, a PMX Antimalarial Protease Inhibitor, and a Putative COVID-19 Inhibitor Using a Highly Stereoselective Glycolate Aldol Addition Reaction Pathway
Source: ACS Omega. 2026 May 1;11(18):27331–41. doi: 10.1021/acsomega.6c01499 (PMC13177256; doi:10.1021/acsomega.6c01499)
Supplement: Supplementary file 1 [file ao6c01499_si_001.pdf]

Supporting Information

Asymmetric synthesis of the HIV protease inhibitor TMC-126, a PMX anti-malarial protease inhibitor, and a putative COVID-19 inhibitor using a highly stereoselective glycolate aldol addition reaction pathway

Kweku Amaning Affram, Austin Carter, April Breede, Alexandria Kimsey, Godson Hemeson, Bader Semakieh, Moses Martinez, Emmanuel Ayim, Joy Odeh, and Shawn R. Hitchcock*

Department of Chemistry, Illinois State University, Normal, IL 61790-4160

Table of Contents

Figure S1. 500 MHz ^{1}H NMR spectrum of **9** in $CDCl_3$……………………………………….……..S4

Figure S2. 100 MHz $^{13}C\{^1H\}$ NMR spectrum of **9** in $CDCl_3$…………………………..……....S5

Figure S3. 400 MHz ^{1}H NMR spectrum of **8** in $CDCl_3$……………………………………….……..S6

Figure S4. 100 MHz $^{13}C\{^1H\}$ NMR spectrum of **8** in $CDCl_3$……………………….…...……..S7

Figure S5. 400 MHz ^{1}H NMR spectrum of **10** in $CDCl_3$…………………………….…..……..S8

Figure S6. 100 MHz $^{13}C\{^1H\}$ NMR spectrum of **10** in $CDCl_3$…………………….…………..S9

Figure S7. 400 MHz ^{1}H NMR spectrum of **7** in $CDCl_3$……………………………….………..S10

Figure S8. 100 MHz $^{13}C\{^1H\}$ NMR spectrum of **7** in $CDCl_3$………………………………..S11

Figure S9. 400 MHz ^{1}H NMR spectrum of **6** in $CDCl_3$ ………………………………...…..S12

Figure S10. 100 MHz $^{13}C\{^{1}H\}$ NMR spectrum of **6** in $CDCl_3$………………..……………...S13

Figure S11. 400 MHz ^{1}H NMR spectrum of **3** in $CDCl_3$…………..……………………....S14

Figure S12. 100 MHz $^{13}C\{^{1}H\}$ NMR spectrum of **3** in $CDCl_3$ …………...……….………...S15

Figure S13. 400 MHz ^{1}H NMR Spectrum of **16** in $CDCl_3$…………..……………………....S16

Figure S14. 100 MHz $^{13}C\{^{1}H\}$ NMR spectrum of **16** in CDCl3…………...………………...S17

Figure S15. 400 MHz ^{1}H NMR Spectrum of **17** in $CDCl_3$…………..……………………....S18

Figure S16. 100 MHz $^{13}C\{^{1}H\}$ NMR spectrum of **17** in $CDCl_3$…………...………….……...S19

Figure S17. 400 MHz ^{1}H NMR Spectrum of **15** in $CDCl_3$…………..……………………....S20

Figure S18. 100 MHz $^{13}C\{^{1}H\}$ NMR spectrum of **15** in $CDCl_3$…………...………….……...S21

Figure S19. 400 MHz ^{1}H NMR Spectrum of **19** in $CDCl_3$…………..……………………....S22

Figure S20. 100 MHz $^{13}C\{^{1}H\}$ NMR spectrum of **19** in $CDCl_3$…………...………………...S23

Figure S21. 400 MHz ^{1}H NMR Spectrum of **14** in $CDCl_3$…………..……………………...S24

Figure S22. 100 MHz $^{13}C\{^{1}H\}$ NMR spectrum of **14** in $CDCl_3$…………...………………...S25

Figure S23. 400 MHz ^{1}H NMR Spectrum of **21** in $CDCl_3$…………..……………………...S26

Figure S24. 100 MHz $^{13}C\{^{1}H\}$ NMR spectrum of **21** in $CDCl_3$…………...……….……....S27

Figure S25. 400 MHz ^{1}H NMR Spectrum of **13** in D_2O…………..………………………......S28

Figure S26. 100 MHz $^{13}C\{^{1}H\}$ NMR spectrum of **13** in D_2O…………...………….……....S29

Figure S27. 400 MHz ^{1}H NMR Spectrum of **4** in $CDCl_3$…………..…………………...…...S30

Figure S28. 100 MHz $^{13}C\{^{1}H\}$ NMR spectrum of **4** in $CDCl_3$…………...………….……...S31

Figure S29. 500 MHz ^{1}H NMR Spectrum of **24** in $CDCl_3$…………..…………………..…..S32

Figure S30. 100 MHz $^{13}C\{^{1}H\}$ NMR spectrum of **24** in $CDCl_3$…………...………………...S33

Figure S31. 400 MHz ^{1}H NMR Spectrum of **27** in $CDCl_3$…………..……………..............S34

Figure S32. 100 MHz $^{13}C\{^{1}H\}$ NMR spectrum of **27** in $CDCl_3$…………...……….....……...S35

Figure S33. 400 MHz ^{1}H NMR Spectrum of **28** in $CDCl_3$…………..………………...…....S36

Figure S34. 100 MHz $^{13}C\{^{1}H\}$ NMR spectrum of **28** in $CDCl_3$…………...…….………....S37

Figure S35. 400 MHz ^{1}H NMR Spectrum of **23** in $CDCl_3$ …………..……………….….....S38

Figure S36. 400 MHz ^{1}H NMR Spectrum of **22** in $CDCl_3$…………..……………….….....S39

Figure S37. 100 MHz $^{13}C\{^1H\}$ NMR spectrum of **22** in $CDCl_3$…………...………………..S40
Figure S38. 500 MHz ^1H-^1H-COSY of **22** in $CDCl_3$…………...…………….…………...S41
Figure S39. 400 MHz ^1H NMR Spectrum of **5** in $CDCl_3$…………...…………………..…...S42
Figure S40. 100 MHz $^{13}C\{^1H\}$ NMR spectrum of **5** in $CDCl_3$…………...……..…………...S43
Figure S41. High resolution mass spectrum of the deprotection putative byproduct **32**…….S44

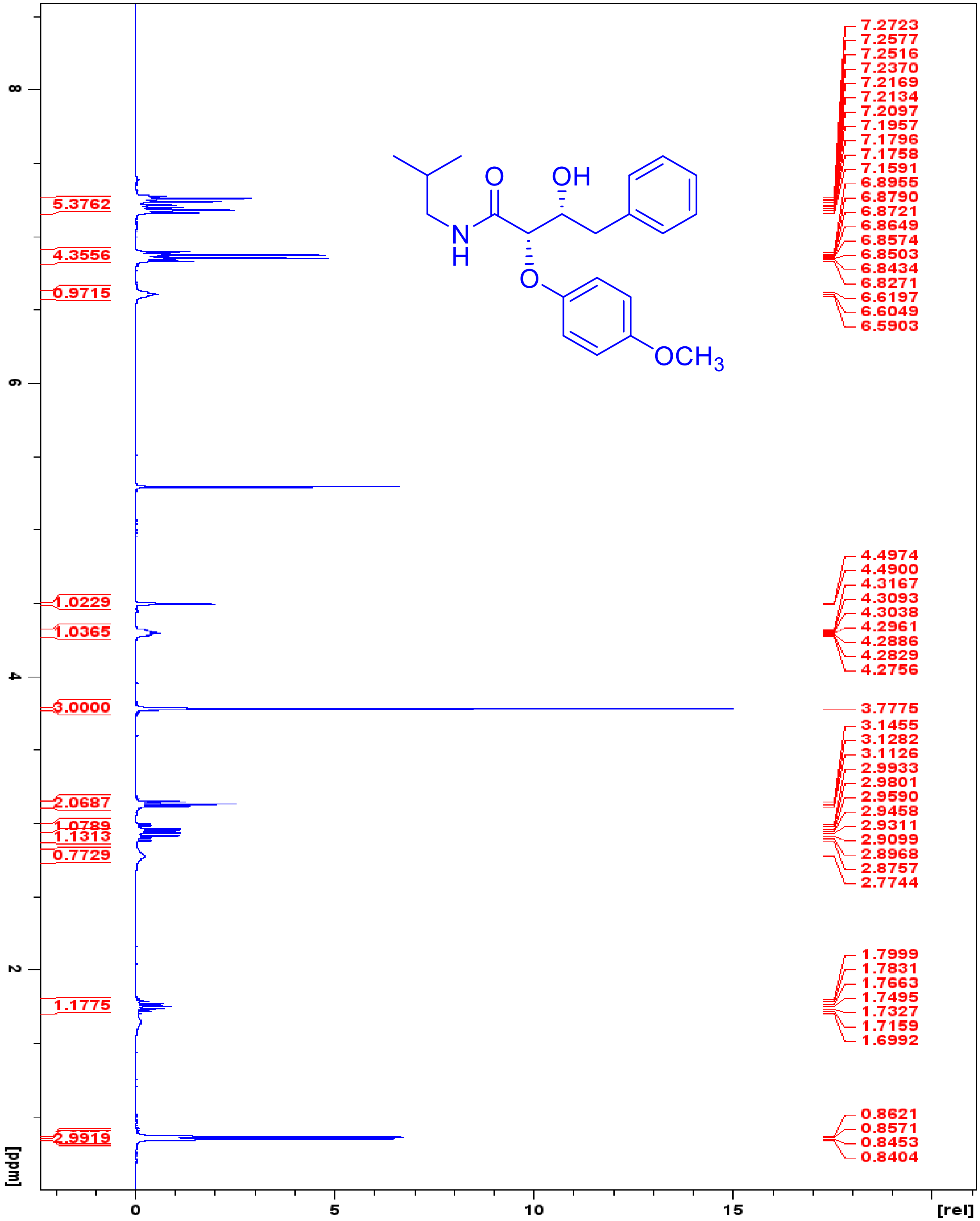

Figure S1. 500 MHz ^{1}H NMR spectrum of **9** in $CDCl_3$.

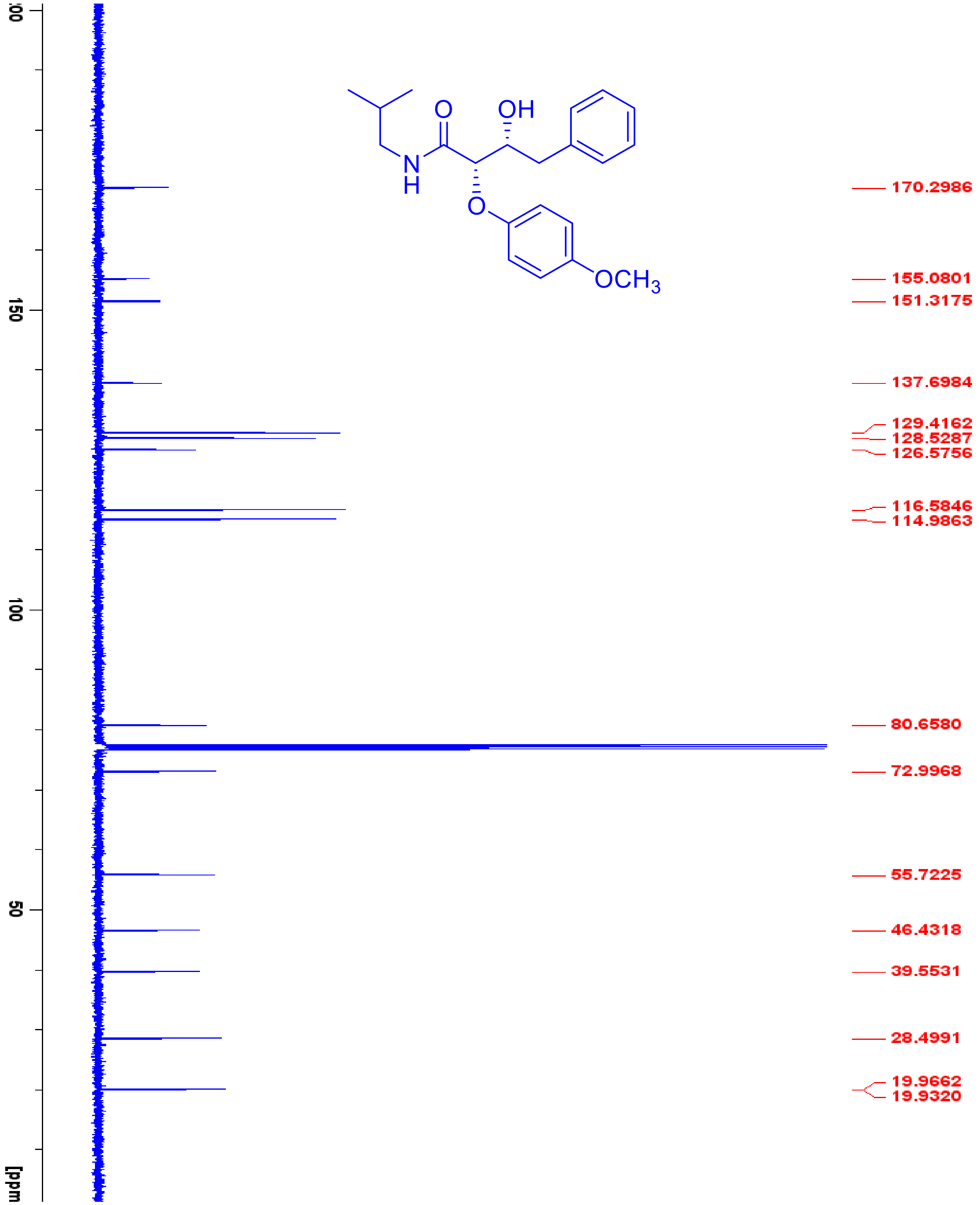

Figure S2. 100 MHz $^{13}C\{^{1}H\}$ NMR spectrum of **9** in $CDCl_3$.

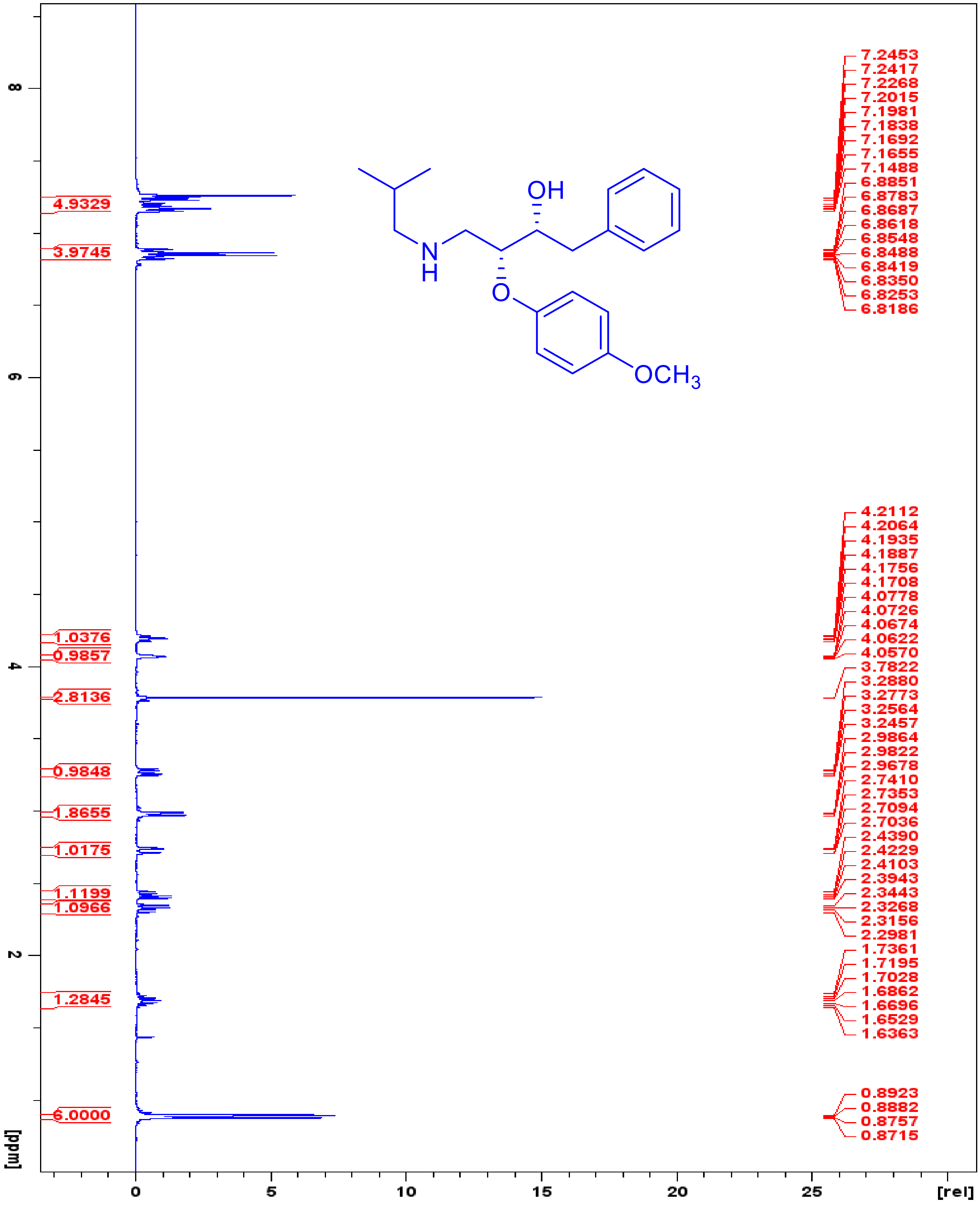

Figure S3. 400 MHz ^{1}H NMR spectrum of **8** in $CDCl_3$.

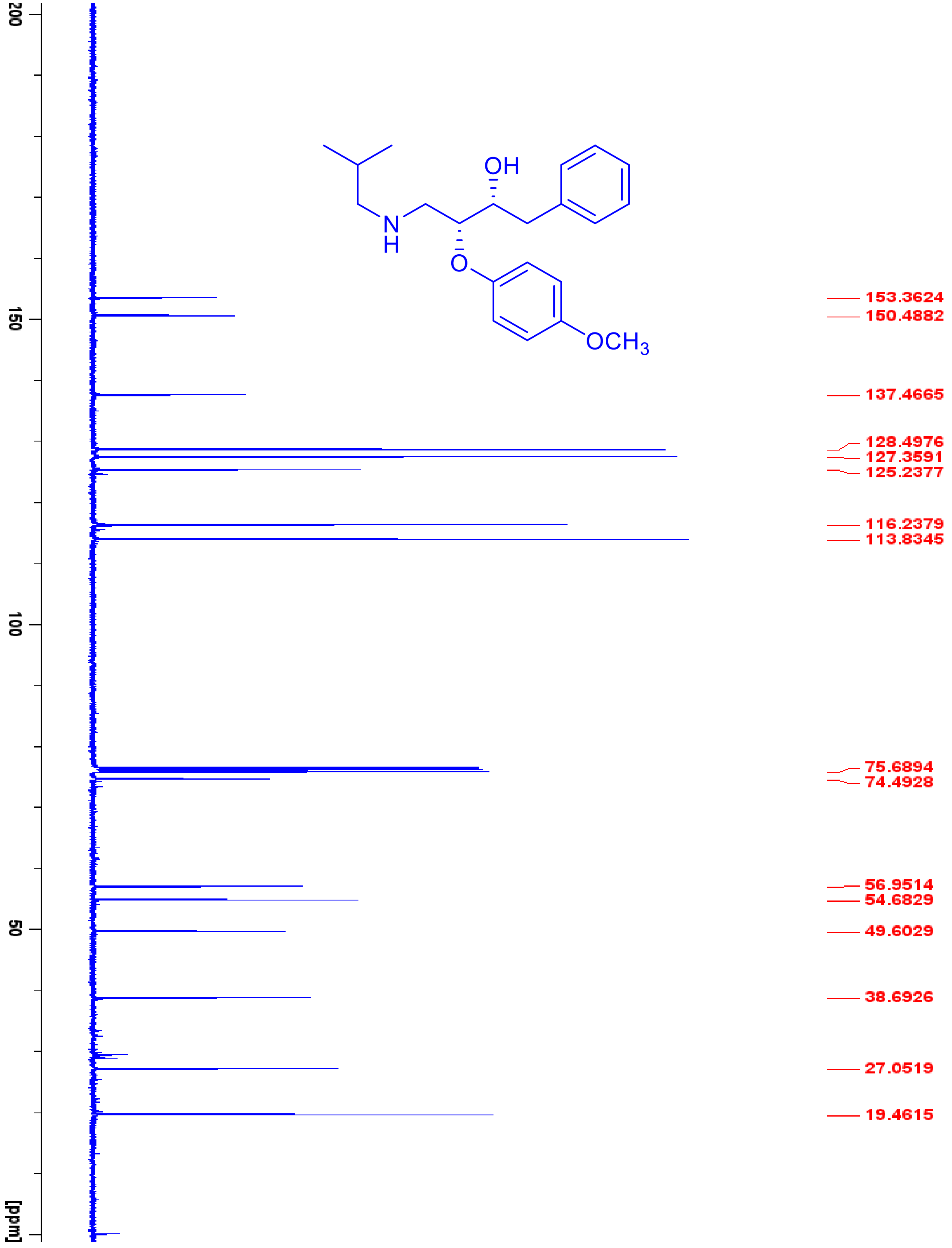

Figure S4. 100 MHz $^{13}C\{^{1}H\}$ NMR spectrum of **8** in $CDCl_3$.

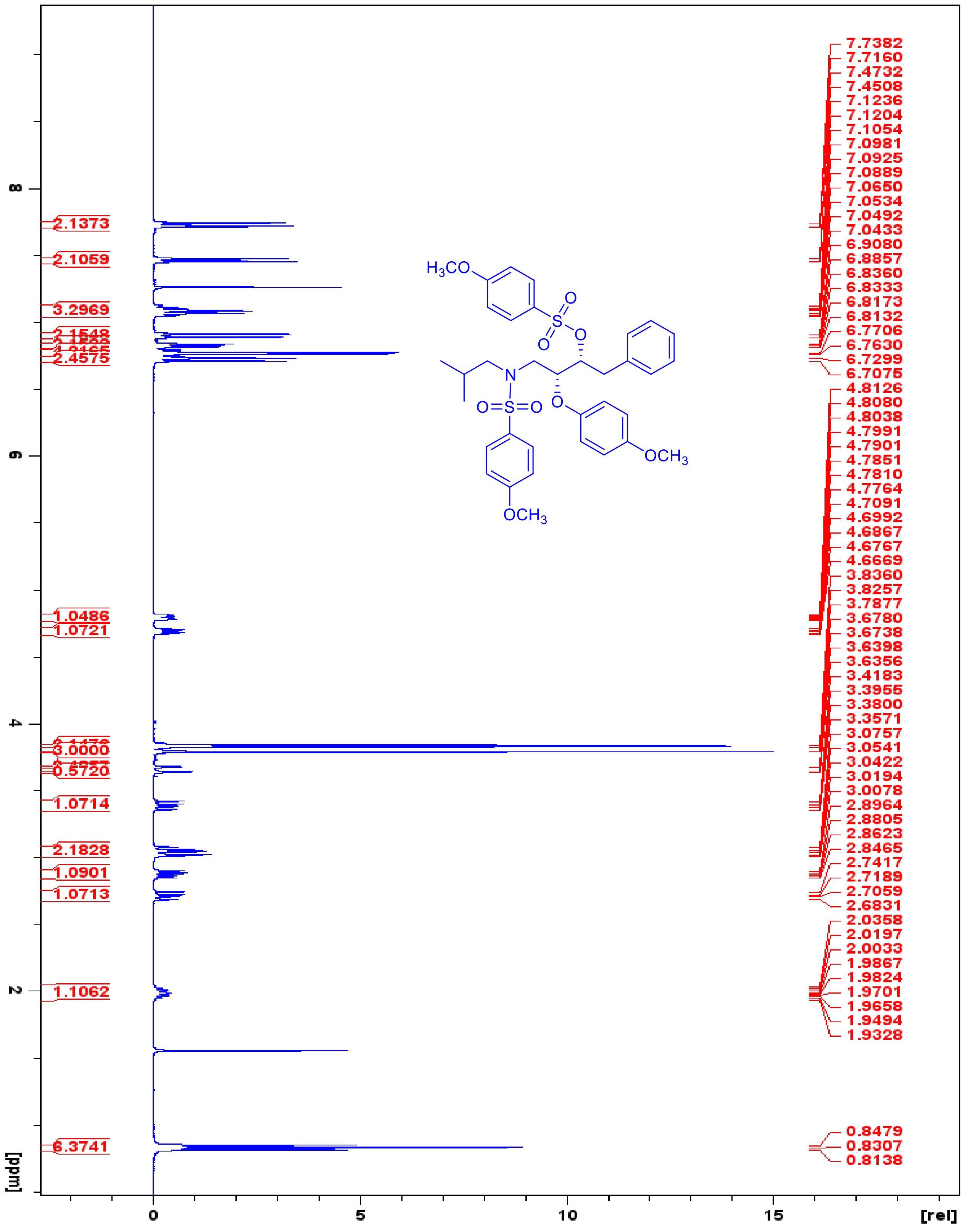

Figure S5. 400 MHz ^{1}H NMR spectrum of **10** in $CDCl_3$.

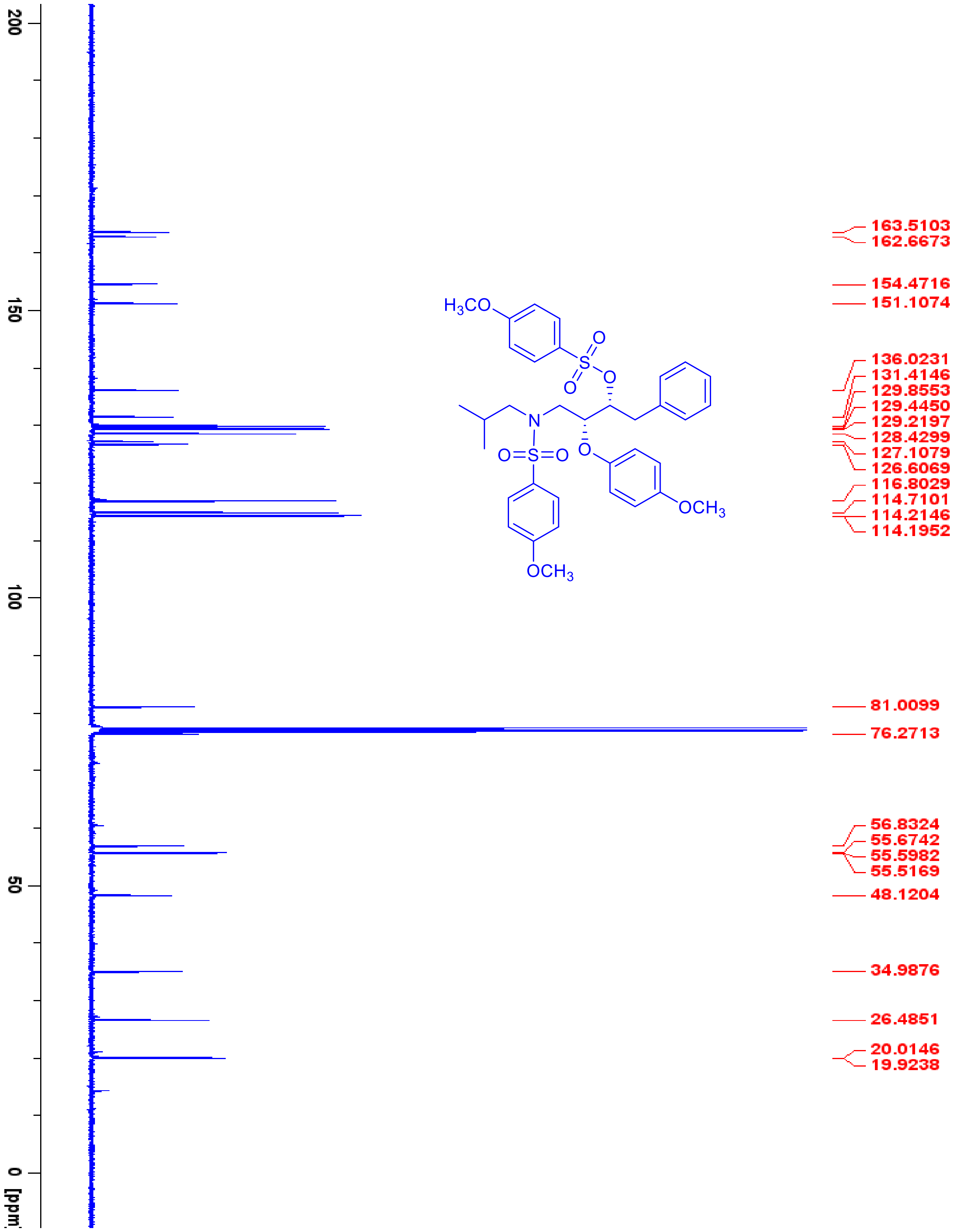

Figure S6. 100 MHz $^{13}C\{^{1}H\}$ NMR spectrum of **10** in $CDCl_3$.

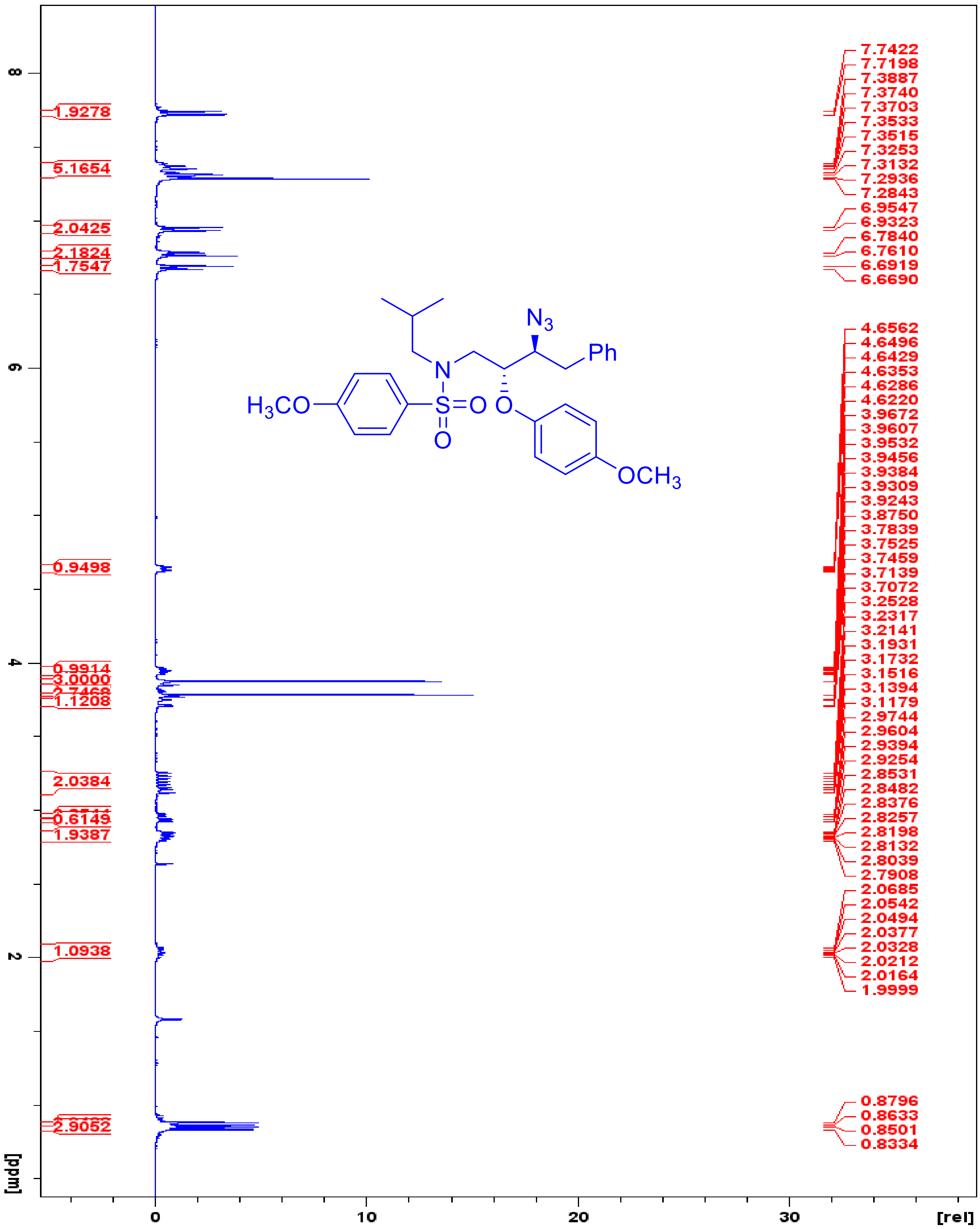

Figure S7. 400 MHz ^{1}H NMR spectrum of **7** in $CDCl_3$.

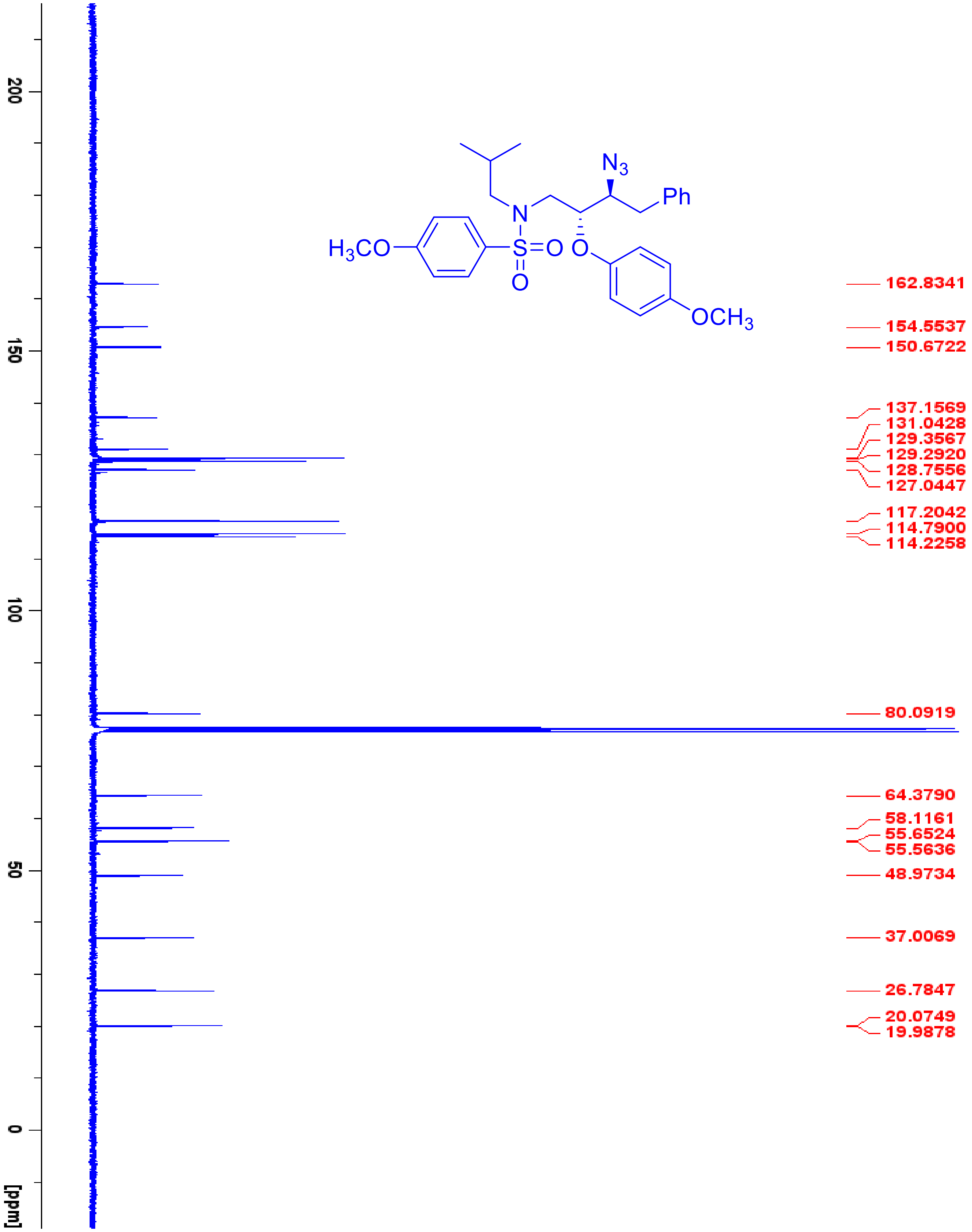

Figure S8. 100 MHz $^{13}C\{^1H\}$ NMR spectrum of **7** in $CDCl_3$.

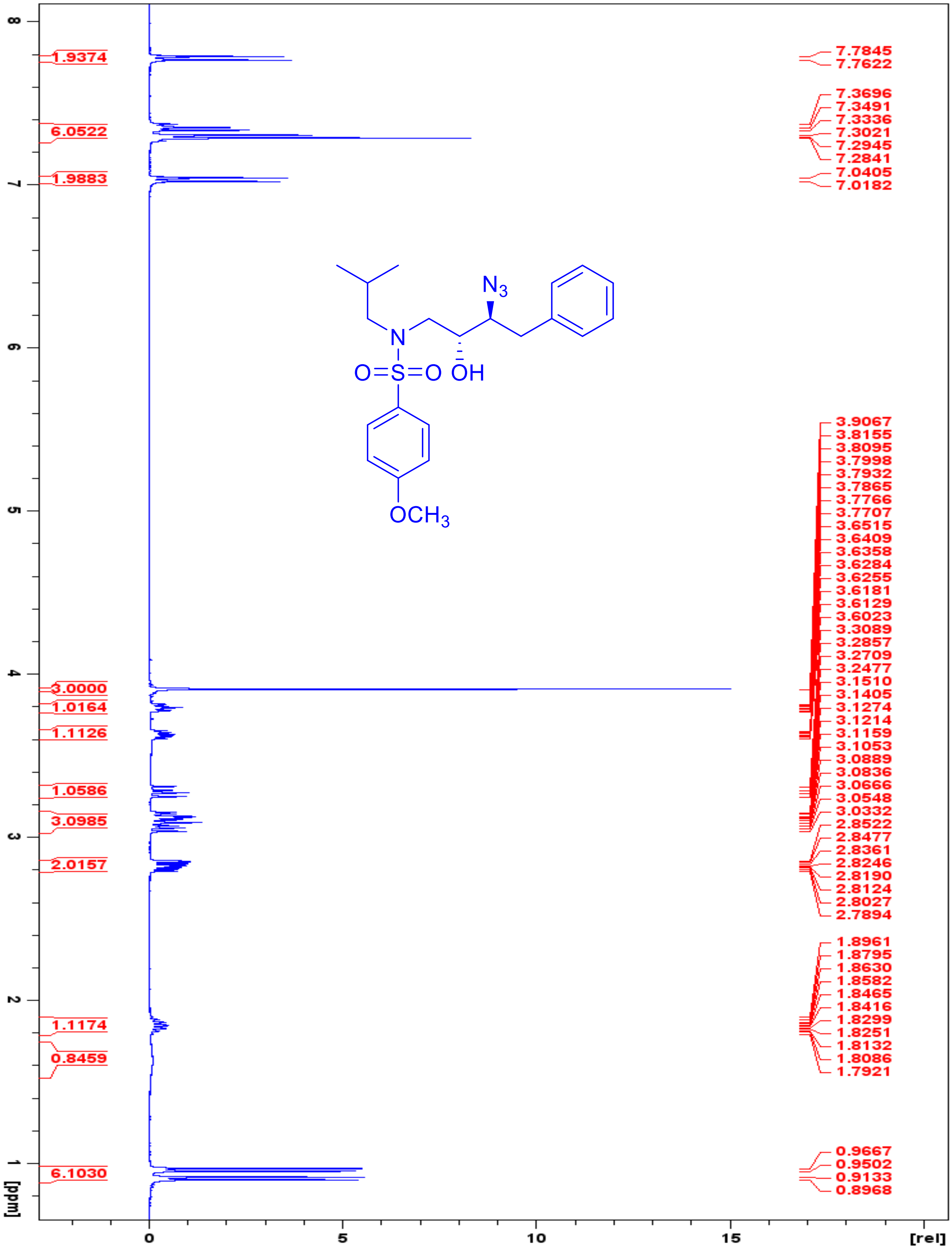

Figure S9. 400 MHz ^{1}H NMR spectrum of **6** in $CDCl_3$.

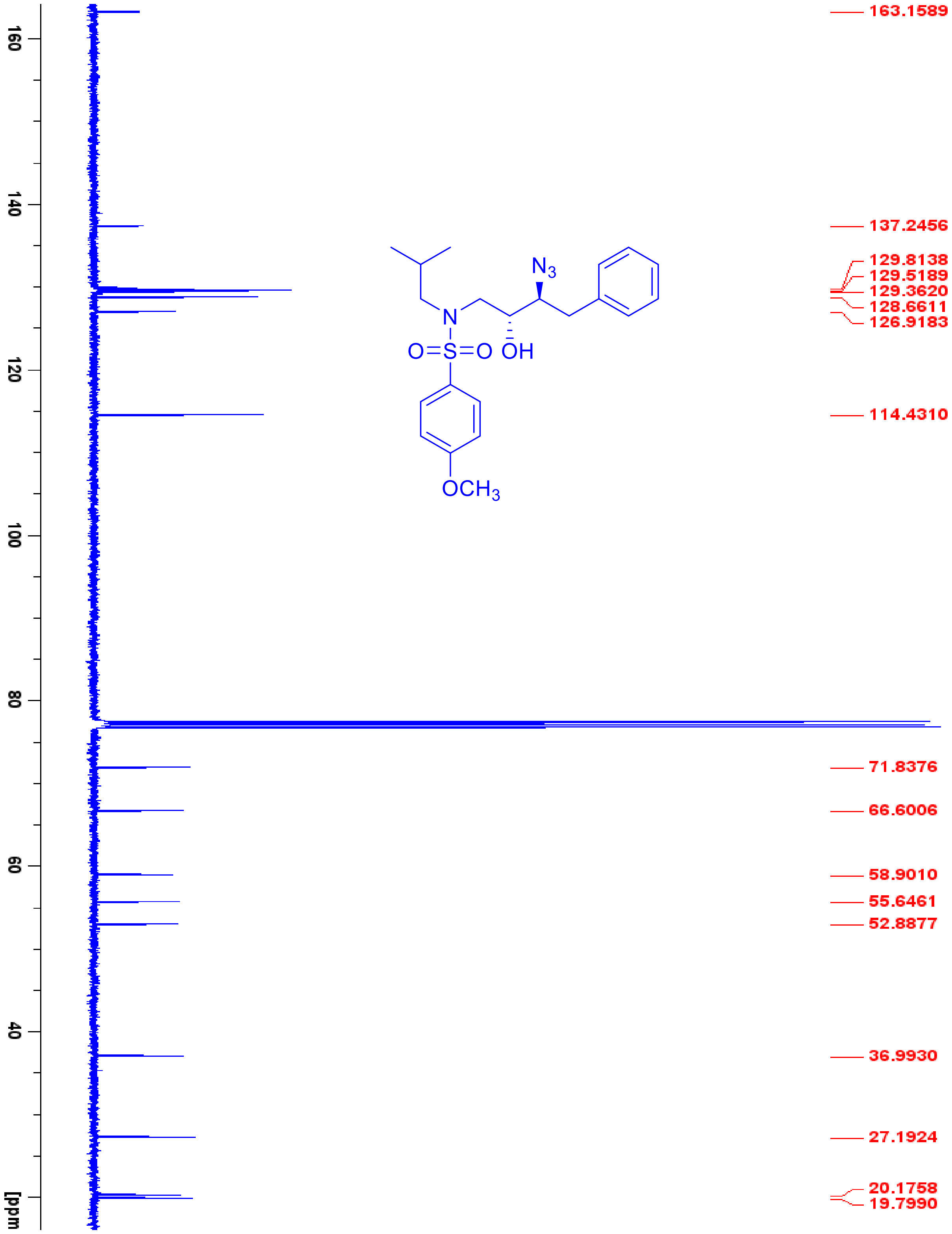

Figure S10. 100 MHz $^{13}C\{^{1}H\}$ NMR spectrum of **6** in $CDCl_3$.

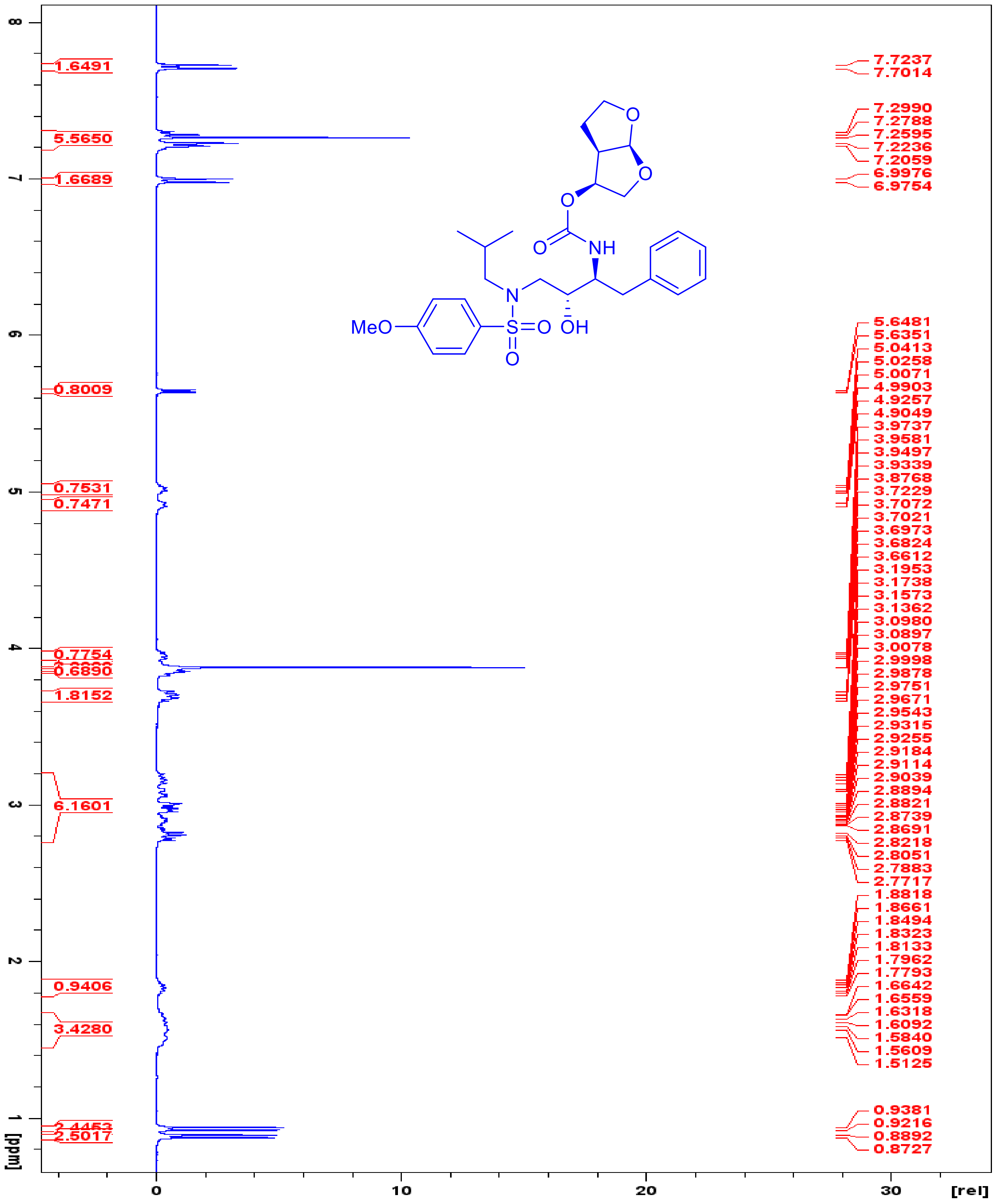

Figure S11. 400 MHz ^{1}H NMR spectrum of **3** in $CDCl_3$.

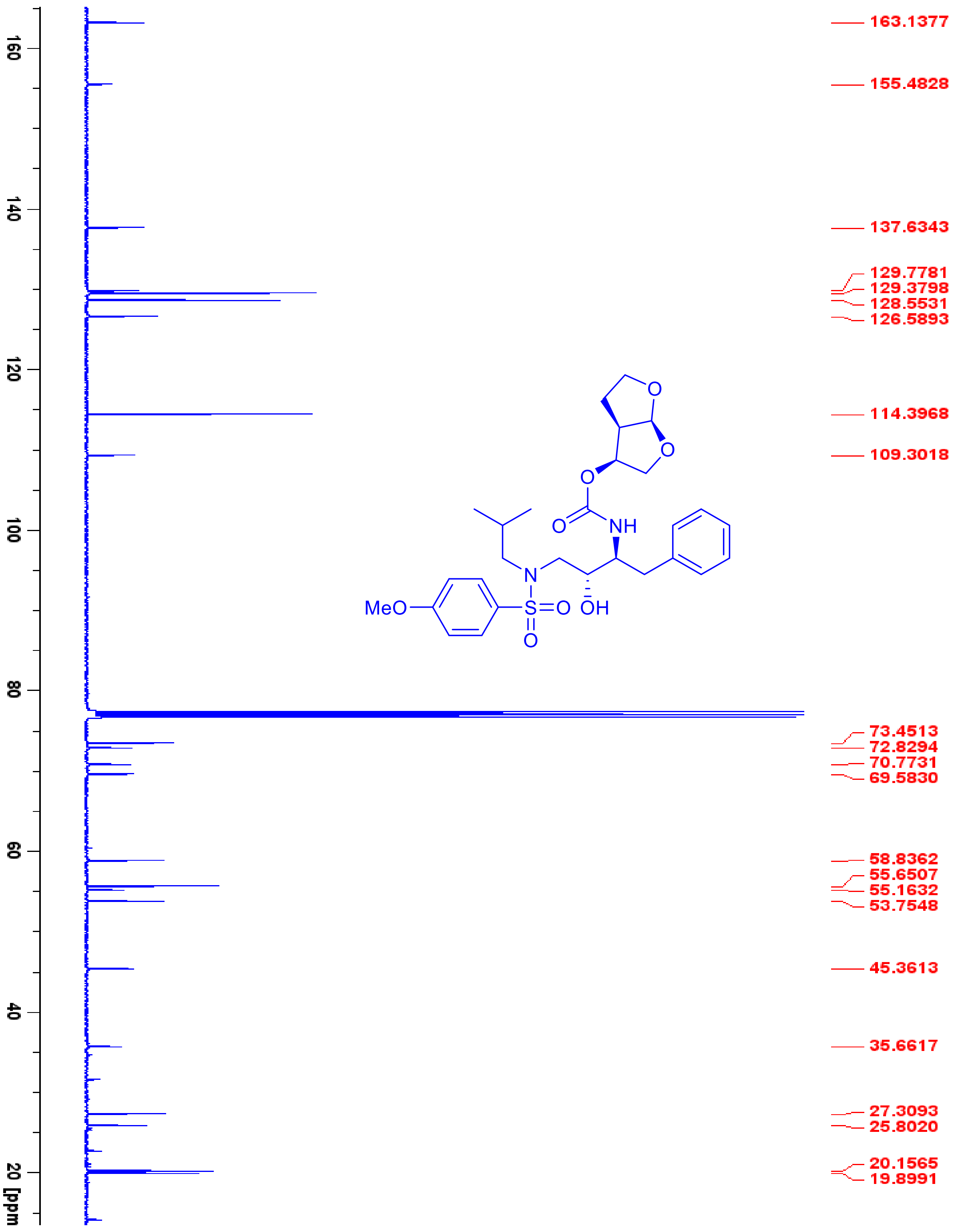

Figure S12. 100 MHz $^{13}C\{^{1}H\}$ NMR spectrum of **3** in $CDCl_3$.

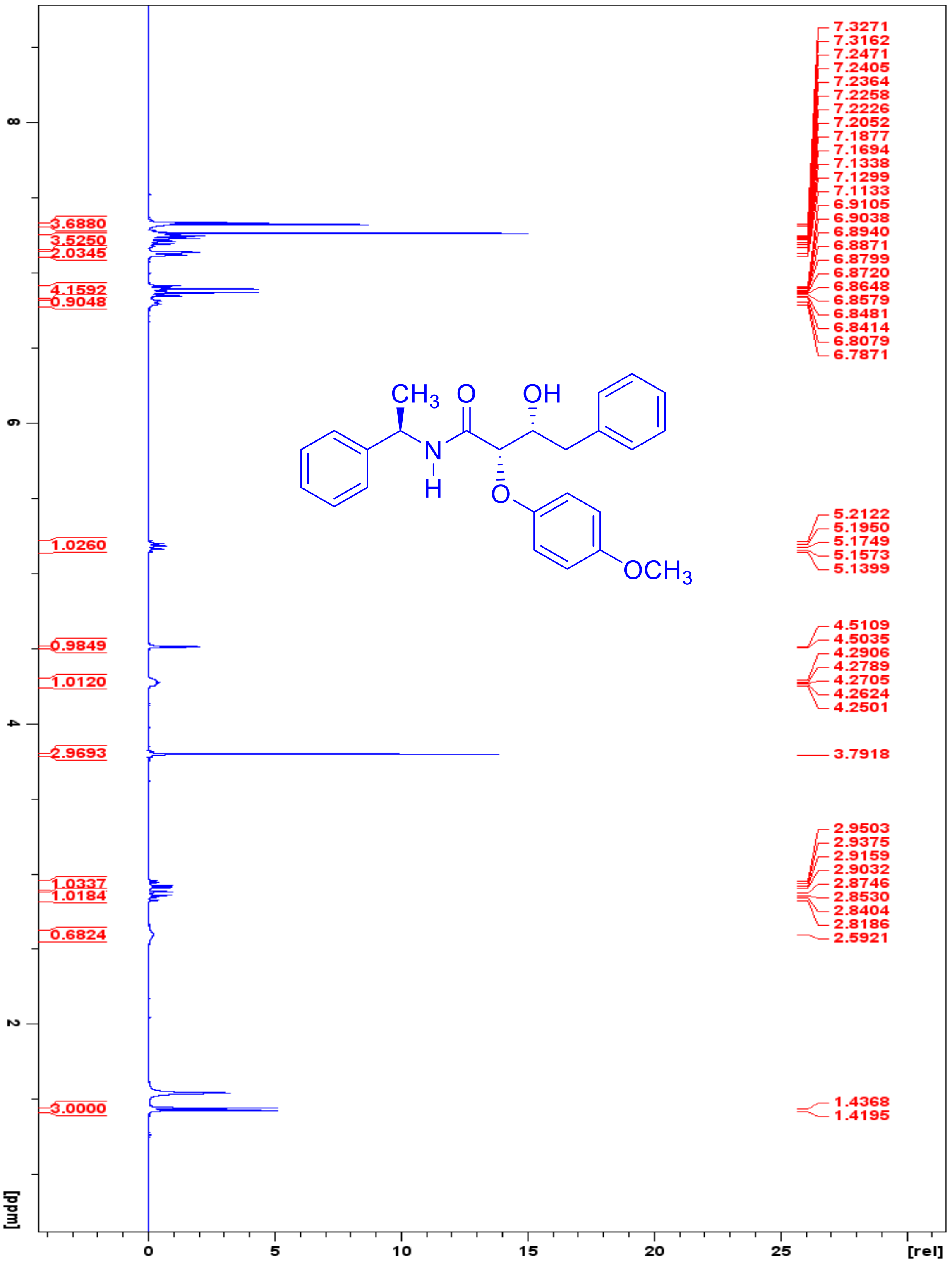

Figure S13. 400 MHz ^{1}H NMR Spectrum of **16** in $CDCl_3$.

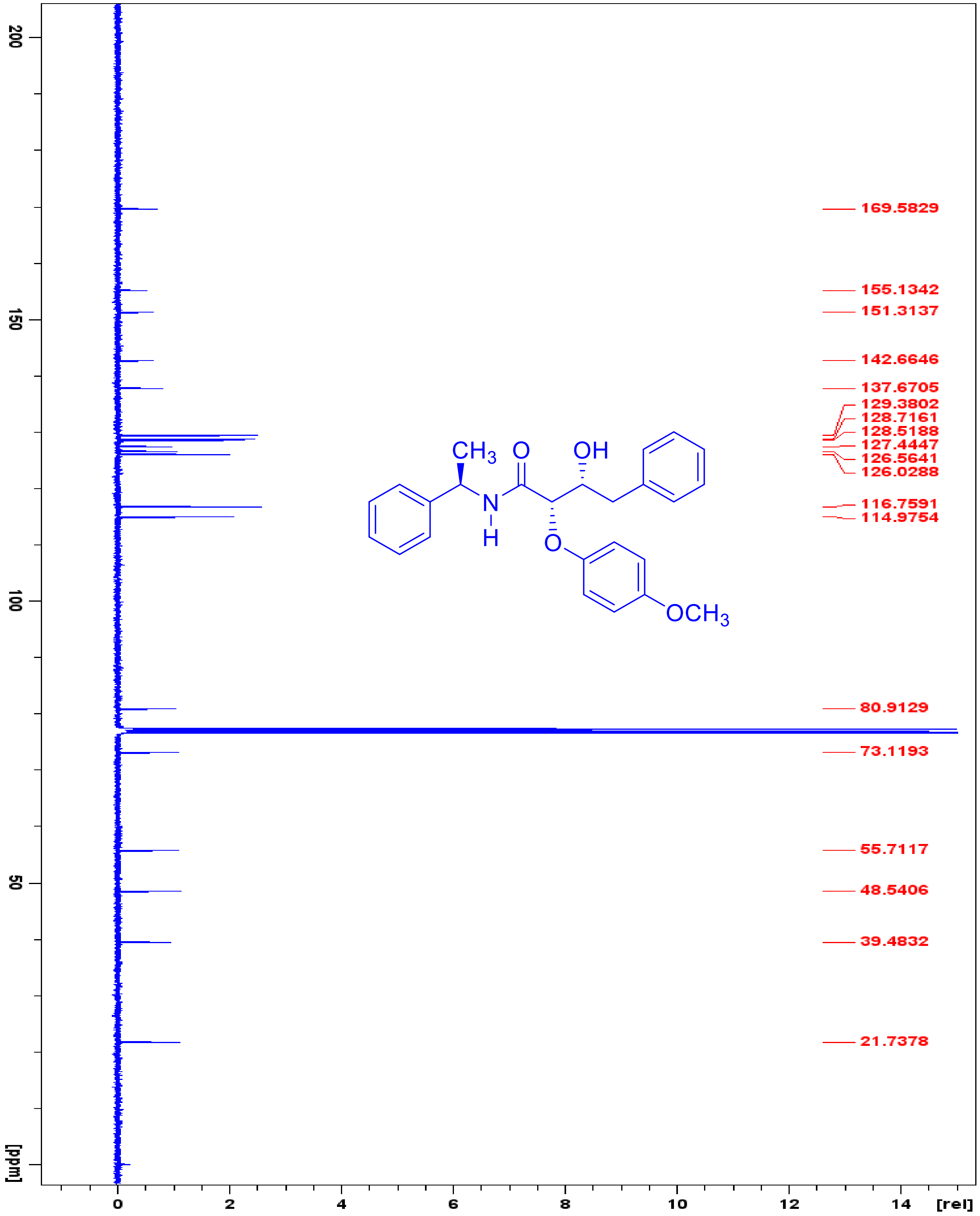

Figure S14. 100 MHz $^{13}C\{^{1}H\}$ NMR spectrum of **16** in $CDCl_3$.

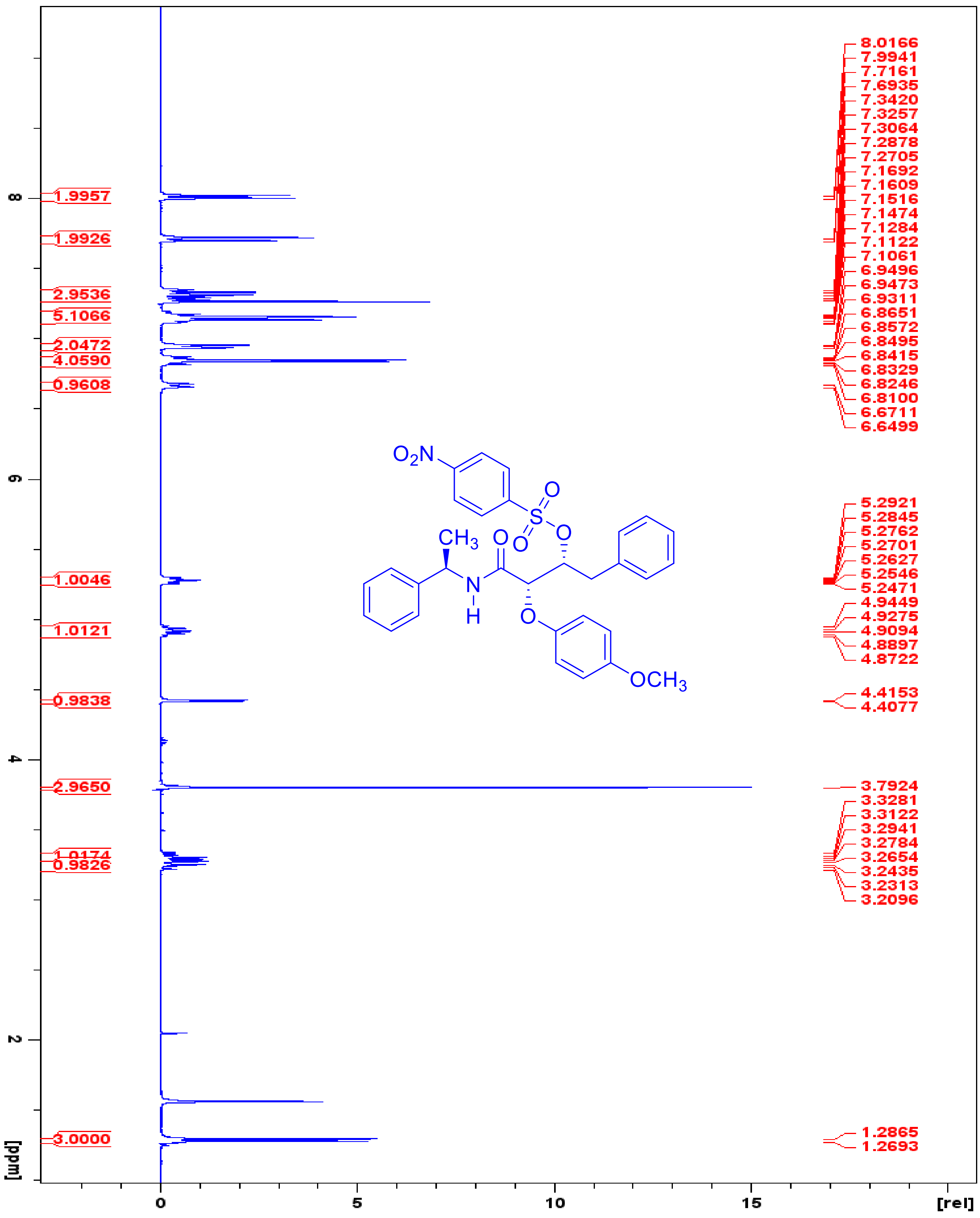

Figure S15. 400 MHz ^{1}H NMR Spectrum of **17** in $CDCl_3$.

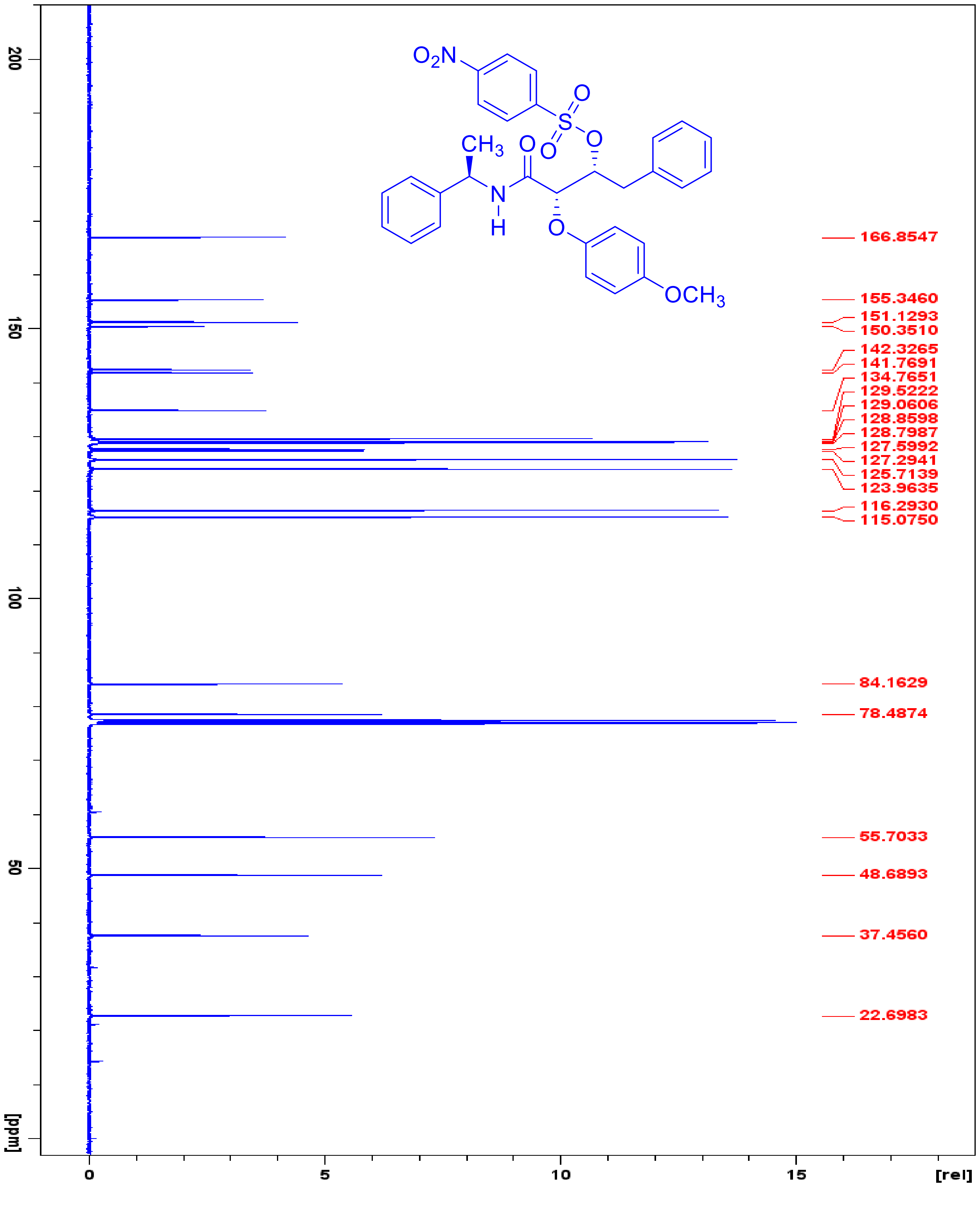

Figure S16. 100 MHz $^{13}C\{^1H\}$ NMR spectrum of **17** in $CDCl_3$.

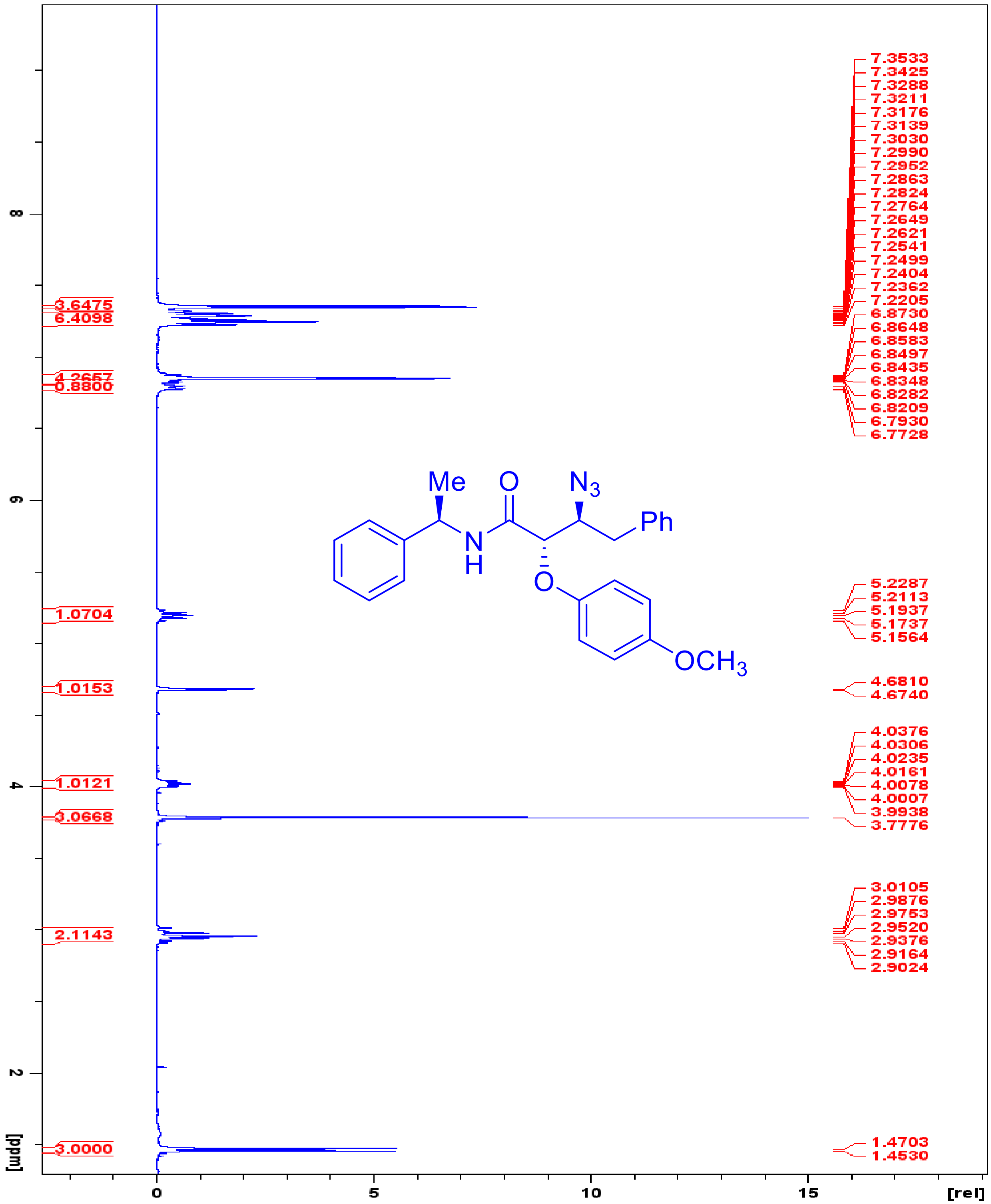

Figure S17. 400 MHz ^{1}H NMR Spectrum of **15** in $CDCl_3$.

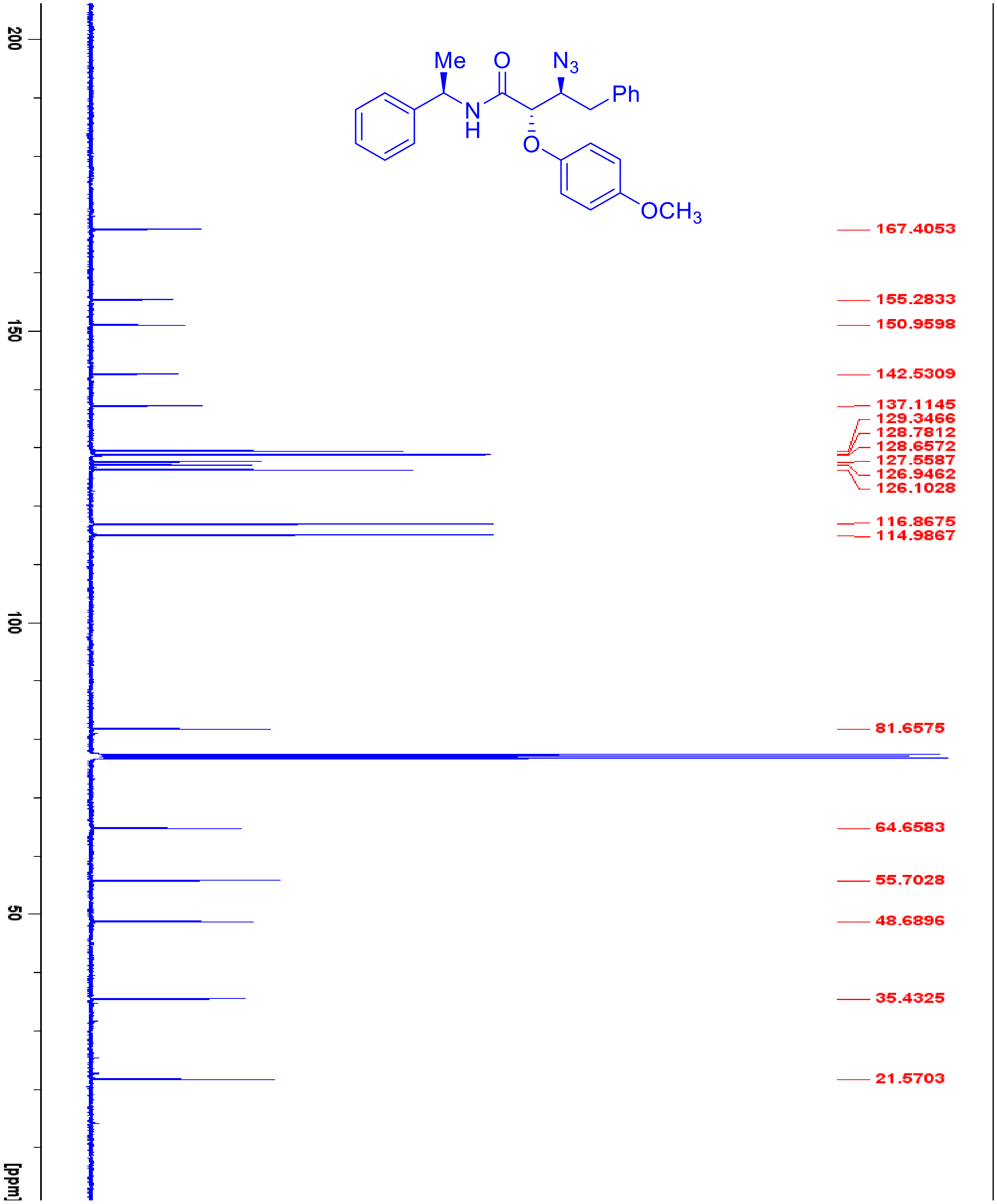

Figure S18. 100 MHz $^{13}C\{^{1}H\}$ NMR spectrum of **15** in $CDCl_3$.

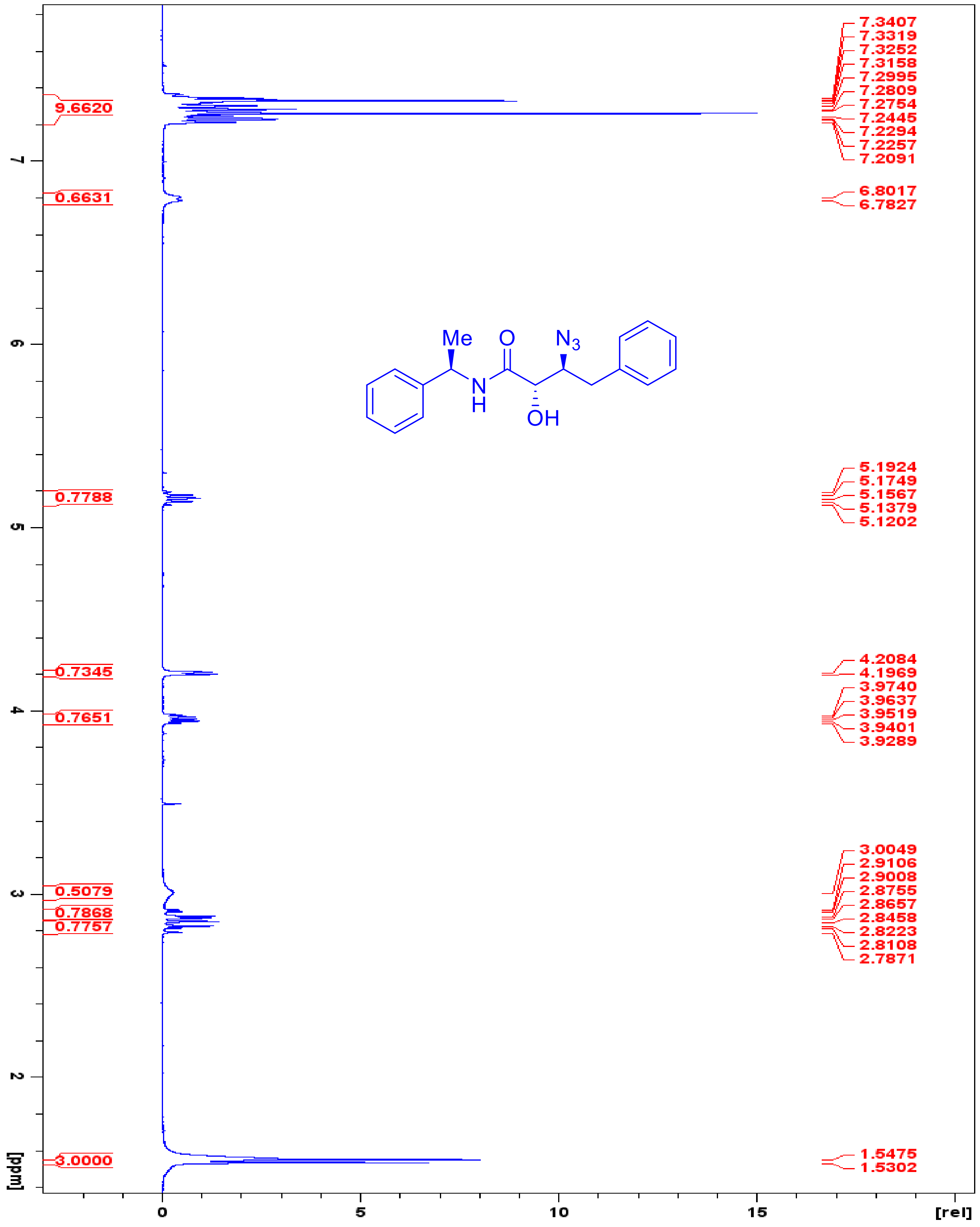

Figure S19. 400 MHz ^{1}H NMR Spectrum of **19** in $CDCl_3$.

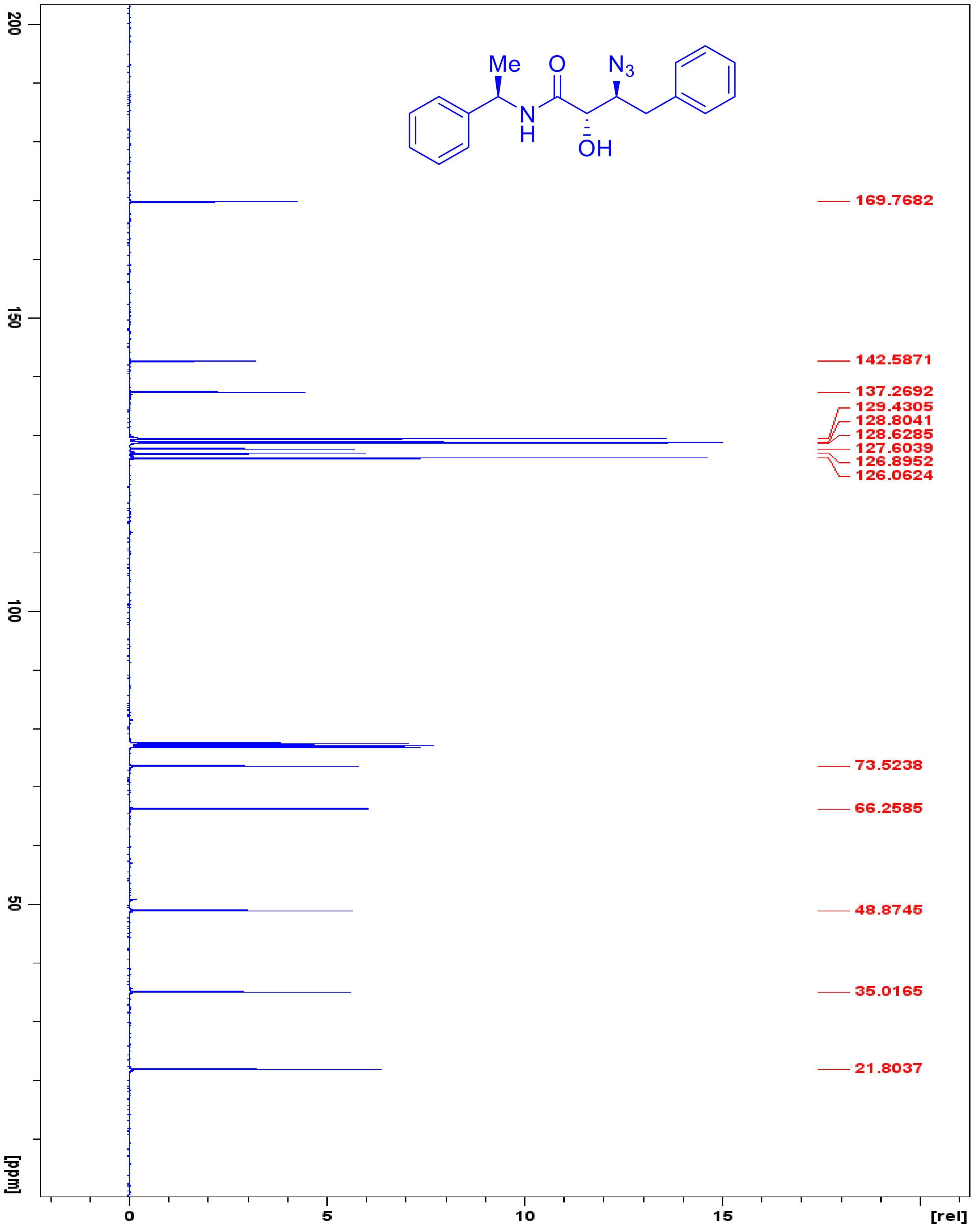

Figure S20. 100 MHz $^{13}C\{^{1}H\}$ NMR spectrum of **19** in $CDCl_3$.

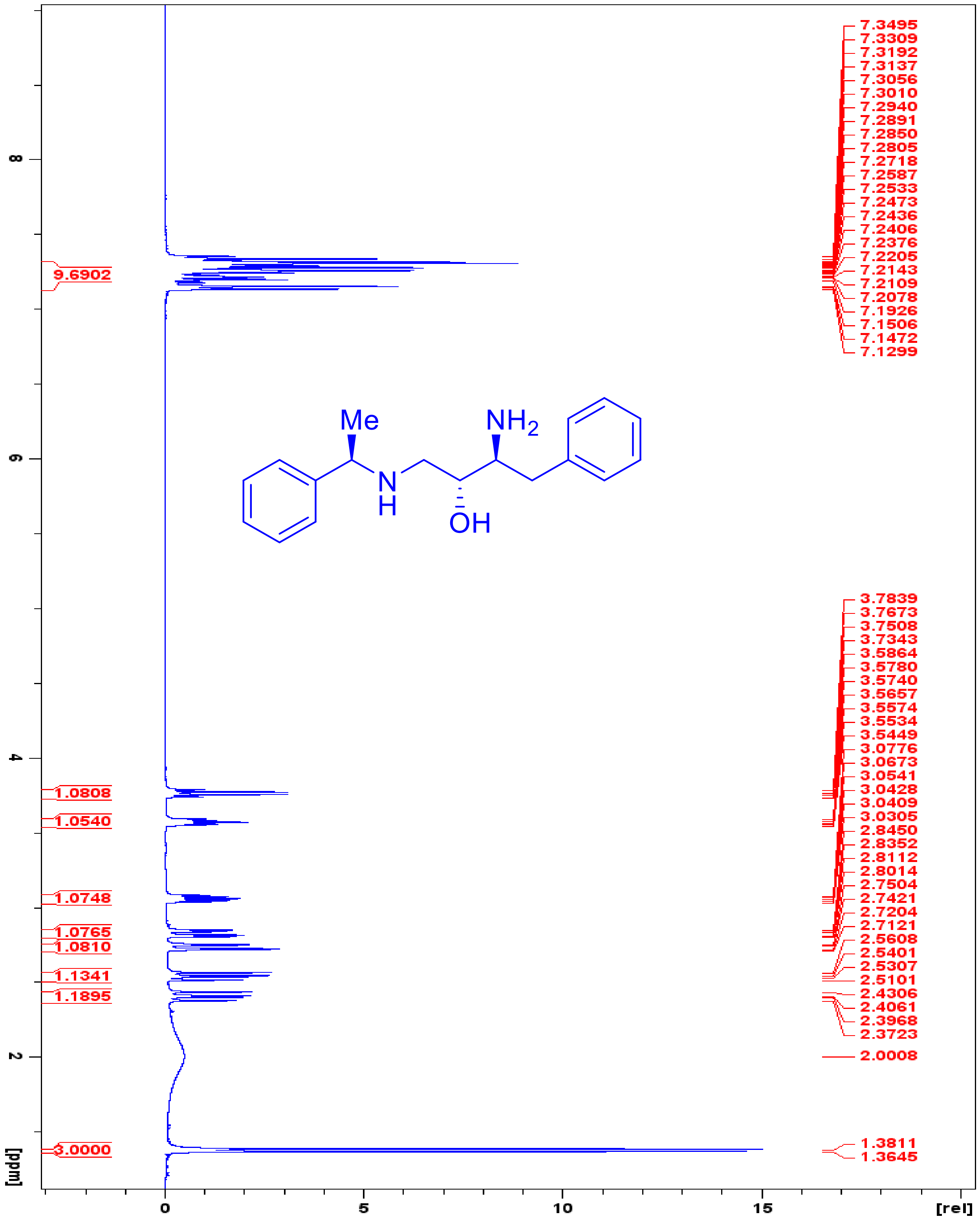

Figure S21. 400 MHz ^{1}H NMR Spectrum of **14** in $CDCl_3$.

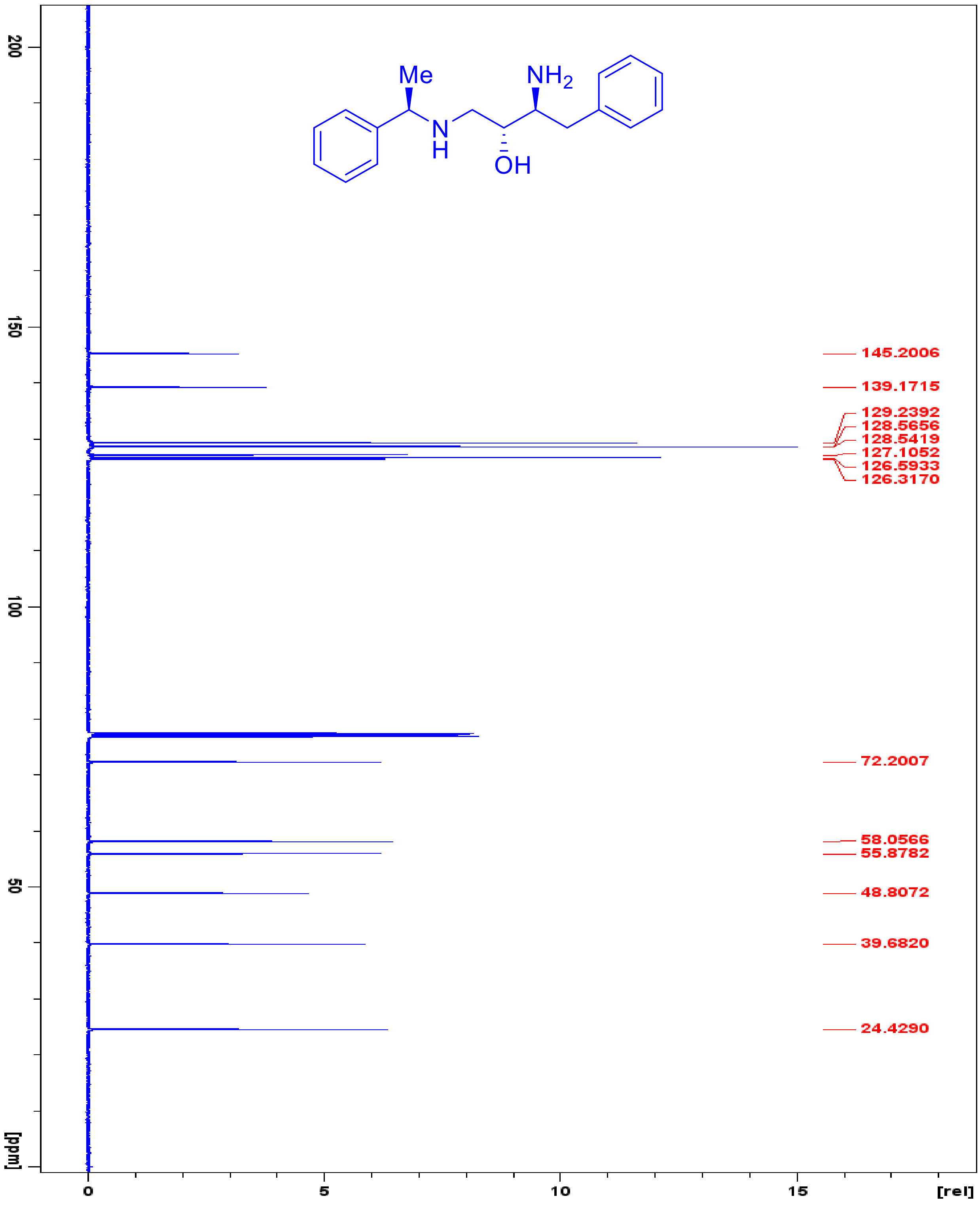

Figure S22. 100 MHz $^{13}C\{^{1}H\}$ NMR spectrum of **14** in $CDCl_3$.

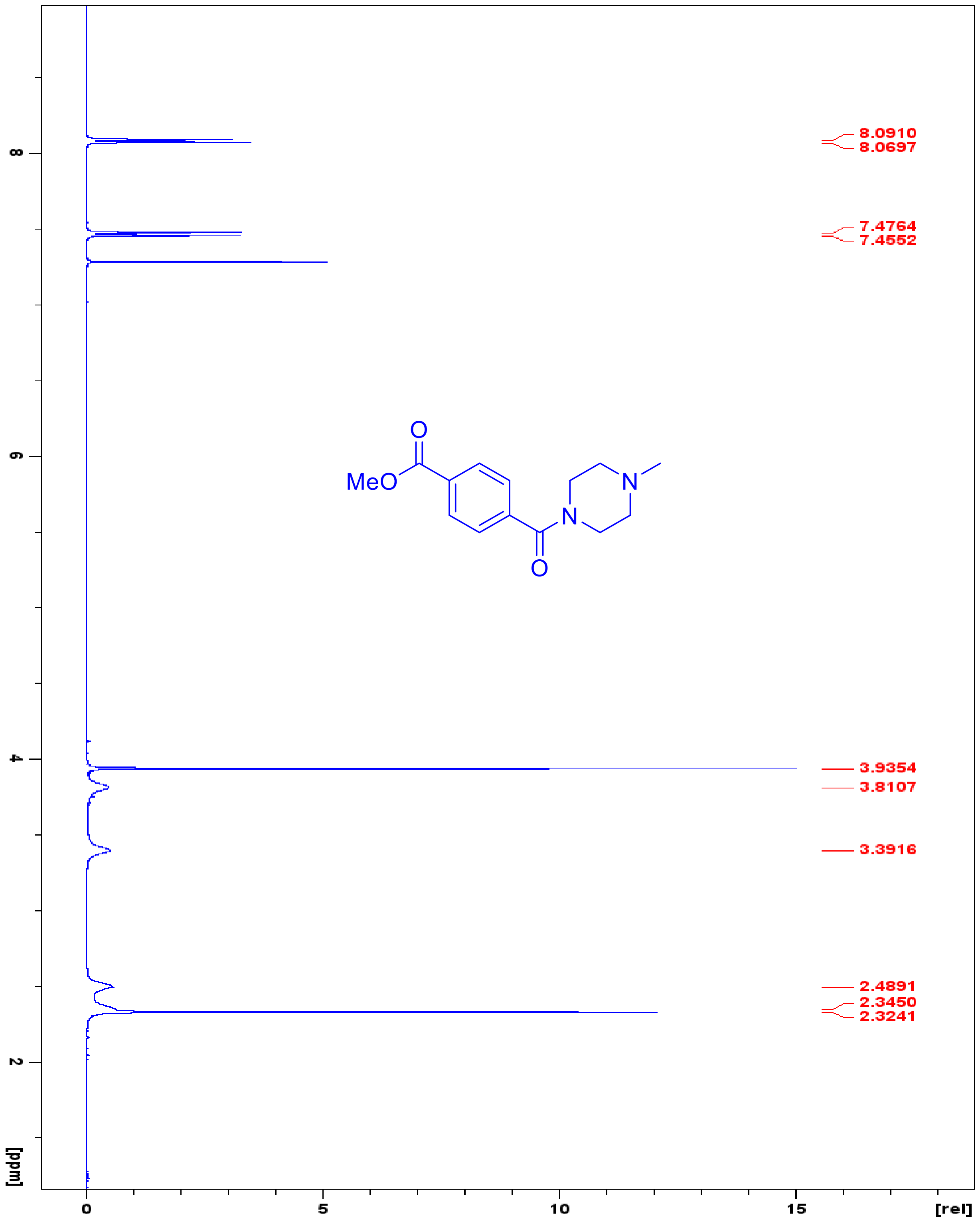

Figure S23. 400 MHz ^{1}H NMR Spectrum of **21** in $CDCl_3$.

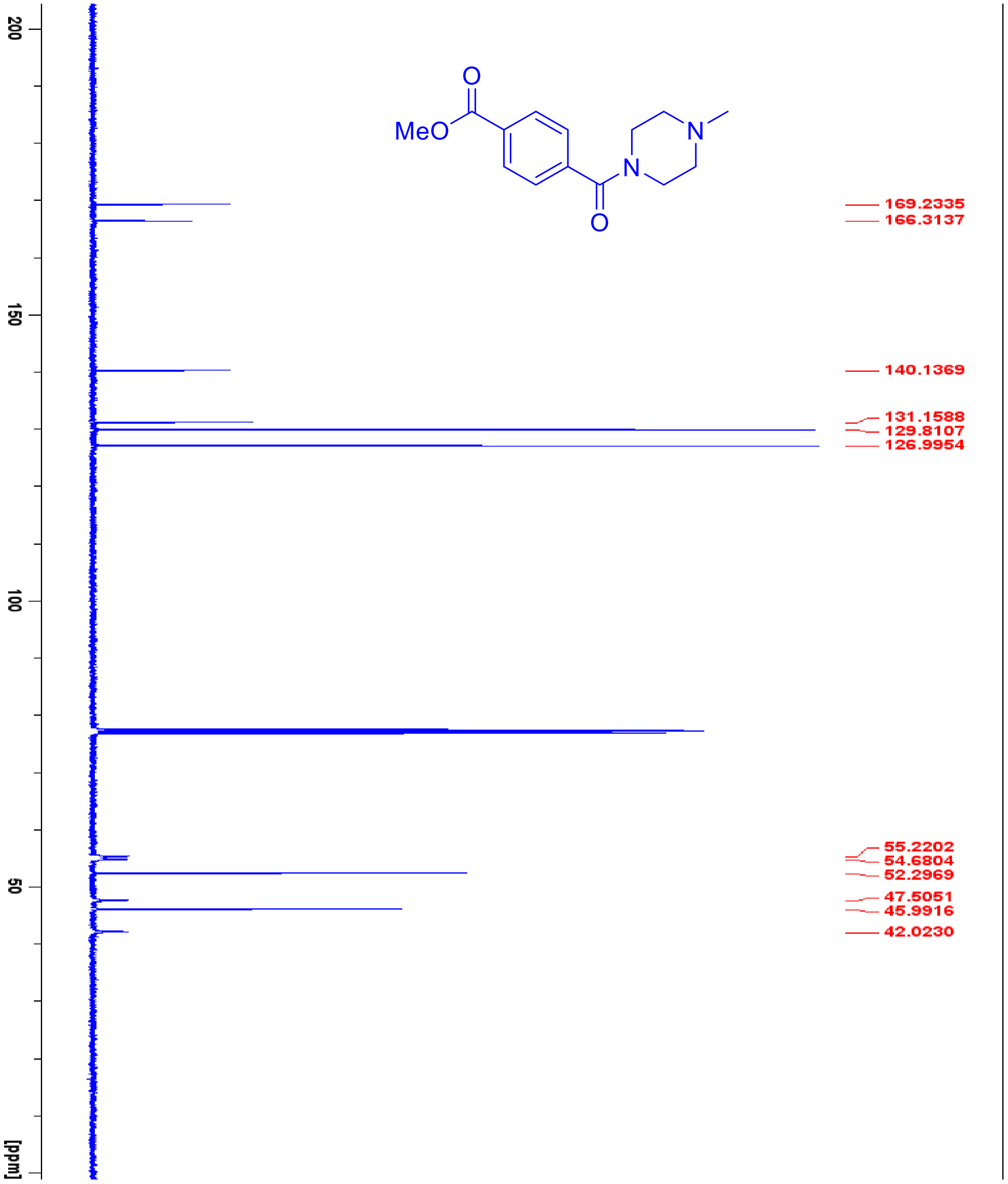

Figure S24. 100 MHz $^{13}C\{^{1}H\}$ NMR spectrum of **21** in $CDCl_3$.

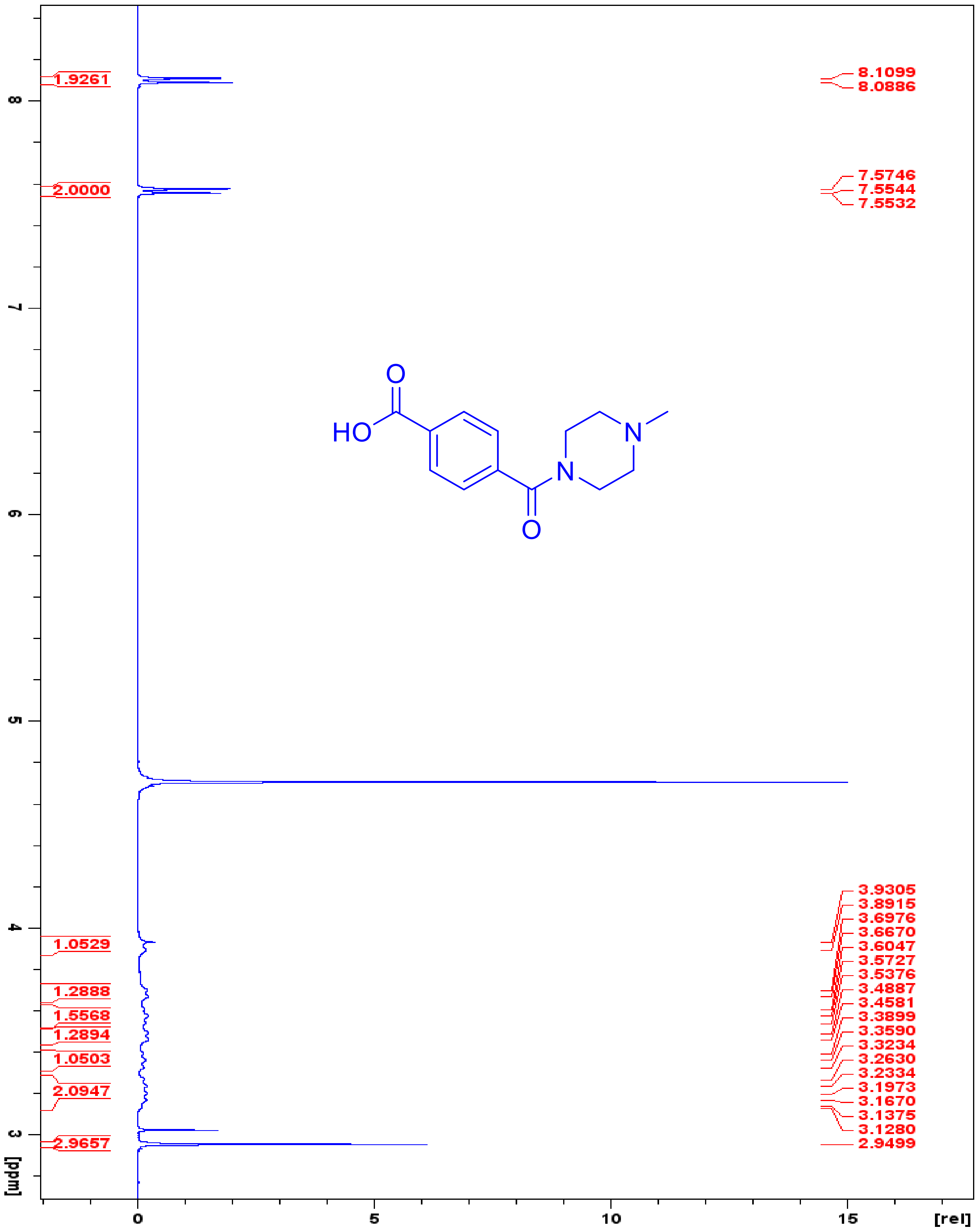

Figure S25. 400 MHz ¹H NMR Spectrum of **13** in D_2O.

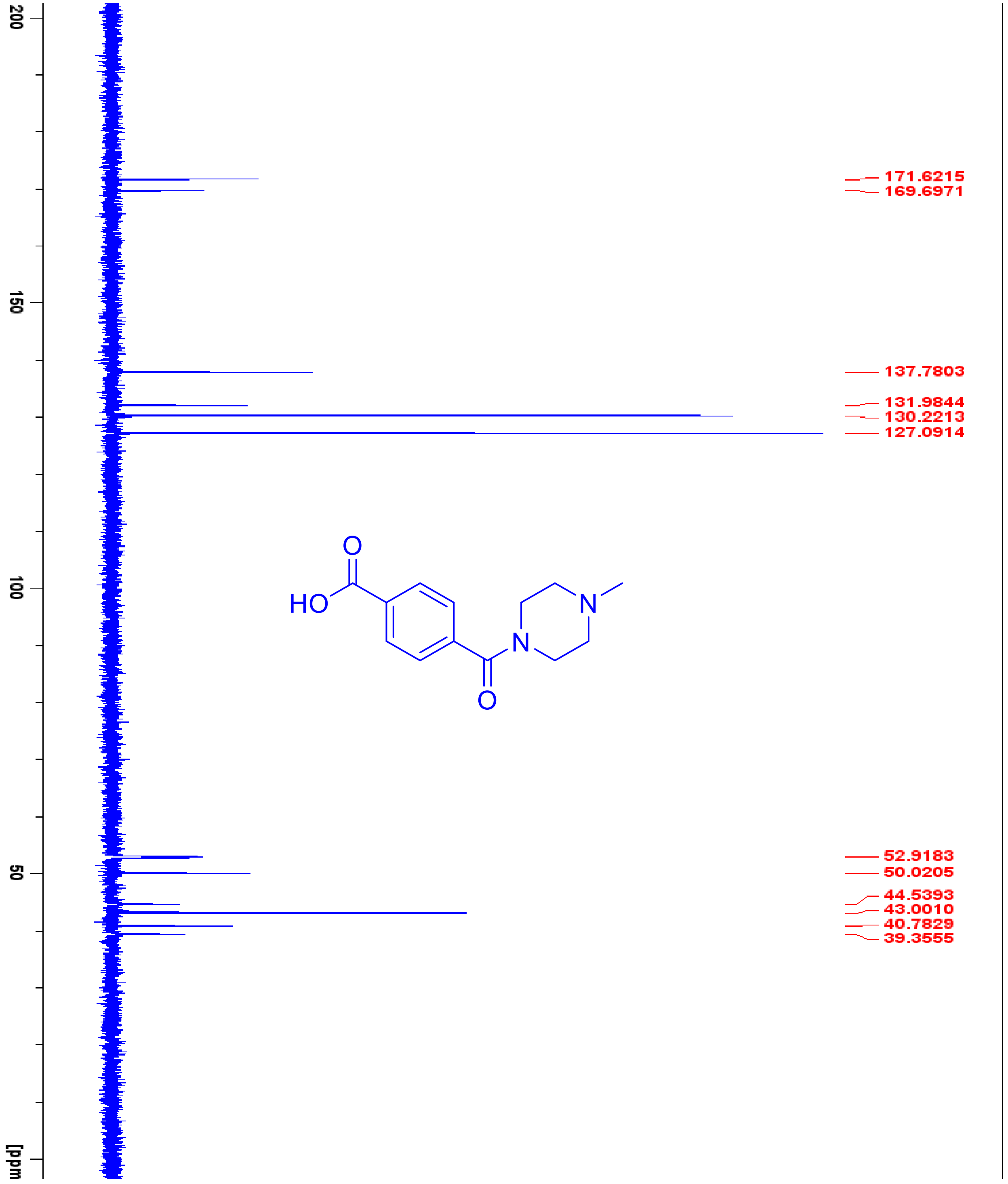

Figure S26. 100 MHz $^{13}C\{^{1}H\}$ NMR spectrum of **13** in D_2O.

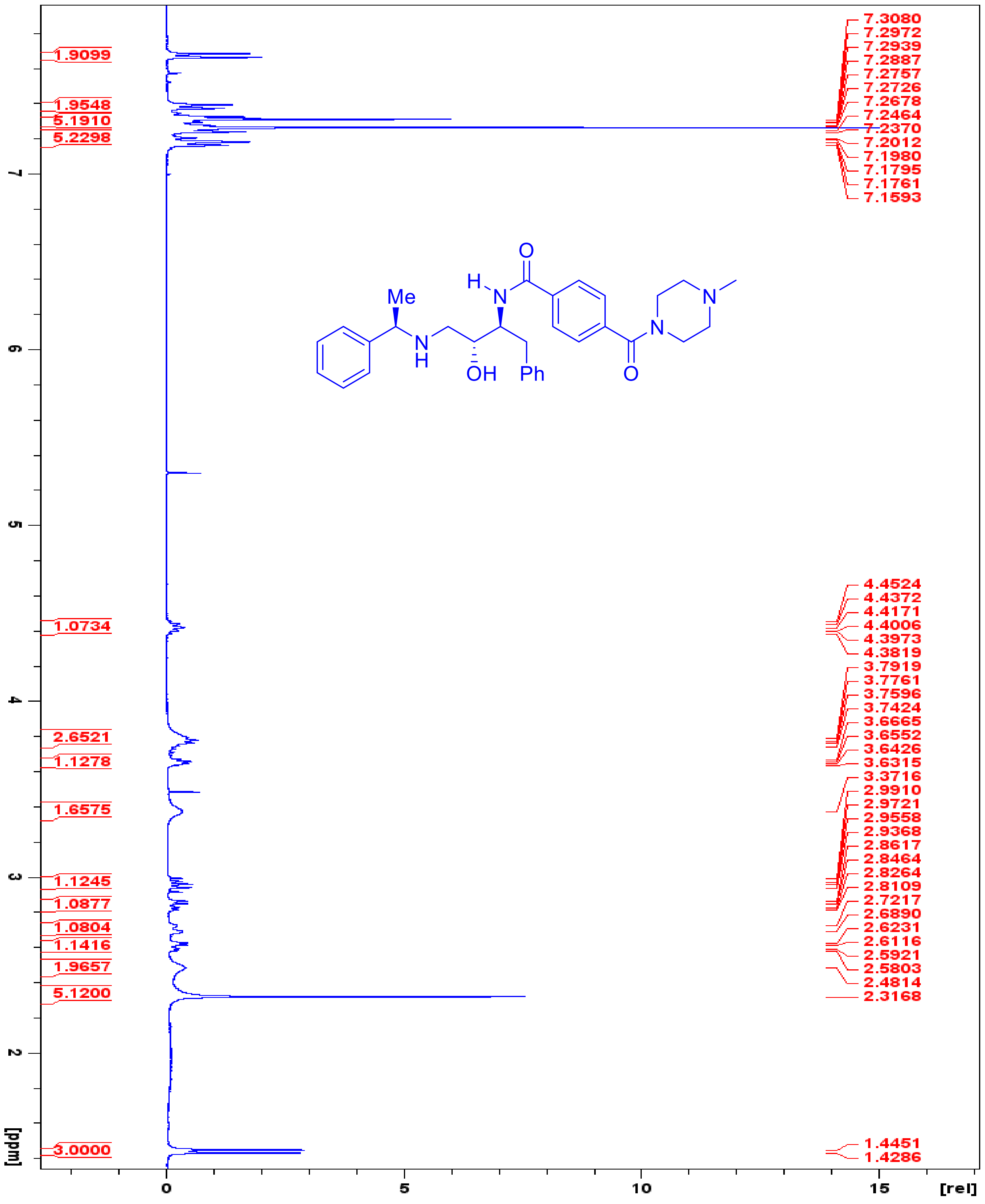

Figure S27. 400 MHz ^{1}H NMR Spectrum of **4** in $CDCl_3$.

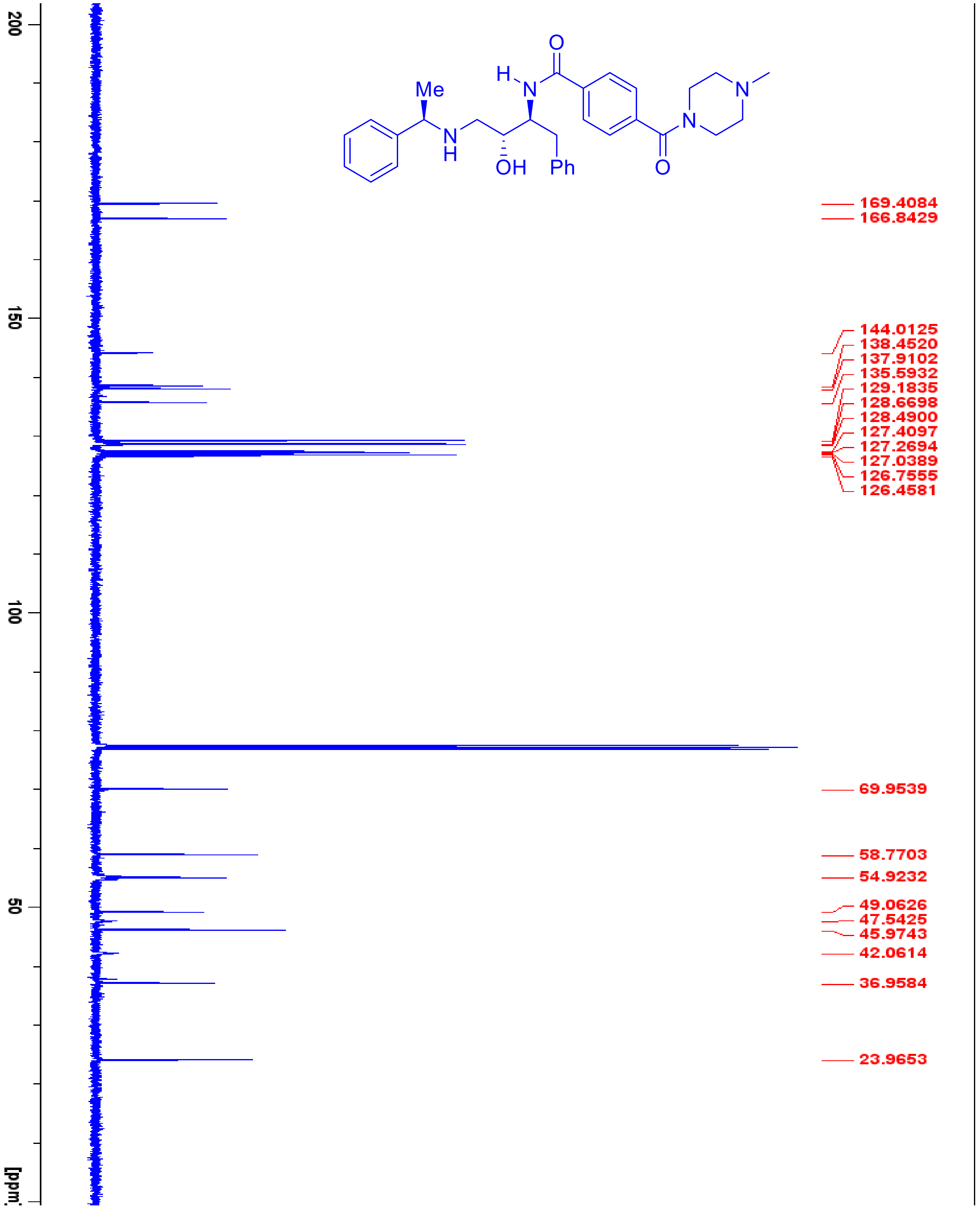

Figure S28. 100 MHz $^{13}C\{^{1}H\}$ NMR spectrum of **4** in $CDCl_3$.

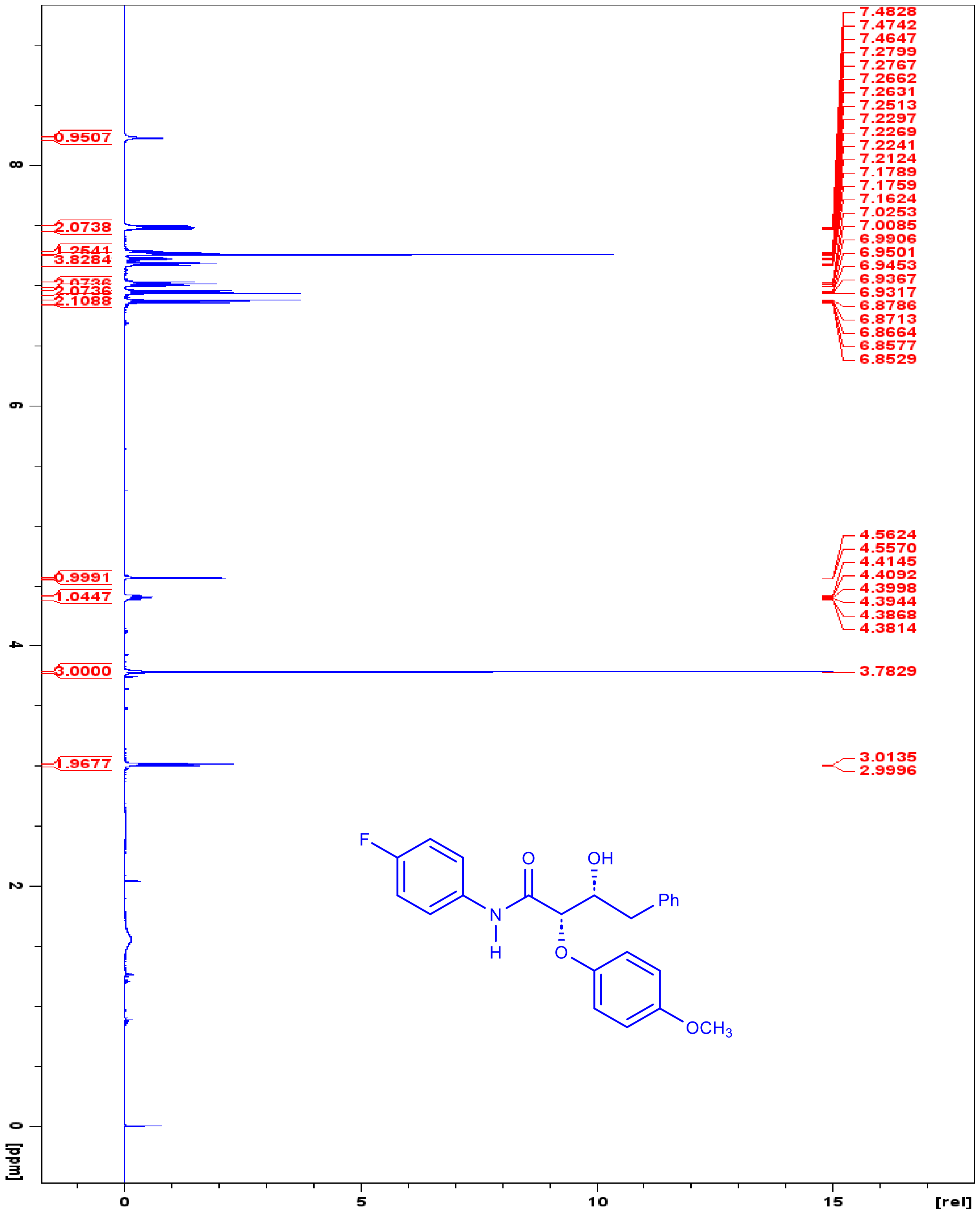

Figure S29. 500 MHz ^{1}H NMR Spectrum of **24** in $CDCl_3$.

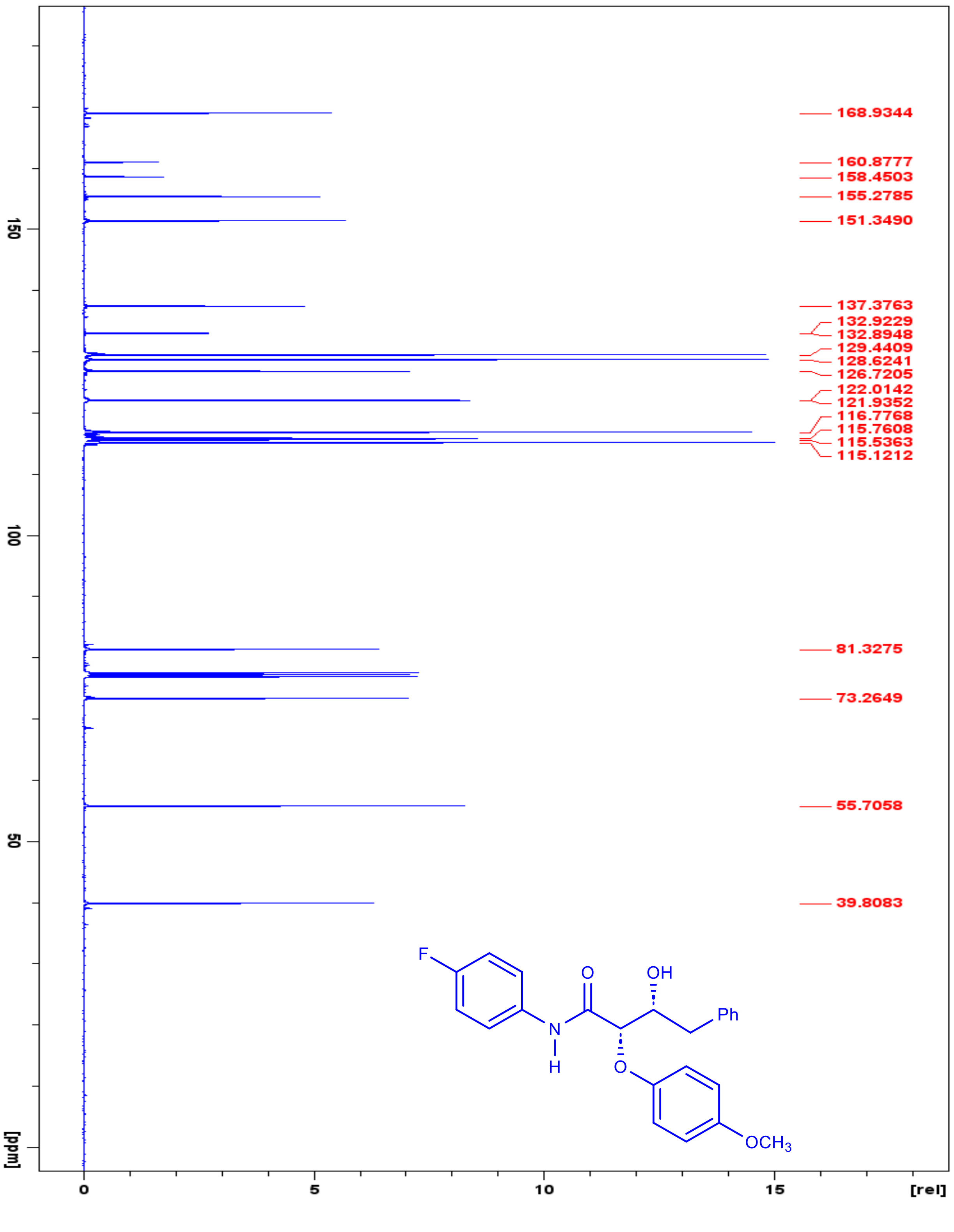

Figure S30. 100 MHz $^{13}C\{^{1}H\}$ NMR spectrum of **24** in $CDCl_3$.

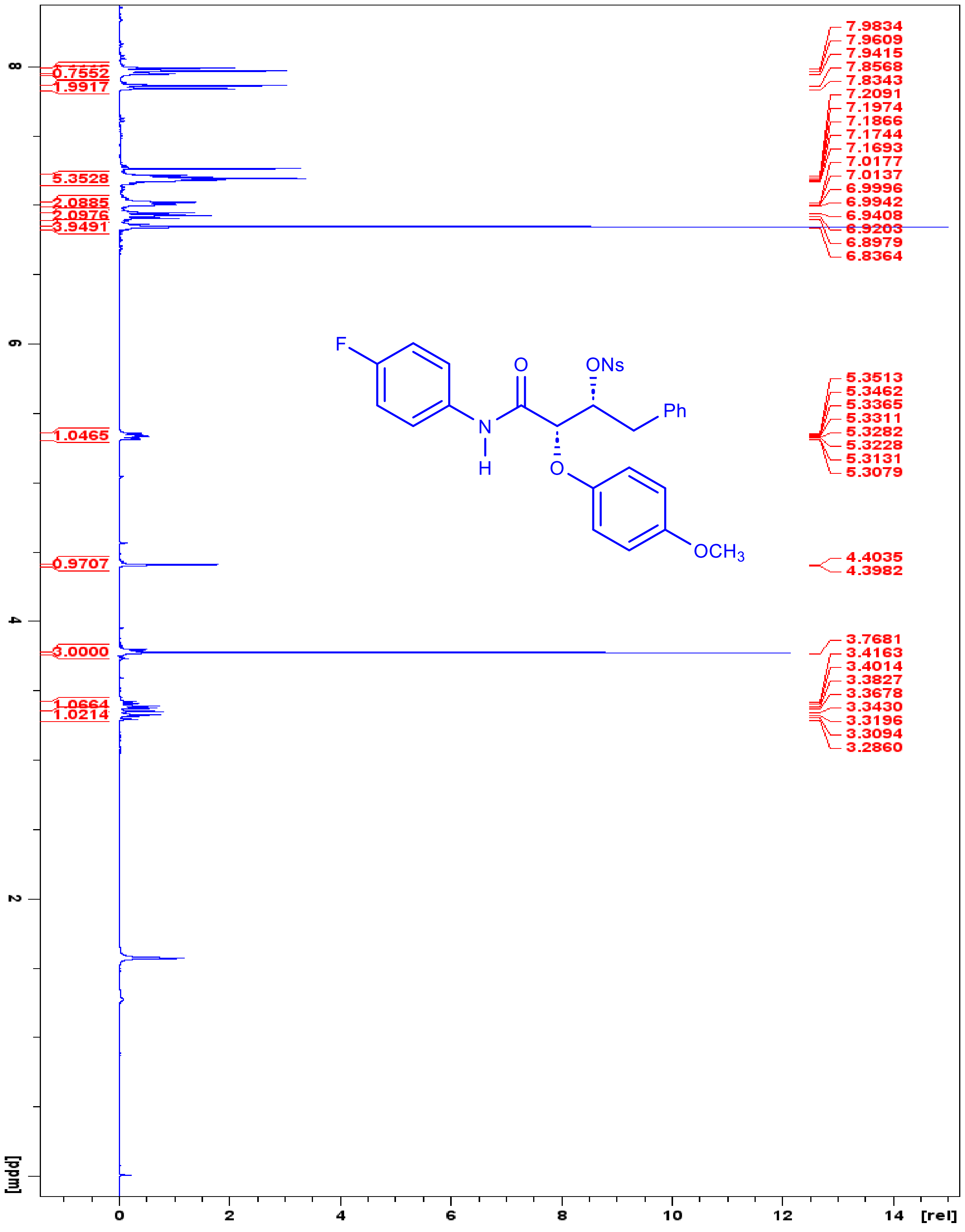

Figure S31. 400 MHz ^{1}H NMR Spectrum of **27** in $CDCl_3$.

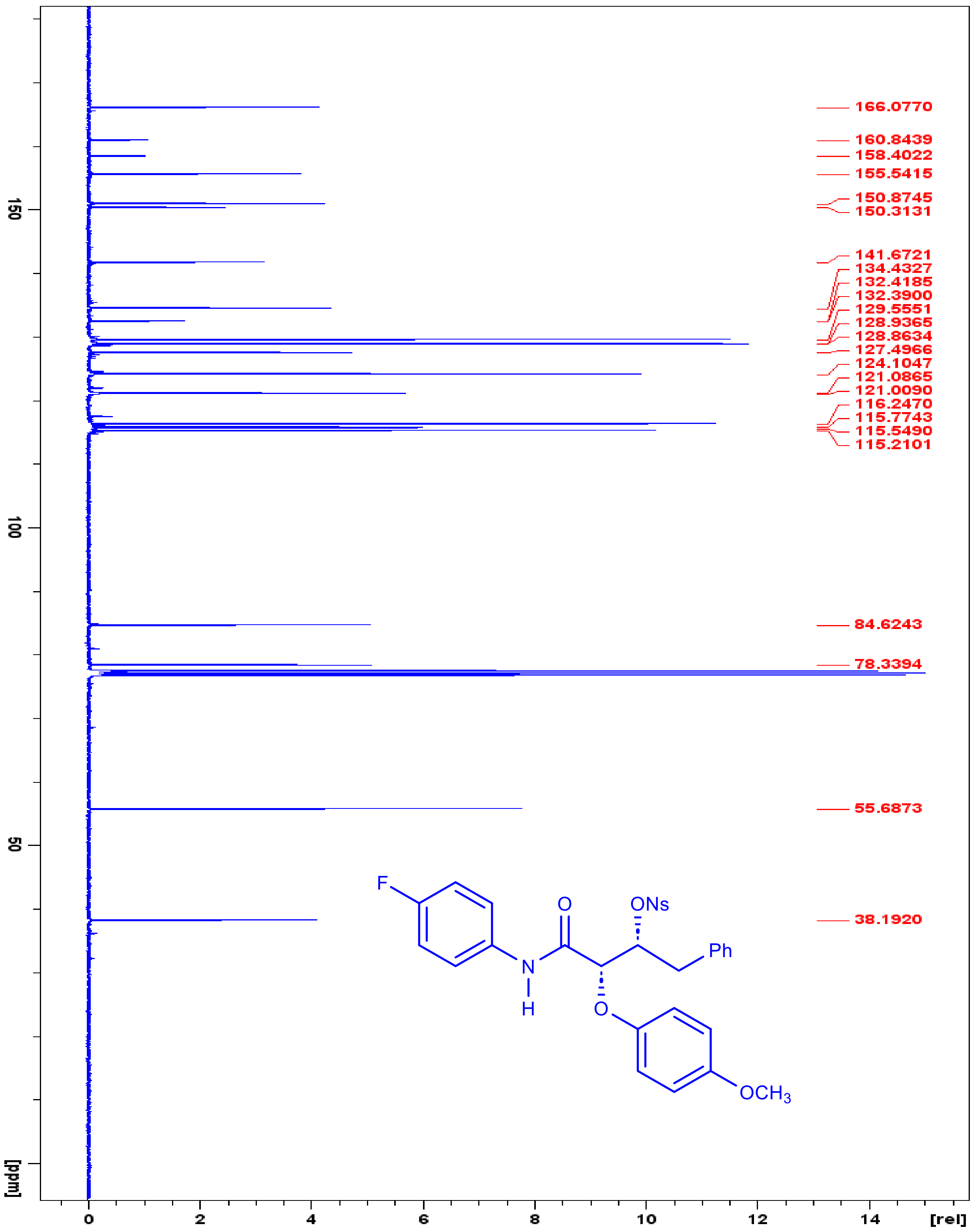

Figure S32. 100 MHz $^{13}C\{^{1}H\}$ NMR spectrum of **27** in $CDCl_3$.

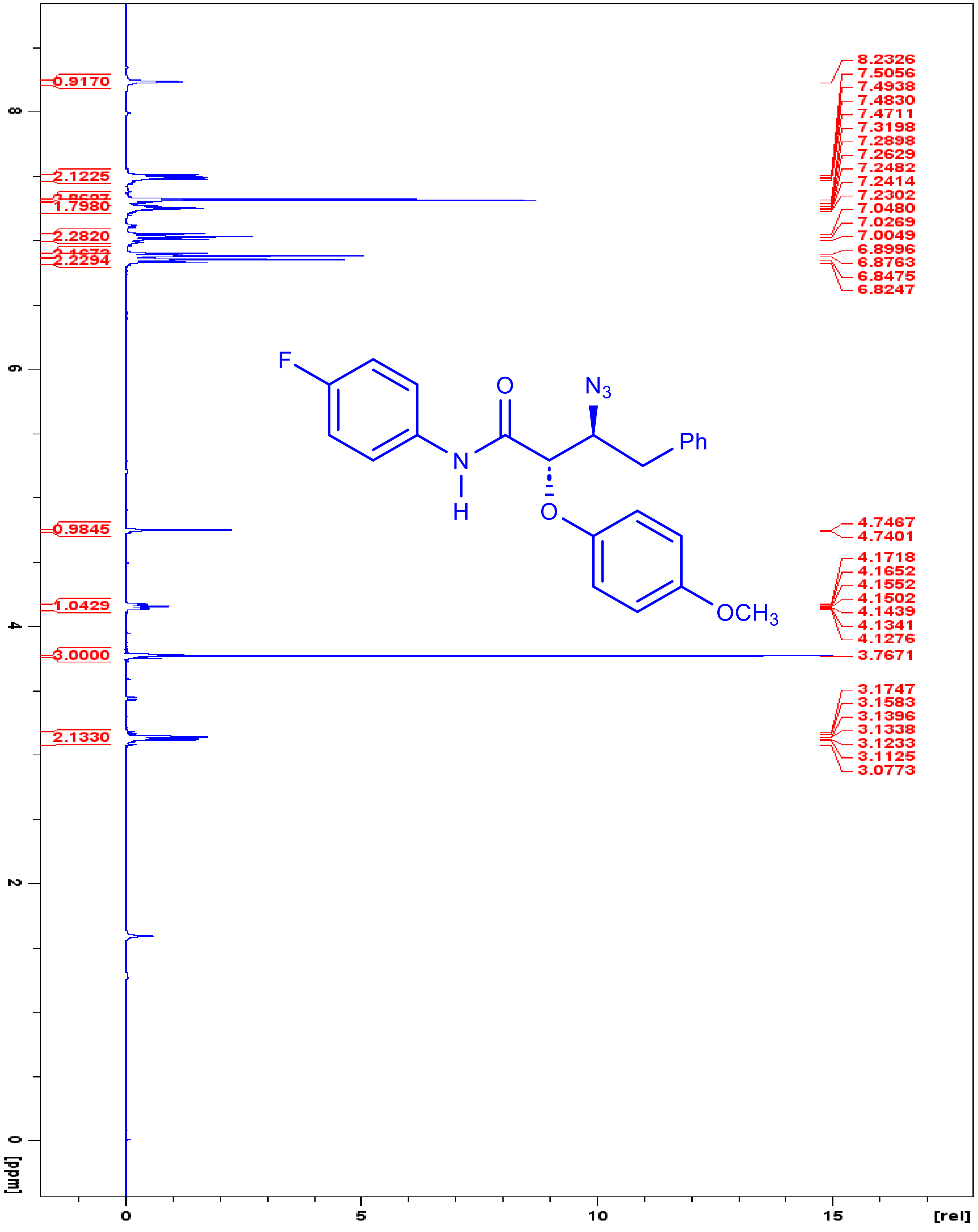

Figure S33. 400 MHz ^{1}H NMR Spectrum of **28** in $CDCl_3$.

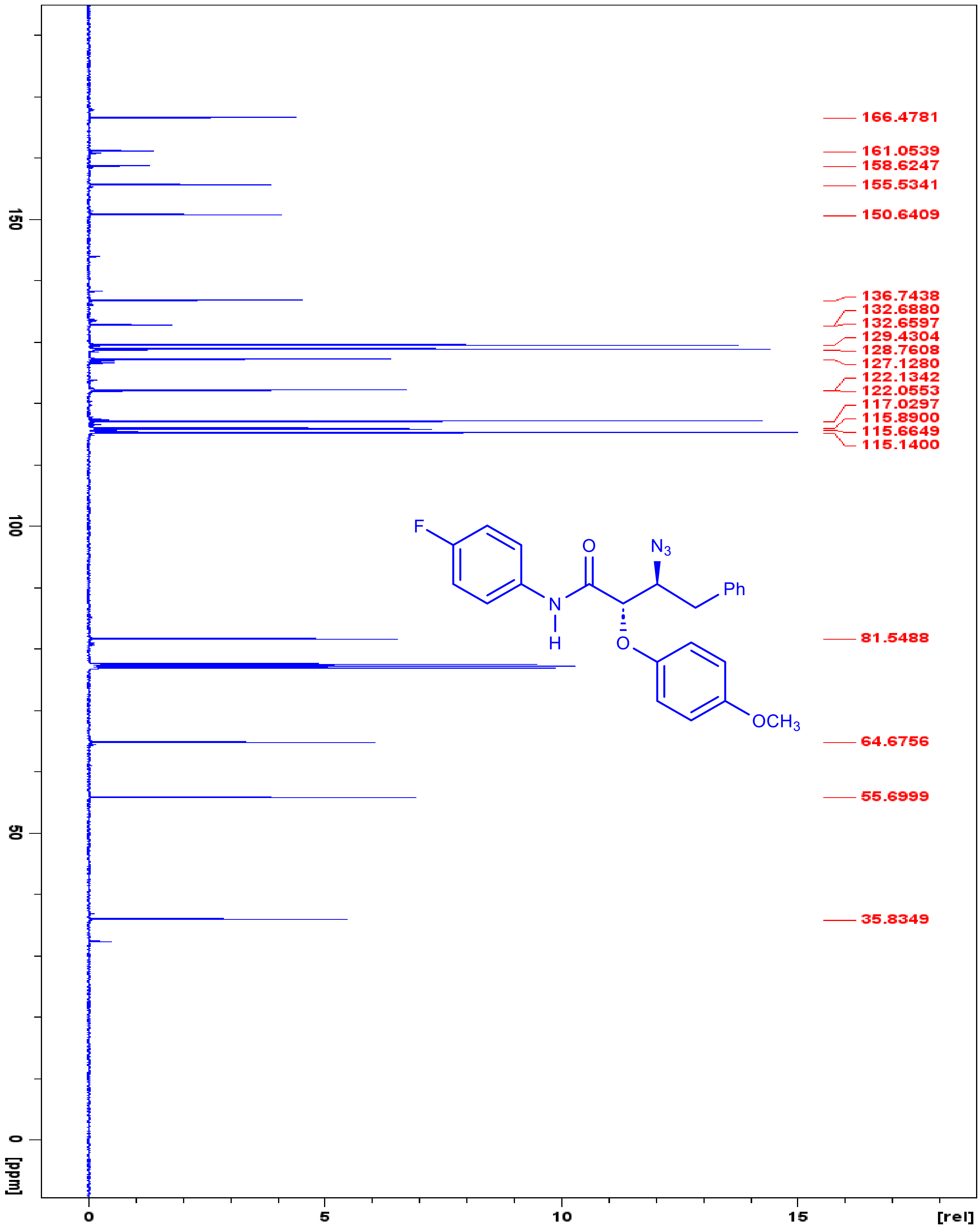

Figure S34. 100 MHz $^{13}C\{^1H\}$ NMR spectrum of **28** in $CDCl_3$.

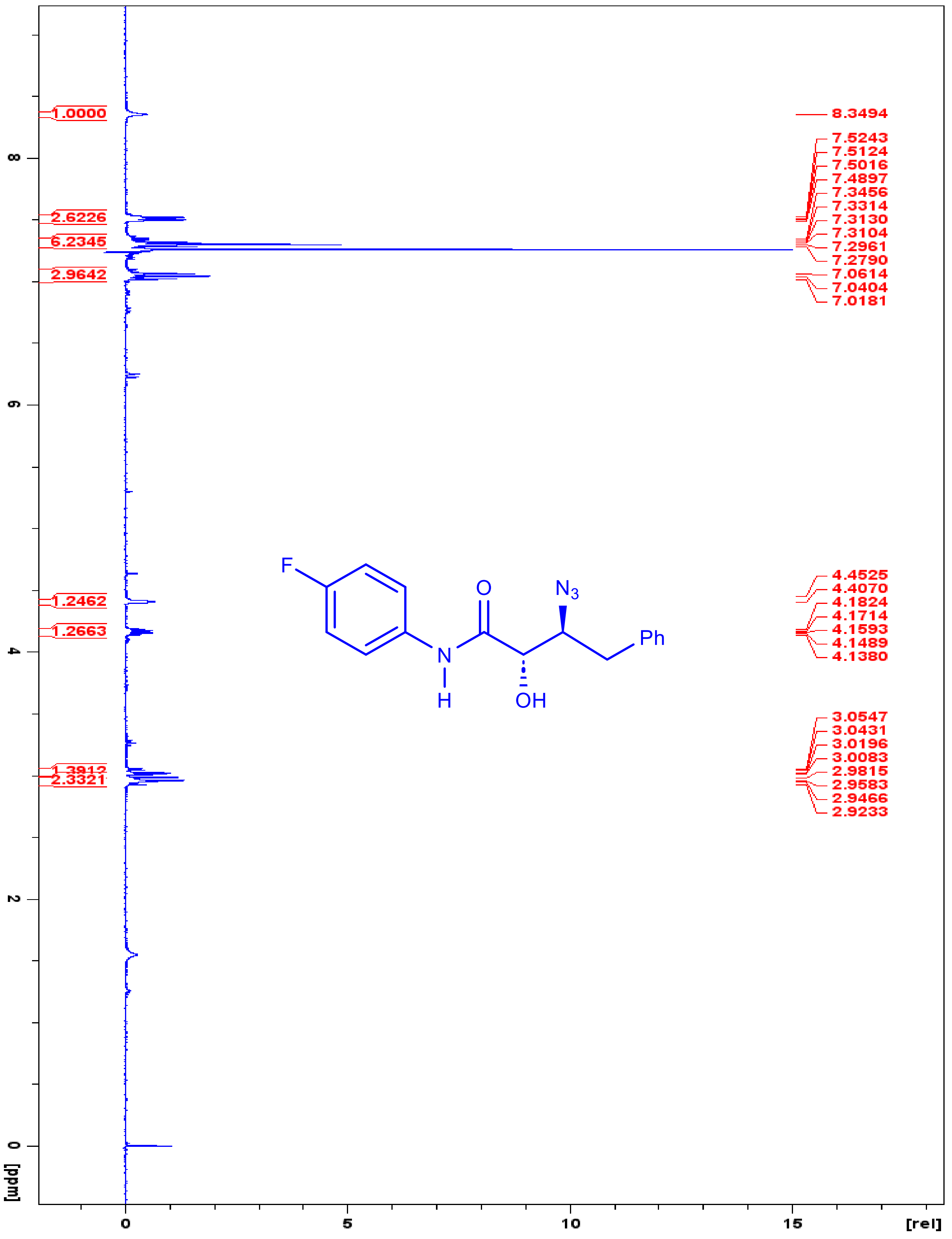

Figure S35. 400 MHz ^{1}H NMR Spectrum of **23** in $CDCl_3$.

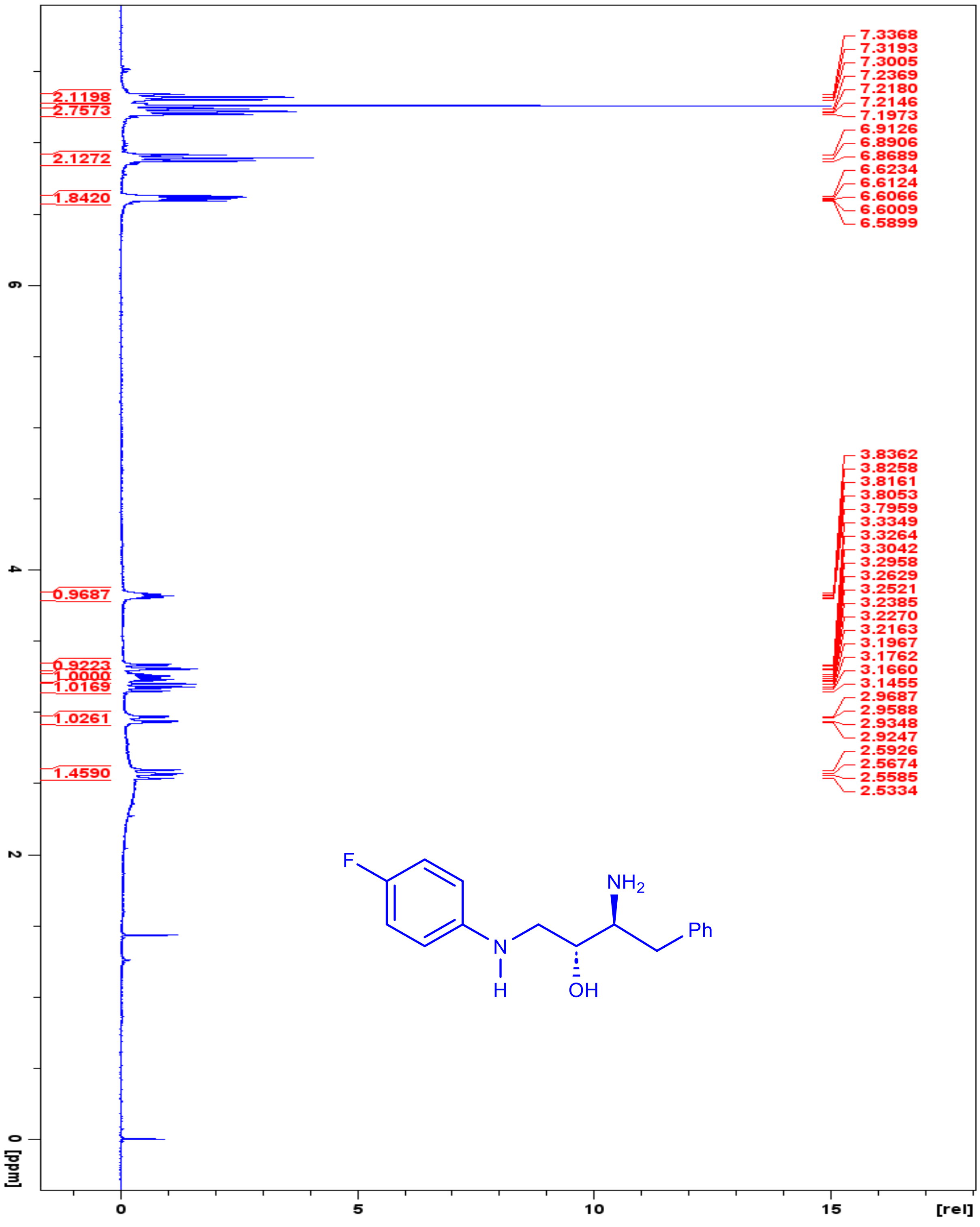

Figure S36. 400 MHz ^{1}H NMR Spectrum of **22** in $CDCl_3$.

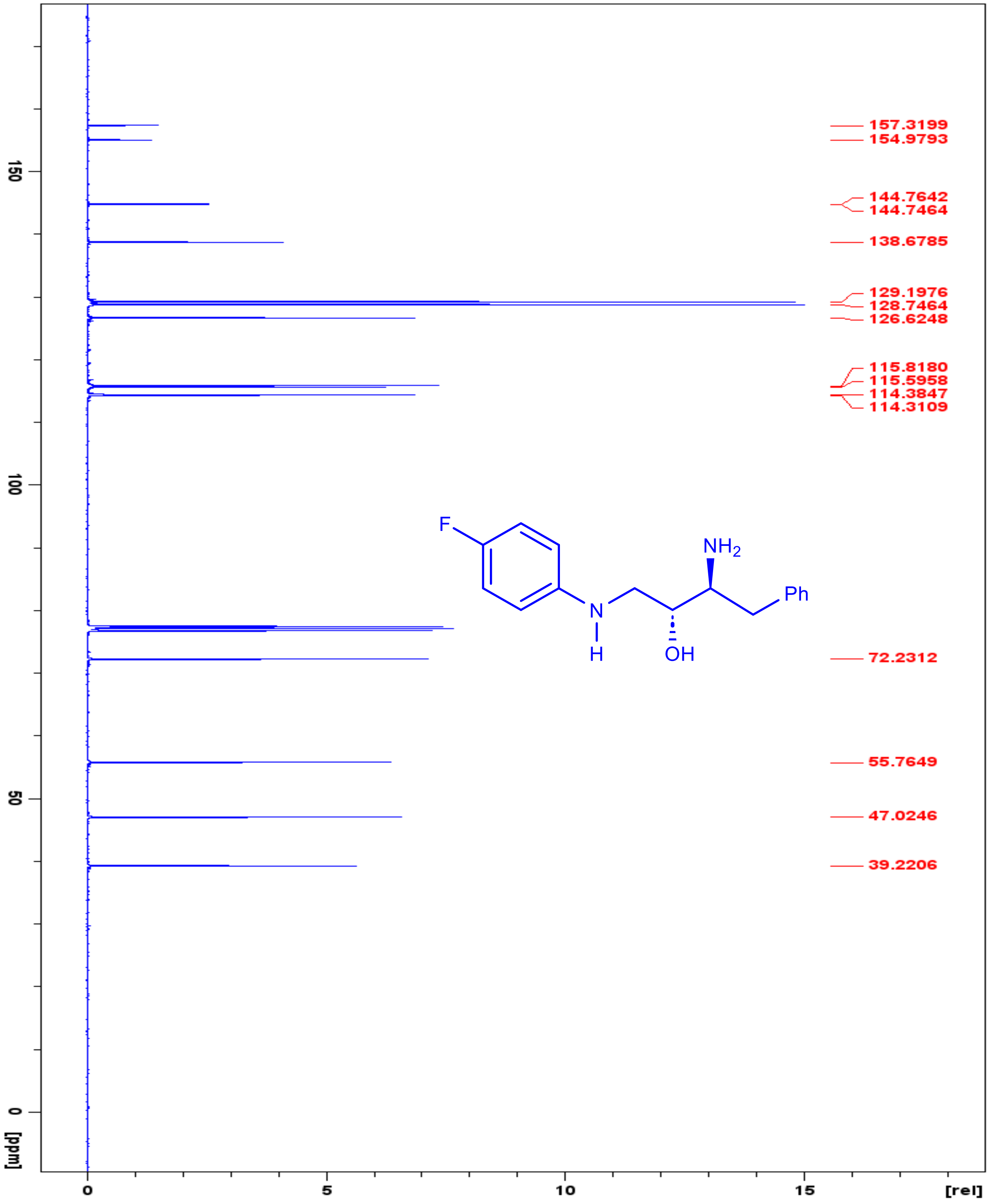

Figure S37. 100 MHz $^{13}C\{^{1}H\}$ NMR spectrum of **22** in $CDCl_3$.

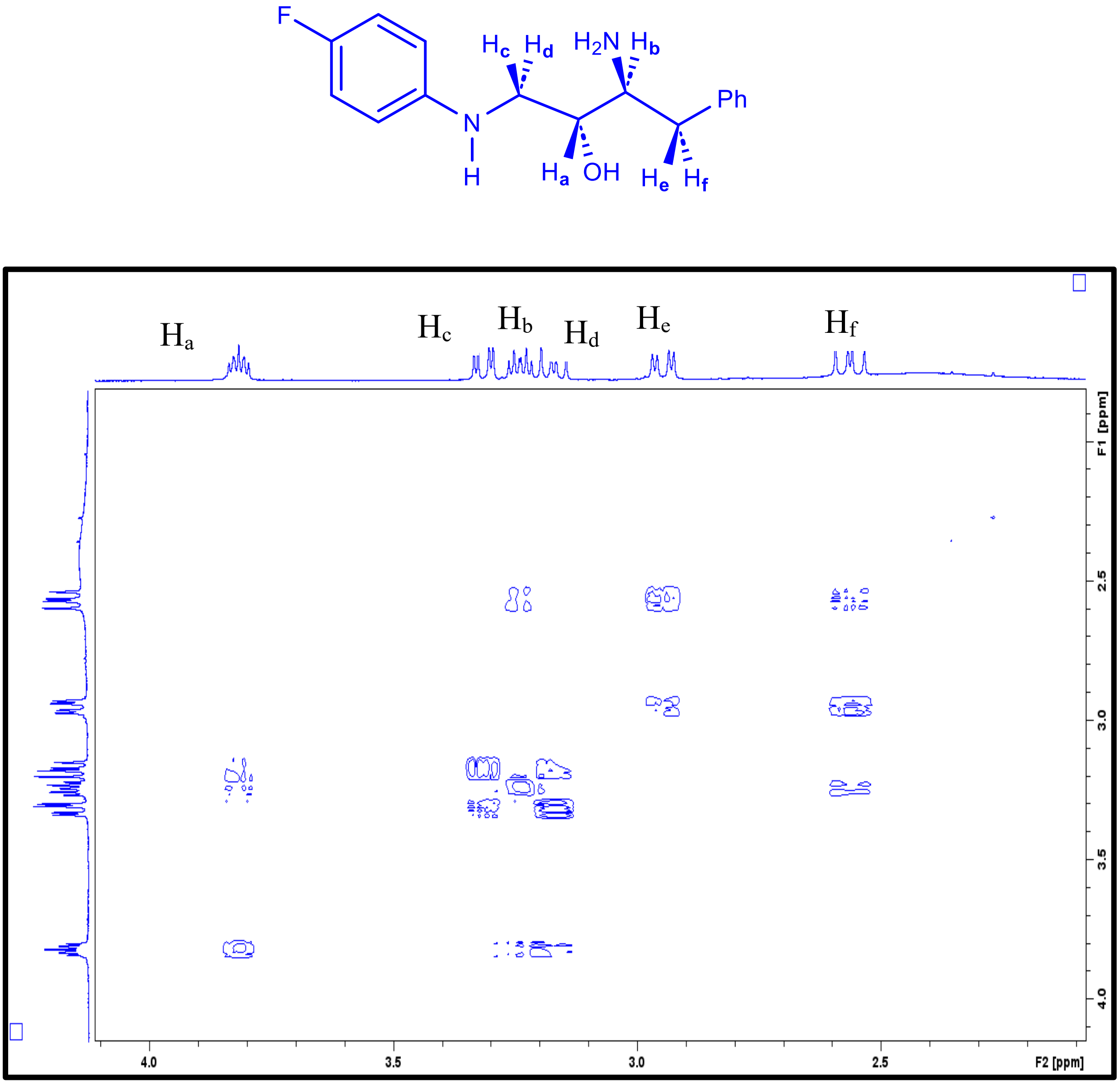

Figure S38. 500 MHz ^{1}H-^{1}H-COSY of **22** in $CDCl_3$.

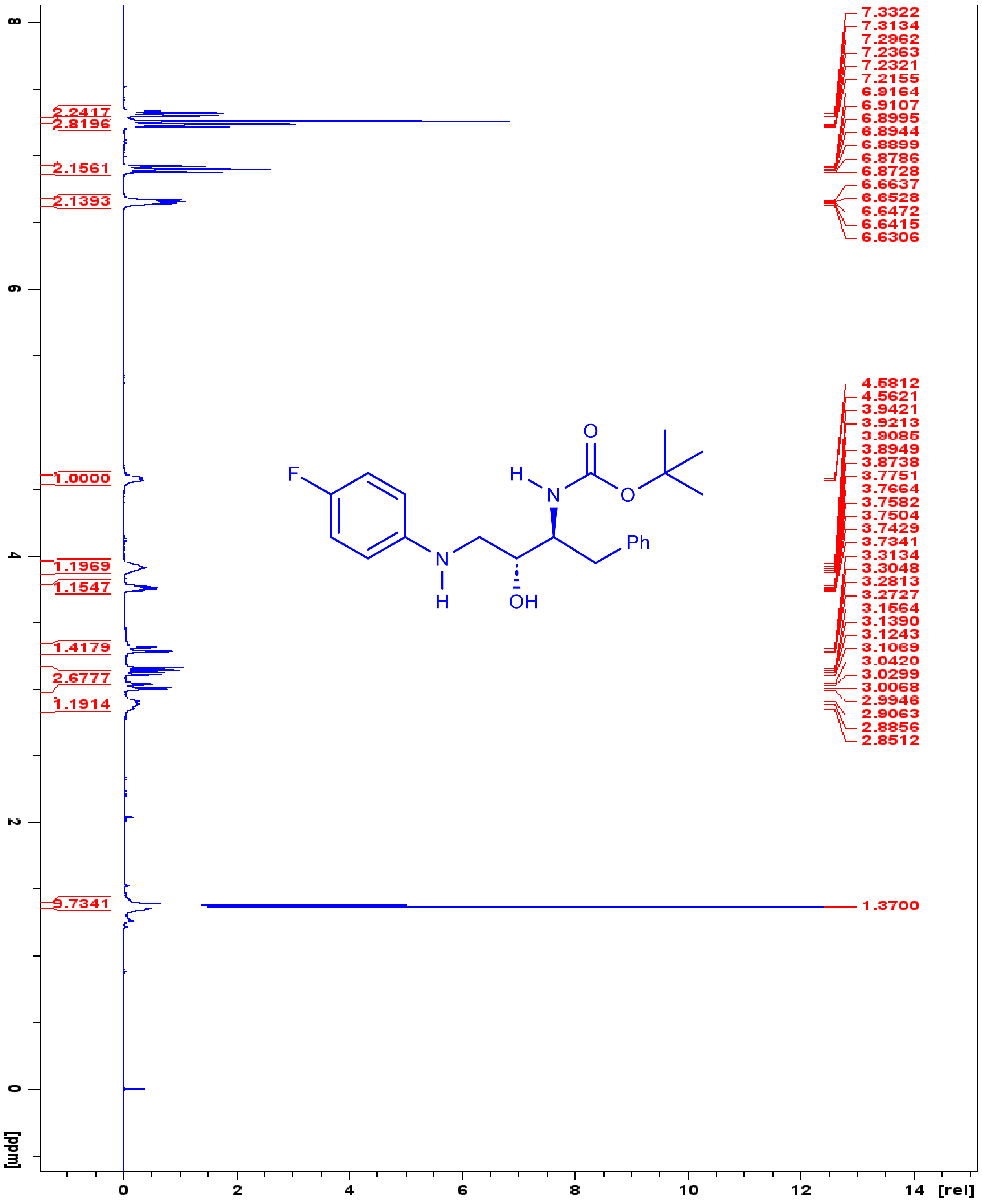

Figure S39. 400 MHz ^{1}H NMR Spectrum of **5** in $CDCl_3$.

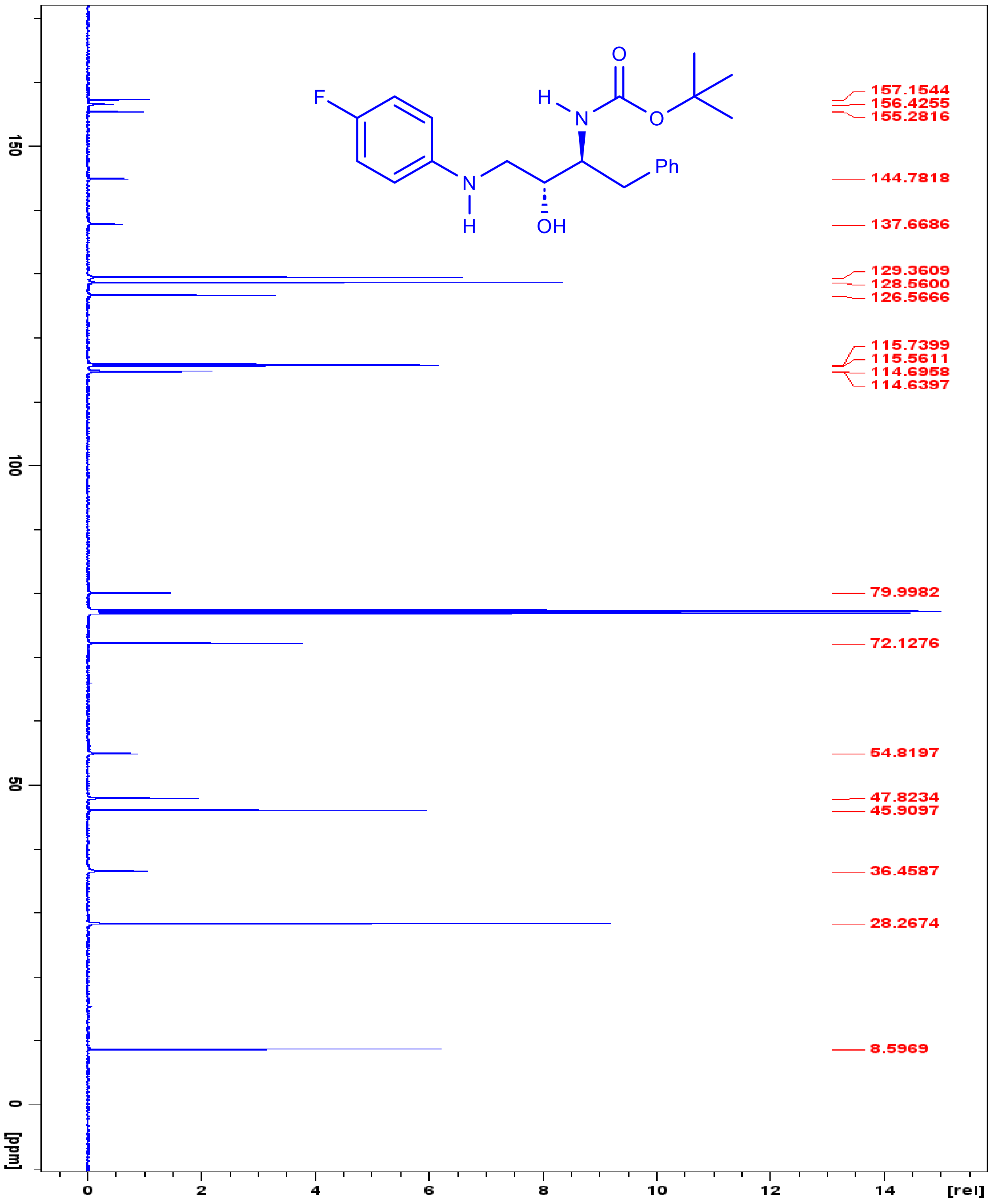

Figure S40. 100 MHz $^{13}C\{^{1}H\}$ NMR spectrum of **5** in $CDCl_3$.

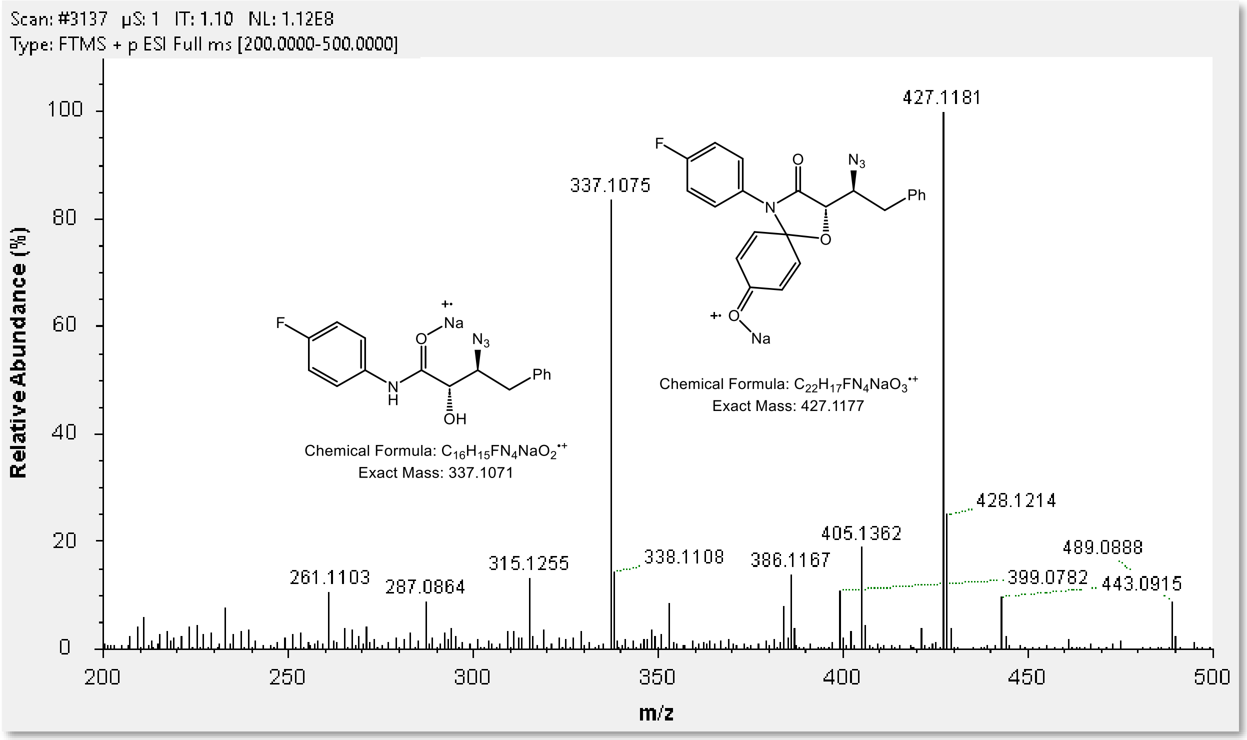

Figure S41. High resolution mass spectrum of the deprotection putative byproduct **32**.
